# Impact of Malaria Control on Mortality and Anemia among Tanzanian Children Less than Five Years of Age, 1999–2010

**DOI:** 10.1371/journal.pone.0141112

**Published:** 2015-11-04

**Authors:** Paul Smithson, Lia Florey, S. Rene Salgado, Christine L. Hershey, Honorati Masanja, Achuyt Bhattarai, Alex Mwita, Peter D. McElroy, Tanzania Malaria Impact Evaluation Research Group

**Affiliations:** 1 Ifakara Health Institute, Dar es Salaam, United Republic of Tanzania; 2 ICF International, Rockville, Maryland, United States of America; 3 United States Agency for International Development, U.S. President’s Malaria Initiative, Washington, DC, United States of America; 4 Centers for Disease Control and Prevention, U.S. President’s Malaria Initiative, Atlanta, Georgia, United States of America; 5 National Malaria Control Programme, Ministry of Health and Social Welfare, Dar es Salaam, United Republic of Tanzania; Centro de Pesquisa Rene Rachou/Fundação Oswaldo Cruz (Fiocruz-Minas), BRAZIL

## Abstract

**Background:**

Mainland Tanzania scaled up multiple malaria control interventions between 1999 and 2010. We evaluated whether, and to what extent, reductions in all-cause under-five child mortality (U5CM) tracked with malaria control intensification during this period.

**Methods:**

Four nationally representative household surveys permitted trend analysis for malaria intervention coverage, severe anemia (hemoglobin <8 g/dL) prevalence (SAP) among children 6–59 months, and U5CM rates stratified by background characteristics, age, and malaria endemicity. Prevalence of contextual factors (e.g., vaccination, nutrition) likely to influence U5CM were also assessed. Population attributable risk percentage (PAR%) estimates for malaria interventions and contextual factors that changed over time were used to estimate magnitude of impact on U5CM.

**Results:**

Household ownership of insecticide-treated nets (ITNs) rose from near zero in 1999 to 64% (95% CI, 61.7–65.2) in 2010. Intermittent preventive treatment of malaria in pregnancy reached 26% (95% CI, 23.6–28.0) by 2010. Sulfadoxine-pyrimethamine replaced chloroquine in 2002 and artemisinin-based combination therapy was introduced in 2007. SAP among children 6–59 months declined 50% between 2005 (11.1%; 95% CI, 10.0–12.3%) and 2010 (5.5%; 95% CI, 4.7–6.4%) and U5CM declined by 45% between baseline (1995–9) and endpoint (2005–9), from 148 to 81 deaths/1000 live births, respectively. Mortality declined 55% among children 1–23 months of age in higher malaria endemicity areas. A large reduction in U5CM was attributable to ITNs (PAR% = 11) with other malaria interventions adding further gains. Multiple contextual factors also contributed to survival gains.

**Conclusion:**

Marked declines in U5CM occurred in Tanzania between 1999 and 2010 with high impact from ITNs and ACTs. High-risk children (1–24 months of age in high malaria endemicity) experienced the greatest declines in mortality and SAP. Malaria control should remain a policy priority to sustain and further accelerate progress in child survival.

## Introduction

Malaria control in sub-Saharan Africa attained unprecedented momentum during the decade after 2000 [1–3]. Donor assistance for malaria control in Africa increased 10-fold between 2000 and 2010, from $200 million to nearly $2 billion annually [4]. Most support came after 2005 from national governments, the Global Fund to Fight AIDS, Tuberculosis and Malaria, the World Bank Booster Program, and the U.S. President’s Malaria Initiative. These resources permitted nationwide scale-up of highly effective malaria control interventions, including insecticide treated bednets (ITNs), indoor residual spraying (IRS) of household walls with insecticides, intermittent preventive treatment of malaria during pregnancy (IPTp), and artemisinin-based combination therapy (ACT) [4,5]. Numerous African countries are on track to meet the World Health Assembly target of a 75% reduction in malaria incidence by 2015 [2]. However, evaluations of the public health impact of malaria control at national scale are sparse [6–8].

**Fig 1:**
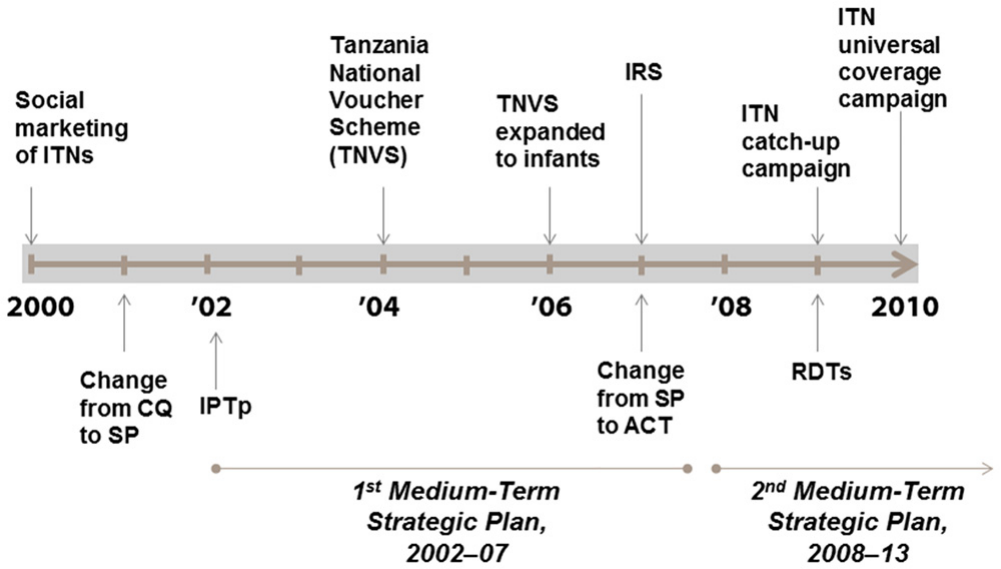
Timeline depicting years when national-level implementation of specific malaria control policies and interventions began in Mainland Tanzania, 2000–2010.

At least 97% of the Tanzania population lived in areas with intense perennial malaria transmission in 2000 [9,10]. Over the following decade, funding for malaria control increased dramatically—from less than $1 million in 2000 to more than $30 million in 2006 and nearly $140 million in 2010 –a cumulative investment of $452 million (S1A and S1B Fig). This funding facilitated the adoption and implementation of a series of malaria control policies and strategies (Fig 1) [11,12]. Social marketing of ITNs and insecticide treatment kits began in 2000 [13]. A year later, high levels of chloroquine resistance prompted a shift to sulfadoxine-pyrimethamine (SP) as first-line therapy for uncomplicated malaria [14,15]. In 2002, NMCP’s first *Medium-term Strategic Plan 2002–7* was implemented and IPTp using SP was adopted to control malaria in pregnancy. Large-scale ITN distribution began with the Tanzania National Voucher Scheme targeted to pregnant women [16] in 2004 and then infants in 2007 [17]. A second *Medium-term Strategic Plan 2008–13* called for an ACT (artemether-lumefantrine) as first-line therapy (from 2007), RDTs at peripheral health facilities (from 2009), and a target of 80% ITN coverage by 2010. Distribution of 8.6 million free long-lasting insecticidal nets (LLINs) to all children less than five years of age began in 2009 and was completed in 2010 [18]. Later that year a universal coverage campaign began to distribute 18 million free LLINs to cover all remaining sleeping spaces [19]. IRS was implemented in Kagera beginning in 2007, and advanced to Mwanza and Mara (three of the original 21 administrative regions) by 2010, but covered only 6% of the national population.

By 2010 malaria intervention coverage had been brought to levels where an impact may reasonably be expected. After a decade of stagnation, all-cause under-five child mortality (U5CM) declines were already evident by 2005 [20], and current UNICEF data indicate Tanzania likely achieved their *Millennium Development Goal 4* (MDG4) target of a two-thirds reduction in U5CM between 1990 and 2015: modeled rates of U5CM declined from 166 to 132 to 54 deaths per 1000 live births in 1990, 2000, and 2012, respectively (a 68% decline) [21].

The Roll Back Malaria (RBM) Partnership’s Monitoring and Evaluation Reference Group (MERG) recommends U5CM as the primary indicator of malaria control impact [22]. This recommendation is based on our: 1) inability to measure malaria-specific mortality in most developing countries, 2) expectation that rapid scale-up of interventions to high levels will result in measurable declines in U5CM, and 3) recognition that malaria has a substantial indirect effect on mortality that will be captured in U5CM estimates [23]. The MERG approach assumes that a portion of the declining trends in U5CM can be plausibly attributed to malaria control efforts if improvements in population-level measurements are found in steps along the causal pathway (e.g., increased ITN coverage and reduced severe anemia prevalence) [24]. As U5CM rates and severe anemia prevalence decline at an accelerated pace, preceded by rapid scale-up of intervention coverage, the conclusion that malaria control efforts reduced malaria-specific mortality becomes more plausible. This plausibility is strengthened if trends in other contextual factors (e.g., immunization, micronutrient supplementation, climate) affecting mortality did not appreciably change during the same period.

Our objective was a summative evaluation to determine if Tanzania’s scale-up of malaria interventions during 1999–2010 helped accelerate declines in malaria morbidity (particularly severe anemia, a common manifestation of *Plasmodium falciparum* infection in high transmission areas [25,26]) and U5CM. The evaluation is restricted to Tanzania Mainland since Zanzibar has a separate Ministry of Health and its National Malaria Control Program followed a different trajectory.

## Methods

All surveys were approved by the ethical review board of Tanzania's National Institute for Medical Research and ICF International's institutional review board. Verbal informed consent was sought from all survey respondents, with an informed consent statement signed by each survey interviewer. All analyses were performed on anonymized and de-identified datasets.

### Evaluation design

**Fig 2:**
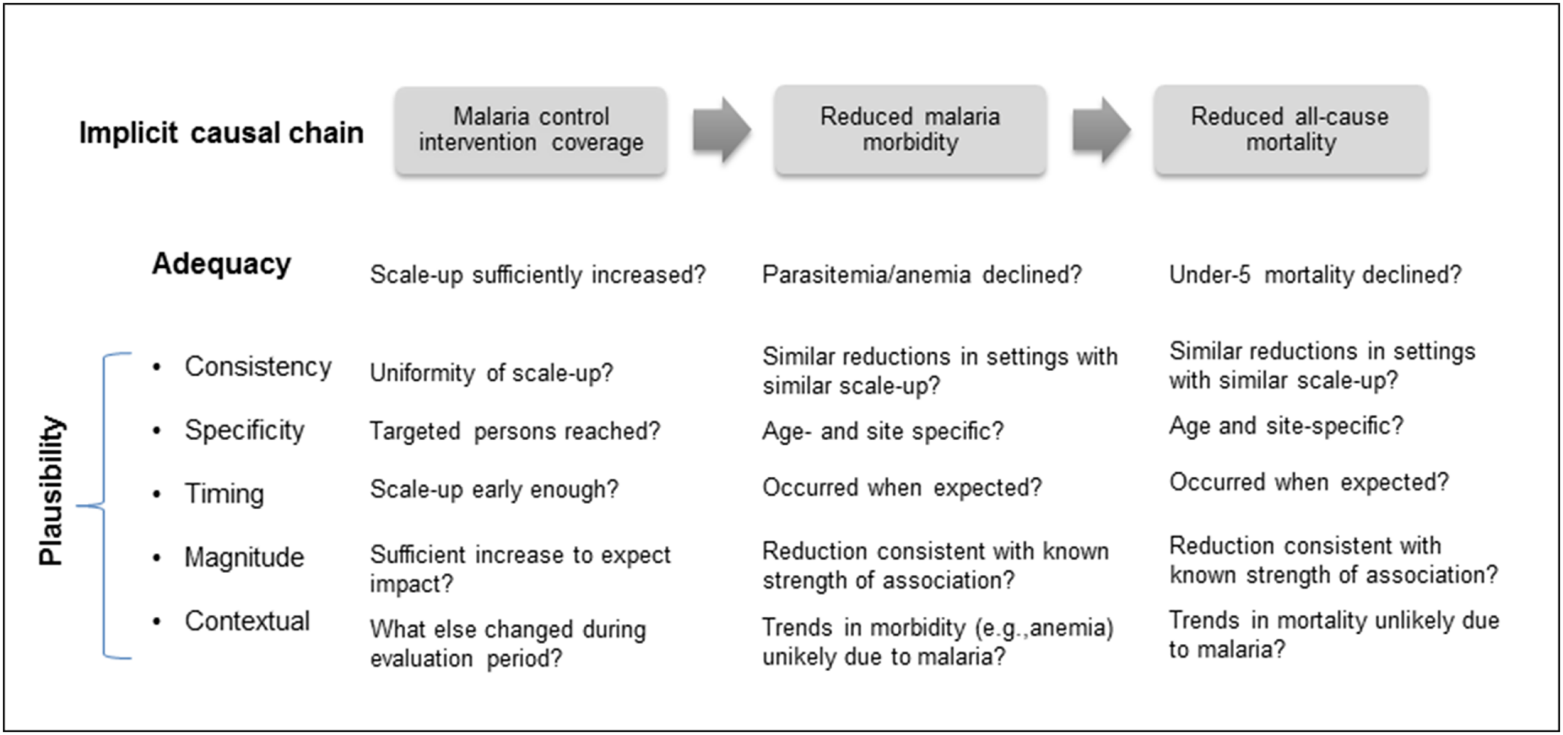
Conceptual framework for constructing an adequacy and plausibility assessment to support the Mainland Tanzania malaria impact evaluation.

Our overall evaluation design embraced both *adequacy* and *plausibility* aspects (Fig 2) [24]. Our initial *adequacy assessment* examined temporal trends in malaria intervention coverage and whether declines in severe anemia prevalence (SAP) and U5CM were observed. This step also explored inequities in malaria intervention coverage across four basic demographic characteristics and whether the same inequities existed for SAP and U5CM rates. Adequacy assessments lack comparison groups and do not address non-malarial contextual factors thought to affect the impact indicators of interest [24]. The *plausibility assessment* included a series of analyses to complement the adequacy assessment and garner greater confidence that a portion of the reductions in SAP and U5CM were due to malaria intervention scale-up [27]. The plausibility assessment stems from Hill’s specificity criteria [28]. This effort contributed to the consistency and specificity of our plausibility assessment. We assessed whether malaria control led to more accelerated declines in SAP and U5CM in younger (<23 months) versus older (24–59 months) children, and among those living in higher versus lower areas of malaria endemicity. Trends in prevalence of other contextual factors with recognized potential to contribute alternative explanations for changes in U5CM were explored. Finally, we examined whether the increase in malaria intervention coverage was compatible with the timing and magnitude of reductions in SAP and U5CM, while also considering changes in relevant contextual factors.

### Data sources and indicators

Malaria intervention coverage, malaria parasitemia, SAP, all-cause mortality, demographic characteristics, and coverage of other health interventions were obtained from four, nationally-representative household surveys in 1999, 2004/5, 2007/8, and 2010 that included 2747, 7847, 6332 and 7720 mainland households, respectively [29–32]. Each survey included a sampling design that was 100% inclusive of all 20 regions, plus Dar es Salaam. All surveys occurred during the drier season (Sep-Feb), but fieldwork for the 2010 survey extended three months longer. Malaria intervention coverage was measured using six standard RBM indicators [33] including the proportion of: households that owned at least one ITN; children <5 years of age, pregnant women, and *de facto* survey population who slept under an ITN the night before the survey; women who received IPTp-2 for pregnancies within the past two years; and children <5 years of age with fever in the past two weeks who received treatment with a first-line antimalarial the same or next day after fever onset. Capillary blood specimens were obtained via finger or heel prick from children 6–59 months of age who slept in each household the night before the survey. Hemoglobin (Hb) concentration was measured (*HemoCue* ^®^ instrument) and severe anemia defined as Hb < 8.0 g/dL, adjusted for altitude [34,35]. A histidine-rich protein 2-based RDT (*Paracheck* ^®^) detected *P*. *falciparum* parasitemia (2007/8 survey only).

Contextual factors (socio-economic, maternal and reproductive health, nutrition, immunization, other morbidities) were sourced from the same surveys described above [29–32]. Additional published sources are cited for trends in rainfall, growth in gross domestic product (GDP) [36], and HIV/AIDS indicators [37–39]. Rainfall data (1983–2010) were obtained from approximately 200 ground-based stations of the Tanzania National Meteorological Agency. Minimum/maximum temperatures were generated by combining weather station measurements with NASA’s land surface temperature estimates with a digital elevation model.

### Analyses

#### Trends in intervention coverage, SAP and U5CM

For each survey, RBM core coverage indicators were calculated with corresponding 95% confidence intervals (CIs) using standard errors corrected for multi-stage survey design [34]. SAP estimates with 95% CIs were calculated for children 6–59 months of age. Direct estimates of U5CM and 95% CIs were calculated with the synthetic cohort life table approach using birth histories over 0–4 years prior to each survey. A chi-square test for linear trend in proportions assessed changes in intervention coverage, SAP and U5CM. ITN use, SAP, and U5CM were further stratified by four key demographic characteristics including sex (male/female), residence (urban/rural), wealth quintile, maternal education (none, primary incomplete, primary complete, secondary+) and plotted with corresponding 95% CIs to assess trends in equity. Under-five, infant, and neonatal mortality rates with 95% CIs were plotted to reflect temporal trends from pre-baseline [40,41] through endpoint.

#### Plausibility assessment (1): Temporal trends by age

Malaria morbidity and mortality are highly age-dependent, with younger children (<24 months) at greater risk for both outcomes, particularly in areas with higher intensity *P*. *falciparum* transmission [42–44]. SAP was stratified by age (6–23 and 24–59 months). Further mortality analyses excluded neonatal deaths since a minimal proportion of these deaths were likely attributable to malaria [45]. Thus all-cause mortality was dichotomized at 1–23 and 24–59 months of age.

#### Plausibility assessment (2): Temporal trends by malaria endemicity

SAP and mortality rates were stratified by level of malaria endemicity to assess whether greater impact was observed in higher endemicity areas. Tanzania mainland regions were divided into three endemicity categories: “lower” (7 regions, prevalence 0–9.2%), “moderate” (7 regions, prevalence 10.4–21.9%), and “higher” (6 regions, prevalence 28.7–32.6%) using region-specific *P*. *falciparum* prevalence from 2007/8 (the only national survey to include this indicator). Survey-specific adjusted odd ratios (ORs) for severe anemia and rate ratios (RRs) for all-cause mortality were obtained using logistic and Cox proportional hazards regression models, respectively, to control for the four key demographic characteristics described above, malaria endemicity level, and age (logistic model only).

#### Plausibility assessment (3): Temporal trends by age and malaria endemicity combined

Age and malaria endemicity exhibited differential risk for SAP and all-cause child mortality. Trends in these outcomes were assessed separately for children 1–23 months of age and children 24–59 months of age, stratified by endemicity.

#### Plausibility assessment (4): Accounting for contextual factors

Climate data were examined in a three-step analysis (IRI Report, supplemental online material). The Climate Suitability for Malaria Transmission Tool [46] was first used to estimate the number of months during the year when climatological conditions were suitable for malaria transmission. A Weighted Anomaly of Standardized Precipitation (WASP) Tool [47,48] estimated the change in rainfall over the evaluation period relative to baseline (1995–99). WASP and temperature data were combined to assess whether the prevailing climate in Tanzania was suitable for malaria transmission relative to baseline. The Climate Analysis Tool explored annual precipitation and temperature data according to temporal and spatial factors affecting malaria transmission. Change in per capita GDP (constant prices) was expressed as cumulative change between 1995–1999 and 2005–2009. For all other contextual factors, baseline (1999) and endpoint (2010) values with 95% CIs and percent changes (relative) were obtained. Differences in baseline and endpoint values were assessed with a Z-test using the standard errors in the Z-score formula. For a small subset of factors lacking 1999 and 2010 values (e.g., HIV/AIDS indicators) the earliest and latest available estimates were used for baseline and endpoint.

#### Plausibility assessment (5): Magnitude of reductions in all-cause mortality

We derived the attributable changes in U5CM due to the observed increases in malaria intervention coverage between 1999 and 2010 and for contextual factors with significant change over the same period. These reductions were derived using population attributable risk percentage (PAR%) calculations based on prevalence of exposure (E) and non-exposure (Ē) to each factor in 1999 and 2010, respectively, and published estimates of relative risk (RR) for all-cause mortality associated with that exposure factor [49–59]. The published RR estimate for effective first-line antimalarials (specifically ACTs) used malaria-specific mortality rather than all-cause mortality. The unadjusted PAR% is useful for comparing the crude marginal effects of multiple interventions and was calculated as:

$$PAR\%\;\;=\;\;{Ē}_{2010}\;\;+\;\;RR\left(E_{2010}\right)\;–\;\;\left[{Ē}_{1999}\;+\;RR\left(E_{1999}\right)\right]\;\;/\;\;Ē{\;}_{1999}\;+\;\;RR\left(E_{1999}\right)$$

[60].

It is an estimate of how the change in prevalence of each risk factor (intervention or exposure) and known strength of the association with mortality resulted in either increases or decreases in rates of U5CM. The PAR% provides an informative link between causality and public health action [61,62].

## Results

### Adequacy assessment: Trends in intervention coverage and rates of SAP and U5CM

Malaria intervention coverage generally improved between 1999 and 2010 (Table 1). Household ownership of ITNs and ITN use by children less than 5 years of age, pregnant women, and the *de facto* population as a whole increased by 61, 62, 55, and approximately 45 percentage points, respectively. A marked increase in ITN coverage between 2007/8 and 2010 followed the distribution of 18 million free ITNs to children less than 5 years of age (May 2009–May 2010). The proportion of pregnant women who received at least two doses of SP for IPTp increased from 21% in 2005 to 26% in 2010 (p<0.0001). During 1999–2010, a high percentage (53% in 1999 and 60% in 2010) of children with fever in the two weeks preceding the household survey received antimalarial treatment. The percentage of children who received a first-line antimalarial the same or next day following fever onset did not exceed 27% and showed no consistent trend.

**Table 1:** Trends in malaria intervention coverage indicators, Mainland Tanzania, 1999–2010.

Variable††	1999	2004/5	2007/8	2010		
	%	%	%	%		
	(95% CI)	(95% CI)	(95% CI)	(95% CI)		
	N	N	N	N	Change (abs.)*	*p* ^†^
**Nets**						
Household ownership ≥1	29.8	45.9	55.6	74.7	44.9	<0.0001
	(25.6–34.3)	(43.1–48.6)	(52.9–58.2)	(72.8–76.4)		
	3,526	9,483	8,269	9,377		
**ITNs**						
Household ownership ≥1	~2.4	22.5	38.3	63.5	61.1	<0.0001
	-	(20.5–24.6)	(36.1–40.6)	(61.7–65.2)		
	3,526	9,483	8,269	9,377		
Use (children <5 yrs)	~1.7	15.8	24.8	63.9	62.2	<0.0001
	(1.2–2.8)	(13.9–18.0)	(22.6–27.2)	(61.2–66.5)		
	3,495	8,147	7,319	7,768		
Use (pregnant women)	~1.9	15.7	26.0	57.1	55.2	<0.001
	(0.8–4.6)	(12.9–19.1)	(21.7–30.9)	(52.4–61.6)		
	369	1,054	823	922		
Use (all persons)	n/a**	14.9	19.7	45.1	~45	<0.0005
		(13.3–16.7)	(17.8–21.6)	(43.4–46.9)		
		44,830	40,660	45,125		
**IPTp during last pregnancy**						
2+ doses	n/a	20.8	29.6	25.7	25.7	<0.0001
		(19.0–22.7)	(27.0–32.3)	(23.6–28.0)		
		3,415	2,969	3,179		
1+ dose	n/a	50.1	56.9	59.9	59.9	<0.0001
		(47.1–53.1)	(53.5–60.3)	(56.8–62.9)		
		3,415	2,969	3,179		
**Malaria treatment**						
Any antimalarial (timing not considered)	53.0	58.2	57.0	60.1	7.1	0.2347
	(45.2–60.6)	(54.7–61.5)	(52.4–61.6)	(56.7–63.4)		
	988	1,882	1,320	1,715		
Recommended first-line (same or next day) ^‡^	n/a	21.4	14.2	26.7	5.3‡	0.0002
		(18.7–24.2)	(11.6–17.2)	(23.8–29.8)		
		1,882	1,320	1,715		

* Percent absolute change, earliest value vs. 2010

^†^ P-value obtained from chi^2^ test for linear trend in proportions across the four surveys (trend test begins with 2004/5 for indicators with “n/a” in 1999)

^‡^ First-line therapy in 1999: chloroquine; 2004/5: sulfadoxine/pyrimethamine; 2007/8 and 2010: ACT

** No ITN indicators were obtained in the 1999 (baseline) survey since these indicators were only developed and promoted by RBM after 2002. Consequently, the 1999 survey questionnaire did not include a full roster of nets, net treatment and use. All families were questioned about the number of nets they owned and the number of children <5 years of age per household was recorded, but net treatment (soaking in insecticidal solution) was asked only for households with children <5 years of age where some or all children slept under nets. The data available permitted a precise estimate of household net ownership; a fairly precise estimate of net and ITN use by children under five, and an estimate of household ITN ownership using available data on proportion of nets that were treated.

^††^N in the table represents weighted denominator (number of households, children under 5 years of age, pregnant women, all persons, or febrile children under 5 years of age) for each variable shown

**Fig 3:**
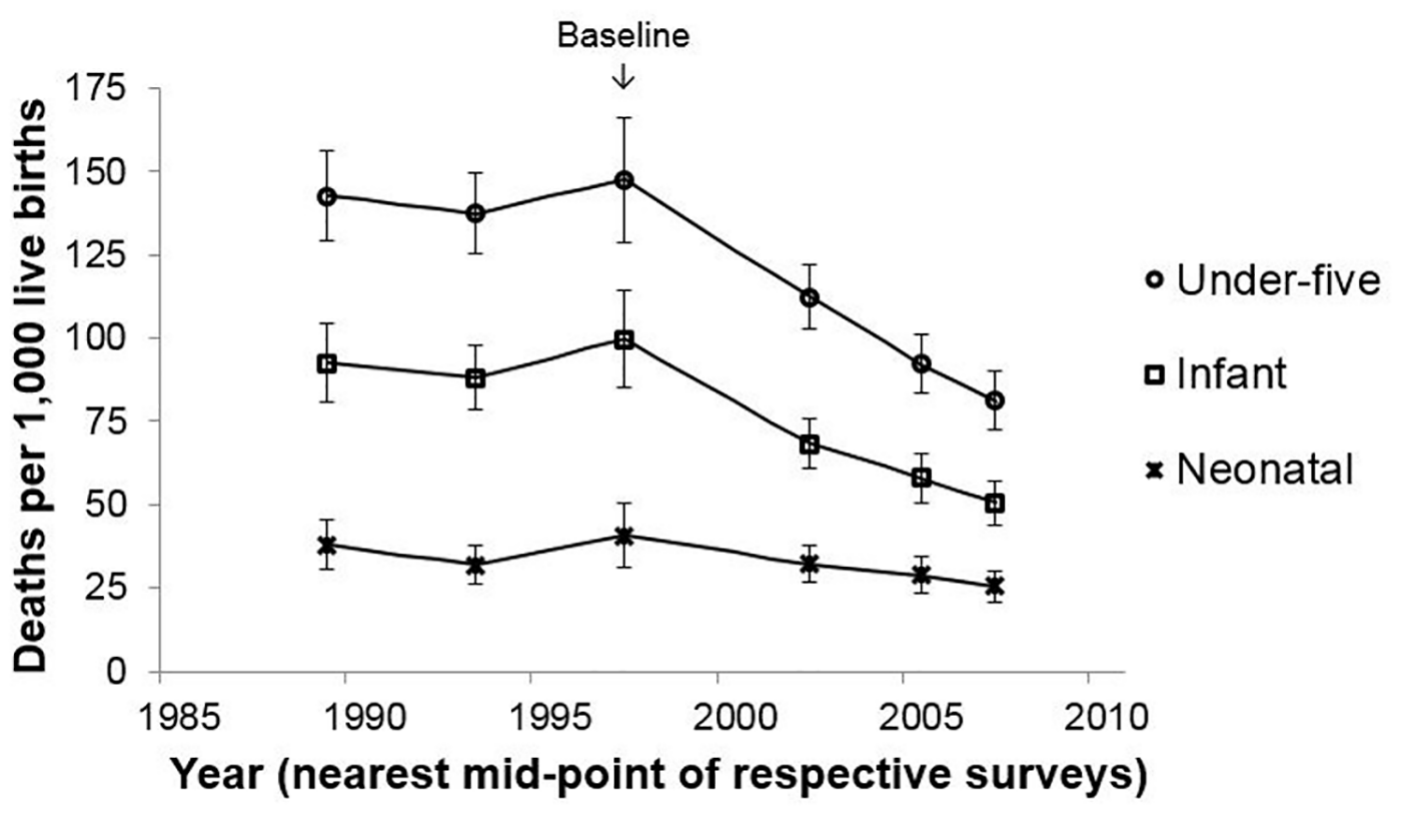
Trends in 5-year estimates of all-cause neonatal, infant, and under-five year mortality rates in Mainland Tanzania, 1990–2010. Footnote: Each rate presented as the mid-point of the five-year interval it reflects (e.g., 2010 survey, measuring mortality 2005–2009, was plotted at 2007). Mortality among children 1–59 months of age declined 54% between 1999 and 2010 (from 111 to 57 deaths per 1000 live births) compared to the 45% relative decline for the traditional 0–59 month age group described in text.

SAP among children 6–59 months of age declined 50%: from 11.1% (95% CI, 10.0–12.3%) in 2004/5, to 7.5% (95% CI, 6.6–8.6%) in 2007/8, and 5.5% (95% CI, 4.7–6.4%) in 2010 (trend *p* <0.0001). U5CM rates declined 45%: from 148 (95% CI; 129–166) deaths per 1000 live births in 1999, to 112 (95% CI, 103–122) in 2004/5, to 92 (95% CI, 83–101) in 2007/8, and 81 (95% CI, 72–90) in 2010 (trend *p* <0.0001); a decline of 67 deaths per 1,000 live births relative to baseline. Much of the decline in U5CM was due to a 49% reduction in infant mortality rate, but with no significant improvement in neonatal mortality (Fig 3): rates remained relatively unchanged across pre-baseline periods: 1987–91, 1991–5, and 1995–99.

Stratification by demographic characteristics revealed trends in ITN use among children less than 5 years of age, SAP, and U5CM associated with residence, wealth, and maternal education, but not sex. In the earlier surveys, ITN use was lower (S2A Fig) and SAP (S2B Fig) and mortality rates (S2C Fig) were higher among rural children, among children from poorer households, and among children with less educated mothers. These disparities disappeared by 2010. Small numbers in the malaria treatment categories precluded analysis of trends by residence, wealth, and maternal education.

### Plausibility assessment (1 and 2): SAP and all-cause mortality declined faster among youngest children and those residing in higher endemicity areas

**Fig 4:**
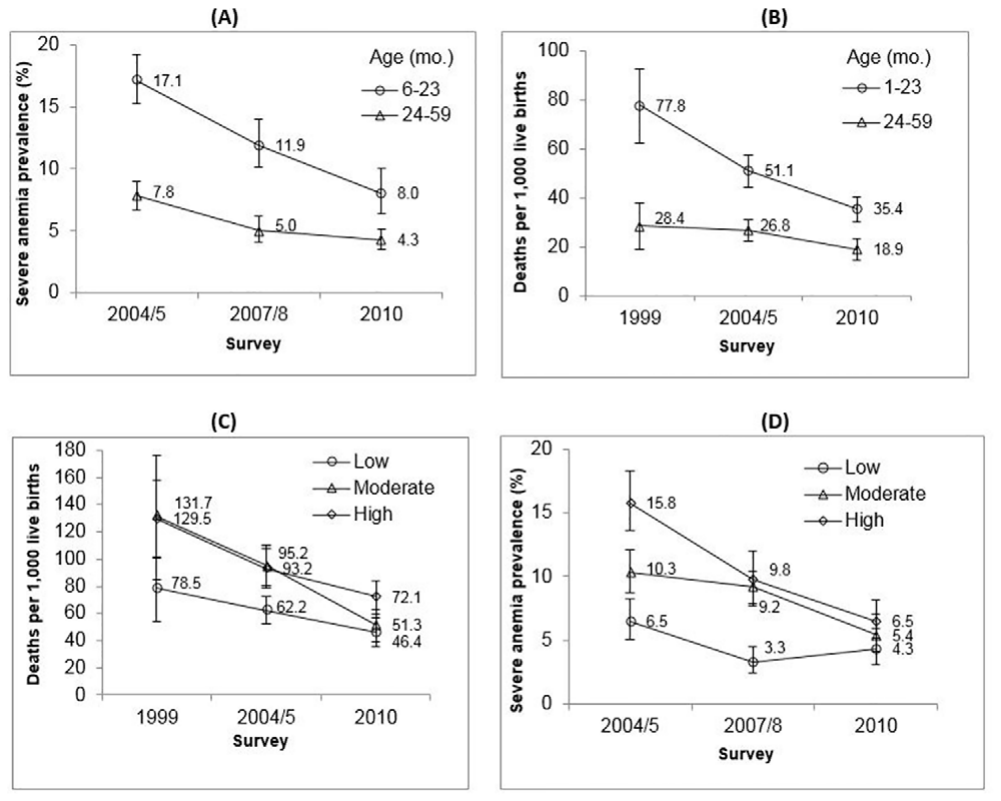
Severe anemia prevalence and all-cause mortality (deaths per 1000 live births) according to age group (A) and (B) and malaria endemicity (C) and (D), respectively.

In 2004/5, the SAP among children 6–23 months of age (17.1%) was over twice as high than in children 24–59 months of age (7.8%) (adjusted OR = 2.5, 95% CI 2.1–2.9). Subsequently, age-stratified SAP estimates improved over time, with a more accelerated decline among children 6–23 months (Fig 4A). In 2004/5, SAP was greater among children in the higher (15.8%) and moderate (10.3%) endemicity strata as compared to children in the lower (6.5%, referent) stratum (adjusted OR = 2.4, 95% CI 1.8–3.2 and OR = 1.5, 95% CI 1.1–2.0, respectively). By 2010, declines in SAP in the higher and moderate endemicity areas were at least twice as great as the declines in lower endemicity (Fig 4C). Notably, the disparity in SAP across endemcity levels was less pronounced in 2007/8, and by 2010 no differences were detected (adjusted OR = 1.4, 95% CI 0.9–2.2 and OR = 1.3, 95% CI 0.8–2.1 for higher and moderate endemicity, respectively).

Children 1–23 months of age experienced a more accelerated decline in mortality rate over time (from 78 to 35 deaths per 1000 live births) as compared to 24–59 month old children (from 28 to 19 deaths per 1000 live births) (Fig 4B).

In 1999, mortality was higher in children from higher endemicity compared to lower endemicity areas (adjusted RR = 1.4, 95% CI 1.1–1.8), but not in those from moderate endemicity areas (adjusted RR = 1.1, 95% CI 0.9–1.6). These all-cause mortality trends in children 1–59 months of age also declined markedly across endemicity (Fig 4D), with greater declines among children from the higher and moderate endemicity areas in a pattern similar to the SAP analysis. Mortality risk in 2004/5 remained greater in the higher (adjusted RR = 1.4, 95% CI 1.2–1.7) and moderate (adjusted RR = 1.4, 95% CI 1.2–1.7) endemicity groups. By 2010, mortality in the higher endemicity stratum still remained greater (RR = 1.7, 95% CI 1.3–2.1), but rates in the moderate and lower strata had converged. Overall, a more accelerated decline in all-cause mortality among 1–59 months of age occurred between 2000 and 2010 in the higher and moderate endemicity areas (reductions of 70 and 100 deaths per 1000 live births, respectively) as compared to the lower endemicity areas (reduction of 38 deaths per 1000 live births).

### Plausibility assessment (3): SAP and all-cause mortality declined even faster among younger children residing in higher endemicity

Reductions in SAP over time were more evident upon combined stratification by age and endemicity (Table 2). In 2004/5, SAP among 6–23 month old children from low, medium, and high endemicity (8.5%, 13.5%, and 20.5%, respectively) were generally twice as high as the endemicity-specific estimates (4.9%, 7.1%, and 11.0% respectively) among the 24–59 month old children. The overall reduction of SAP was most pronounced for the 6–23 month age group living in higher endemicity areas where SAP declined 67% between 2004/5 and 2010 (from 20.5% to 7.9%).

**Table 2:** Trends in severe anemia prevalence stratified by malaria endemicity* levels and age in Mainland Tanzania, 2004/5-2010.

	2004/5	2007/8	2010		
	%	%	%		
	(95% CI)	(95% CI)	(95% CI)		
	N	N	N	Change (abs.)^†^	*p* ‡
**Endemicity (6–23 mo.)**					
Lower	8.5	5.8	6.4	-2.1	0.0062
	(6.5–11.1)	(3.8–8.7)	(4.4–9.2)		
	1,099	701	596		
Moderate	13.5	14.8	9.4	-4.1	<0.0001
	(11.2–16.0)	(11.8–18.6)	(6.1–14.2)		
	944	677	754		
Higher	20.5	14.4	7.9	-12.6	<0.0001
	(17.3–24.0)	(11.3–18.1)	(5.8–10.6)		
	1,269	801	837		
**Endemicity (24–59 mo.)**					
Lower	4.9	2.0	3.2	-1.7	0.0084
	(3.53–6.83)	(1.3–3.2)	(2.1–5.0)		
	1,604	1,327	1,190		
Moderate	7.1	6.0	3.4	-3.7	0.0001
	(5.7–8.8)	(4.6–7.8)	(2.5–4.5)		
	1,289	1,171	1,512		
Higher	11.0	7.5	5.8	-5.2	<0.0001
	(9.0–13.3)	(5.6–10.0)	(4.4–7.6)		
	1,713	1,584	1,620		

* Endemicity categories: lower = 0–9.2%; moderate = 10.4–21.9%; higher = 28.7–32.6%

^†^ Percentage points absolute change, earliest value vs. 2010

^‡^ P-value obtained from chi^2^ test for linear trend in proportions across the three surveys

When mortality rates were stratified by both age and endemicity (Table 3), only the 1–23 month age group showed a significant decline across the evaluation period (trend *p* <0.0001). The largest mortality declines occurred among children aged 1–23 months living in areas of moderate and higher malaria endemicity (65.7% and 54.7% relative declines, respectively). No significant declines in mortality were detected for the 24–59 month age group within the three endemicity strata.

**Table 3:** All-cause mortality (1–59 months of age) stratified by malaria endemicity and age on Mainland Tanzania, 1999, 2004/5, 2010.

	1999	2004/5	2010		
	Rate*	Rate*	Rate*		
	(95% CI)	(95% CI)	(95% CI)	Δ (rel.)^†^	*p* ^‡^
**Endemicity (1–23 mo.)**					
Lower	49.5	35.6	29.9	-40.0	0.0001
	(30.6–68.0)	(27.3–43.9)	(21.3–38.3)		
Moderate	93.6	59.7	32.1	-65.7	<0.0001
	(47.2–137.7)	(47.5–71.6)	(22.5–41.7)		
Higher	94.9	59.4	43.0	-54.7	0.0001
	(71.7–117.6)	(46.1–72.6)	(33.4–52.6)		
**Endemicity (24–59 mo.)**					
Lower	29.1	22.9	12.9	-55.7	0.6934
	(12.8–45.1)	(16.6–29.1)	(6.2–19.5)		
Moderate	33.5	34.8	17.5	-47.8	0.6532
	(17.9–48.9)	(24.7–44.8)	(10.1–24.8)		
Higher	24.5	24.6	25.9	4.5	0.7445
	(3.5–45.0)	(17.0–32.3)	(17.4–34.4)		

* Number of deaths per 1000 live births

^†^ Percent relative change in mortality rate, 1999 value vs. 2010

^‡^ P-value obtained from chi^2^ test for linear trend in proportions across the three surveys

### Plausibility assessment (4): Changes in some contextual factors also influenced U5CM

Table 4 includes baseline and endpoint prevalence estimates (or medians where appropriate) for health-related contextual factors thought to influence U5CM.

**Table 4:** Prevalence (%) of contextual variables recognized to influence all-cause mortality over time in Mainland Tanzania, 1999 versus 2010.

	1999			2010				
	value	(95% CI)	N	value	(95% CI)	N	Δ (%)	*p*
**Socio-economic**								
Real GDP% change (1999 = 100)	**100**	-	-	**147**	-	-	47%	na
Women (15-49y) yrs of ed. (mean)	**4.7**	(4.4–5.0)	3,929	**5.7**	(5.5–5.9)	9,813	21%	**<0.0005**
Improved water source^1^	**65.8**	(59.1–71.8)	3,526	**56.9**	(53.3–60.4)	9,377	-14%	**0.016**
Households with toilet^2^	**88.8**	85.5–91.4	3,526	**86.3**	84.1–88.3	9,377	-3%	0.17
Modern floor material^3^	**20.9**	(17.0–25.4)	3,526	**31.8**	(28.7–35.0)	9,377	52%	**<0.0005**
Modern roof material^4^	**50.6**	(47.3–53.8)	9,483	**61.9**	(58.9–64.9)	9,377	22%	**<0.0005**
Problem satisfying food needs^5^	**22.6**	(21.2–24.1)	9,483	**23.3**	(21.6–25.1)	9,377	3%	0.54039
**Maternal & reproductive**							
Births any high-risk fertility category^6^	**56.9**	(53.6–60.2)	3,196	**57.1**	(55.2–58.9)	7,955	0%	0.916
ANC visits 4+	**69.9**	(63.8–75.4)	2,131	**42.7**	(40.6–44.8)	5,378	-39%	**<0.0005**
Last pregnancy protected against NNT^7^	**81.0**			**88.2**	(86.7–89.6))	5,378	9%	
Delivery at a health facility	**43.7**	(37.6–50.0)	3,196	**50.2**	(46.9–53.4)	7,955	15%	0.067
Low birth weight <2500g (%)	**8.4**	(6.3–11.2)	772	**6.1**	(5.0–7.4)	2,786	-27%	0.096
Female HIV prevalence (15-49y)^8^	**7.7**	(6.8–8.6)	5,753	**6.8**	(6.0–7.6)	7,909	-12%	na
**Nutrition**								
Median duration exclusive breastfeed (mo)	**1.1**		326	**2.4**		818	118%	
Under-fives stunted (%)^9^	**44.0**	(40.5–47.5)	2,746	**35.7**	(34.0–37.4)	7,265	-13%	**0.003**
Under-fives underweight (%)^10^	**29.5**	(26.5–32.7)	2,746	**20.5**	(19.1–22.0)	7,265	-31%	**<0.0005**
Vitamin A supplementation (6-59mo)^11^	**13.3**	(10.3–16.9)	2,503	**59.8**	(57.4–62.2)	6,638	350%	**<0.0005**
**Vaccination**								
BCG	**92.6**	(89.7–95.5)	578	**95.4**	(93.5–96.8)	1,533	3%	0.13
DPT3 / DPT3-HB-Hib	**80.9**	(73.2–86.9)	578	**87.8**	(84.8–90.3)	1,533	9%	0.064
Polio3	**79.9**	(74.4–85.3)	578	**84.9**	(81.7–87.6)	1,533	6%	0.112
Measles	**78.2**	(72.0–84.4)	578	**84.5**	(81.6–86.9)	1,533	8%	0.061
All (BCG, measles, DPT3, polio3)	**68.3**	(61.1–74.7)	578	**75.1**	(71.6–78.3)	1,533	10%	0.077
**Health Care Access**							
Health care utilisation^12^	**70.1**	63.4–76.1	1,399	**68.6**	65.9–71.1	2,776	-2%	0.669
**Morbidity (past 2 weeks)**								
Fever	**35.3**	(31.8–39.0)	2,792	**23.2**	(21.5–24.9)	7,388	-35%	**<0.0005**
Suspected ARI^13^	**13.8**	(12.0–15.8)	2,820	**7.7**	(6.9–8.6)	7,461	-44%	**<0.0005**
Diarrhoea	**12.4**	(10.6–14.5)	2,820	**14.6**	(13.4–15.8)	7,461	18%	**0.06**
Any^14^	**49.6**	(46.4–52.9)	2,820	**37.2**	(35.5–39.0)	7,461	-25%	**<0.0005**
**HIV/AIDS (UNAIDS 2011 and 2013)[109,110]**								
Adult (male/female) HIV prevalence (15-49yrs)	**7.0**	(6.3–7.8)		**5.3**	(4.8–5.8)			
Female HIV prevalence (15–49 yrs)	**7.7**	(6.8–8.6)		**6.3**	(5.7–7.0)			
Pregnant women tested for HIV	**<1%**	n/a		**86**	—			
Pregnant women HIV prevalence				**5.3**	—			
Eligible ARV coverage (all age)^15^	**<1%**	n/a		**42**	(39–46)			
Eligible children (<15 y) ARV coverage	**<1%**	n/a		**18**	(16–21)			
HIV-infected PW recv ARV for PMTCT	**<1%**	n/a		**59**	(52–68)			
HIV-exposed infants recv ARV for PMTCT	**<1%**			**68**	(59–77)			

^1^ Piped, tap, protected well, borehole

^2^ Flush, VIP or traditional pit latrine

^3^ Cement, tiles or other (not earth/sand/dung)

^4^ Iron sheets, tiles, concrete, asbestos

^5^ “often” or “always” had problems satisfying food needs in past year

^6^ Any one or combination of: parity = 1 or 4+; age <18 or 35+; birth spacing <24 months, multiple birth

^7^ NNT = neonatal tetanus. Includes mother with 2 injections during pregnancy of her last birth, or 2+ injections (past 3 years of last birth), or 3+ injections (past 5 years of last birth), or 4+ injections (past 10 year of last birth, or 5+ injections prior to last birth

^8^ Baseline figure 2003 (THIS), endpoint 2007/8 (THMIS)

^9^ Height-for-age is below -2 SD from the median of the NCHS/CDC/WHO international reference population

^10^ Weight-for-age is below -2 SD from the median of the NCHS/CDC/WHO international reference population

^11^ Percent of children aged 6–59 months who received Vitamin A supplementation in past 6 months

^12^ Percent of children 0–59 months with cough/fever/diarrhea taken to a health facility for treatment

^13^ Definition of ARI comparable across surveys

^14^ One or more of fever, ARI, diarrhea

^15^ Coverage estimates based on the estimated unrounded numbers of people receiving antiretroviral therapy and the estimated unrounded need for antiretroviral therapy (based on UNAIDS/WHO methods). The ranges in coverage estimates are based on plausibility bounds in the denominator: that is, low and high estimates of need.

#### Climate

The 12-month WASP trend over the period 1985–2010 revealed conditions were generally drier than the long-term average after 1999 (S3 Fig). The period over which baseline mortality was measured (1995–9) included a major El Niño year (1997–8). By contrast every year after 1999 was drier than the long-term average, with the exception of 2006–7. Evidence of significant warming (~ 0.3°C per decade) in many regions of Tanzania was detected.

#### Socio-economic factors

At the national level, per capita GDP was 47% higher in 2005–09 compared to 1995–9. The mean years of schooling among 15–49 year old females increased by 1.0 year between 1999 and 2010. The proportion of households with access to an improved water source declined, but the proportion with modern floor and roof materials increased. The proportion of households with a toilet or frequent problems in satisfying food needs did not change.

#### Maternal and reproductive health factors

There was no significant change in the proportion of births falling in the “high fertility risk” category, nor in the likelihood of low birth weight or facility-based delivery. The proportion of women making at least four antenatal visits during their last pregnancy declined 39% over the evaluation period, but cumulative doses of neonatal tetanus toxoid (NNT) remained high. The maternal mortality ratio in Tanzania declined 37% over the evaluation period, from 730 (95% CI, not available) to 578 (95% CI, 466–690) to 454 (95% CI, 353–556) per 100,000 live births for the 10-year period preceding the 2000, 2004/5 and 2010 surveys, respectively.

#### Nutrition

The median duration of exclusive breastfeeding rose from 1.1 to 2.4 months. Anthropometric indices (stunting and underweight) showed significant improvements of six and nine percentage points, respectively. Vitamin A supplementation for children aged 6–59 months rose more than four-fold between 1999 and 2010, from 13% to 60%.

#### Vaccination

Child vaccination coverage indicators did not change significantly between baseline and 2010. Antigens for hepatitis B and *H*. *influenzae* type B (Hib) were added to the diptheria/pertussis/tetanus (DPT) regimen in 2002 and 2009, respectively. The probability of seeking healthcare at a health facility by children aged 0–59 months who suffered cough, fever, or diarrhea did not change.

#### Other morbidity

The proportion of children with fever reported in the two weeks prior to the survey declined by one-third from 35% to 23%. Two-week prevalence of suspected acute respiratory infection (ARI: cough with rapid breathing) declined by nearly half (14% to 8%), but with no change in diarrhea prevalence (~13%).

#### HIV/AIDS

Adult HIV prevalence declined over the decade, from 7.0% to 5.3%. HIV prevalence among females (15–49) did not change between 2003 and 2007 (one percentage point decline). Prevention of mother-to-child HIV transmission (PMTCT) services commenced in 2002. National program statistics indicate that the proportion of births to HIV infected mothers protected by PMTCT rose from close to zero to approximately 50% in 2008.

### Plausibility assessment (5): Magnitude & direction of expected changes in U5CM

Malaria interventions and several other contextual factors contributed to reductions/increases in U5CM (Table 5). Among the malaria interventions scaled-up nationally, the greatest reduction in U5CM was attributable to ITN use among children less than 5 years of age, with a PAR% of 11%. Improvement in ITN use among pregnant women and IPTp (at least two doses) coverage both contributed to reductions, but only in the neonatal period (based upon published efficacy estimates for these interventions). Our derived RR for malaria-specific mortality associated with prompt access to effective first-line antimalarials yielded a PAR% of 18%.

**Table 5:** Coverage estimates of important child survival interventions and corresponding relative risk estimates with predicted proportions of all-cause under-5 mortality averted (or increased) in Mainland Tanzania, 1999–2010.

Intervention/risk factor	1999	2010	Published effect estimate	Decrease/increase in U5 mortality (PAR%)	
	(baseline)	(endpoint)	RR (95% CI)		Source^††^
**Malaria Interventions**					
ITN use <5yrs	<2%	64%	0.82 (0.75–0.90)	-11% (1–59 mo)	[49]
ITN use pregnant women	<2%	57%	0.84 (0.76–0.93)	- 9% (neonatal)	[50,51]
IPTp-2+	0%	26%	0.80 (0.71–0.91)	- 5% (neonatal)	[50]
Effective 1^st^ line therapy*	10%	27%	0.02 (0.01–0.14)	- 18% (malaria-specific, 1–59 mo)	[52]
**Other contextual factors**					
Female education (median)	4.7 y	5.7 y	0.50	- 8%	[53]
ANC visits 4+	70%	43%	0.68 (0.59–0.79)	+ 11% (neonatal)	[54]
PMTCT†	<<1%	4%	0.57	- 3%	[55]
Exclusively breastfed <6 mo.	32%	50%	0.41 (0.24–0.69)	- 8% (<6 mo)	[56]
Stunted‡	44%	36%	1.47 (1.21–1.78)	-3%	[57]
Underweight**	30%	21%	2.49 (1.56–3.97)	-9%	[57]
Vitamin A supplementation	13%	60%	0.76 (0.69–0.83)	- 10%	[58,59]

* 1999 exposure estimate assumes half of febrile children seeking treatment received ineffective therapy due to *P*. *falciparum* reduced susceptibility to chloroquine. Artemisinin-based combination therapy relative risk based on malaria-specific mortality reduction in ACT efficacy trials.

^†^ Prevention of mother-to-child transmission (PMTCT) of HIV infection. Figures represent proportion of all pregnant women (HIV infected and uninfected) receiving PMTCT

^‡^ Height-for-age is below -2 SD from the median of the NCHS/CDC/WHO international reference population

** Weight-for-age is below -2 SD from the median of the NCHS/CDC/WHO international reference population

^††^ Sources for RR estimates represent meta-analyses or multi-country studies

Among the multiple contextual factors with significant change between 1999 and 2010, at least six would be expected to result in declines in U5CM, ranging from 3% (PMTCT coverage improvement and declines in prevalence of stunting) to 10% (vitamin A supplementation improvement) based on published estimates of RR. Likewise, declines in four or more antenatal care (ANC) visits over the evaluation period were associated with mortality increases (PAR% = 11%), but mostly through neonatal deaths. These factors are not expected to be entirely independent of one another so that the individual impact magnitude cannot simply be summed. Nonetheless, this analysis serves to compare the expected impact of increased malaria intervention coverage as compared to the anticipated effect of changes in multiple other contextual factors unrelated to malaria control.

## Discussion

This impact evaluation provides plausible evidence that a 45% reduction in U5CM in Tanzania was partially attributable to dramatic reductions in SAP following increased funding and adoption of sound policies to guide the scale-up of multiple malaria interventions during the period 1999 to 2010. Younger age children (excluding neonates) from areas of higher malaria endemicity experienced the greatest declines in all-cause mortality and SAP (55% and 67%, respectively). Numerous other contextual factors associated with U5CM also improved over the evaluation period and likely contributed to declines in mortality. However, few of these factors contributed the same magnitude of mortality reduction as the combined malaria interventions. Our PAR% analysis illustrates how malaria control interventions contributed to the 45% decline in U5CM in Tanzania between 1999 and 2010, with 11% of this overall reduction attributable to ITNs alone. ITN use among Tanzanian children less than five years of age reached 64% by 2010, and further gains in child survival will be achieved with recent increases in ITN coverage [63].

After a decade (1990s) of stagnation in child survival in Tanzania, the decline in U5CM (1999–2010) was almost certainly a consequence of reductions in malaria-specific mortality associated with the 50% decline in SAP. The age and endemicity stratifications for SAP and mortality exhibited a pattern of change consistent and specific to observed malaria risk, with declines most pronounced in the more vulnerable children aged 1–23 months (6–23 months for SAP) in areas of higher malaria endemicity [42,64]. ITN coverage improved slowly between 2000 and 2007/8, and then increased abruptly by 2010. At time of the 2010 survey, children less than 24 months of age had benefited from this protection, helping to further accelerate mortality declines in this age group. While low IPTp coverage likely had only minimal impact on U5CM, these same pregnant women also benefited from access to ITN vouchers, resulting in increased protection for infants. Although no clear trend was observed in the proportion of children <5 years of age that received prompt treatment of fever with first-line antimalarials, substantial treatment efficacy had already been lost *twice* before national treatment policies transitioned. In 2000, chloroquine treatment failures by day 14 were 45% in Africa and as high as 71% in Tanzania [14] where 22 million malaria cases (confirmed or probable) were reported among children <5 years of age that year. A substantial number of these uncomplicated malaria cases treated with chloroquine likely experienced poor hematologic recovery which increased their risk of progression to severe malaria, an outcome associated with a 10–15% case-fatality rate, and up to 25–50% when accompanied by other severe sequelae [65,66]. Just five years later (2005) and only three years after SP replaced chloroquine, SP failure was 40% by day 14 [67]. Before SP was replaced with ACT as first-line treatment in 2007, some proportion of Tanzania’s annual 14–18 million uncomplicated malaria cases treated with SP during 2004–6 likely progressed to severe malaria with high case-fatality. Artemether-lumefantrine (mainland Tanzania’s first-line ACT) presently remains a highly efficacious treatment in Tanzania [68].

UNICEF estimates U5CM in Africa declined 20% between 2000 and 2010 (from 159 to 127 deaths per 1000 live births), with over one million children’s lives saved due to malaria interventions [4,69]. Recent national and sub-national analyses are more consistent with the higher reductions presented in this report. Zambia reduced U5CM 36% between 2001/2 and 2007, while severe anemia and parasitemia prevalence declined 68% and 53%, respectively, following rapid scale-up of malaria interventions [70]. U5CM in Rwanda declined 50% (from 152 to 76 deaths per 1000 live births) between 2001–5 and 2006–10, mostly following scale-up ITNs and ACTs [71]. On Bioko Island after four years of intervention scale-up (2005–08), U5CM declined 64% (from 152 to 55 deaths per 1000 live births) and SAP declined 86% (from 15% to 2%) [72]. Zanzibar, part of the Republic of Tanzania, achieved high intervention coverage earlier than the mainland and U5CM declined 52% between 2002 and 2005 [73]. A recent analysis from two districts in Tanzania detected a 43% decline in all-cause mortality among children 1–5 years of age between 1997 and 2009, with malaria interventions associated with the observed decline [74]. Our results are consistent with these published findings.

The United Nations Inter-Agency Group for Child Mortality Estimation (IGME) and the Institute for Health Metrics and Evaluation (IHME) have both derived Tanzania-specific estimates of U5CM. The decline in U5CM between 2000 and 2010 based on IGME estimates was 42% (132 to 76 deaths per 1000 live births) [69,75], quite comparable to the 45% decline in this report. However, IHME estimates correspond to a decline in U5CM of only 23% (127 to 98 deaths per 1000 live births) [76]. The IHME mortality estimates did not include 2010 Tanzania DHS data, thus the IHME modelled estimates likely over-estimated U5CM for 2010.

In 2010 the largest contributors to U5CM in Africa included perinatal causes (30%), pneumonia (14%), diarrheal diseases (11%), HIV/AIDS (4%), and malnutrition through synergy with prevalent infectious diseases (35%) [77]. With approximately 15% of U5CM in Africa caused by malaria in 2010 (down from over 20% in 2000) [78], we carefully considered over 30 other Tanzania-specific contextual factors (socioeconomic, biological, and environmental risks) in our plausibility assessment of alternative explanations for declines in U5CM. Others have shown limited or no declines in neonatal mortality rates in Africa for the period 2000–10 [76,79], and the same was observed in Tanzania. Low levels of maternal education are associated with higher rates of U5CM, and in many countries more than half of the recent reductions in child deaths are linked to improvements in female education [53,80,81]. A recent analysis showed that for each one year increase in education of reproductive-age women (as observed in Tanzania), U5CM decreased by 9.5% [53]. Likewise, a 1% annual increase in GDP per capita is associated with 0.4–0.6% reduction in U5CM [82]. Tanzania’s GDP per capita increased 47% (from $297 to $438, current U.S. dollars) between 1999 and 2010 [83], one of the fastest growing economies in Africa [84]. This growth in GDP could potentially correspond to a 18–27% reduction in U5CM, or between one-third and one-half of the 45% overall decline in U5CM over the same period. Water, sanitation and hygiene indicators did not significantly change.

The single biggest improvement among the biological determinants of child health was percentage of children 6–59 months of age who received vitamin A supplementation in the past six months. This intervention can reduce U5CM by up to 20%, particularly in high-priority countries (such as Tanzania) [58,59]. Despite a nearly 5-fold increase in vitamin A coverage between 1999 and 2010, the prevalence of vitamin A deficiency (serum retinol <0.70 μmol/l) among Tanzanian children actually increased from 24% in 1997 [85] to 38% in 2010 [86]. Thus Tanzania had a severe public health problem of vitamin A deficiency (prevalence ≥20% among child < 5 years) throughout our evaluation period. Notably, WHO recommends a vitamin A coverage threshold of 70%, the minimal level at which a country can expect to observe meaningful reductions in child mortality. Tanzania had only reached supplementation of 60% by 2010, making large reductions in U5CM attributable to this intervention unlikely [32].

Pregnant women, their unborn fetuses, and newborns benefit from four or more antenatal care (ANC4+) visits [54]. Two doses of tetanus toxoid to protect against neonatal tetanus is one intervention provided at ANC [87]. During the evaluation period, pregnant women completing ANC4+ visits declined by 39% which may have increased neonatal mortality. However, sufficient cumulative dosage of tetanus toxoid remained high and thus did not account for accelerated declines in U5CM. Maternal survival has a dramatic impact on U5CM, but it is very difficult to measure and confidence intervals are often wide [88,89]. The 37% decline in the maternal mortality ratio between 2000 and 2010 (from 730 to 460) [90] likely accelerated the declines in U5CM through other biologic determinants we examined.

Malaria transmission is sensitive to temperature and rainfall, but the effect of these on malaria morbidity and mortality are not well delineated [91]. Temperature influences the extrinsic life-cycle of the parasite and may also modify vector or human behavior, all combining to affect the transmission dynamics of sporozoites [92]. While rainfall suitable for malaria transmission persisted in Tanzania throughout the evaluation period, it is unlikely that variations in rainfall significantly altered malaria transmission. Climatic variables alone did not predict malaria outcomes in other analyses from northeastern Tanzania [93,94].

Tanzania experienced changes in multiple indicators of childhood nutrition, an important determinant of U5CM. Exclusive breast feeding in the first six months of life is recommended by WHO and UNICEF to partially protect against diarrheal and acute respiratory illness and mortality. Supplementation of breast milk with other liquids and foods begins very early in Tanzania: nearly half of infants younger than 2–3 months receive supplements. Given the modest protective efficacy of exclusive breastfeeding versus partial or predominant breastfeeding [56,95], and the limited change in prevalence of exclusive breastfeeding in Tanzania over time, this practice likely accounted for only a small decline in U5CM. Stunting and underweight prevalence reflect shortness-for-age and low weight-for-age, respectively, and are indicators of chronic malnutrition. Models estimate that a 5% reduction in prevalence of underweight is associated with a 13% reduction in U5CM [96]. Wasting prevalence (low weight-for-height) can reflect a recent and severe event causing substantial weight loss, but this indicator did not change in Tanzania during the evaluation period. No significant improvement in immunization coverage was detected during the evaluation period with the exception of *Haemophilus influenza* type b (Hib), introduced in 2009. While Hib is a contributor of meningitis and pneumonia deaths among Tanzanian children under five, the rapid Hib vaccine coverage achieved by 2010 (88%) is unlikely to have caused a decline in U5CM so soon after introduction (less than one year).

Among the 49.7 million HIV infections that occurred worldwide by 1999, 72% were in sub-Saharan Africa; as were 91% of child HIV infections and 94% of child AIDS deaths [97]. HIV prevalence in Tanzania was monitored through household surveys and ANC sentinel sites over our evaluation period. While HIV prevalence among adults declined over 30% between 1999 and 2010, prevalence did not significantly decline among women of reproductive age or pregnant women. Estimates of pediatric HIV/AIDS prevalence in Tanzania are not available, but in the absence of antiretroviral therapy, over 50% of HIV-infected infants progress to AIDS and die within 24 months [98]. As part of the global effort to eliminate new HIV infections in infants, Tanzania accomplished major improvements in the provision of preventive services and care and treatment for HIV/AIDS, particularly PMTCT coverage which reached the majority of HIV-exposed infants by 2010. While PMTCT scale-up has reduced U5CM in Africa [99], the scale-up of in Tanzania mostly occurred after 2007 and benefited a relatively small proportion of the population less than five years of age. The best estimate of the percentage of pediatric deaths in Tanzania due to HIV/AIDS during 2010 was 5%, down from 8% in 2000 [77].

Our approach was accompanied by multiple limitations. We were unable to include national-level malaria morbidity/mortality data from health facilities, resulting in analyses entirely dependent upon household survey data. Using parasitemia prevalence data from only the 2007/8 Malaria Indicator Survey likely resulted in misclassification of our malaria endemicity categories over time. However, since malaria intervention scale-up in Tanzania occurred at a national level, this exposure misclassification over time was likely non-differential and thus not disproportionately higher among children with anemia or those who died at a younger age. More recent reports from two distinct locations in Tanzania [100,101], while limited to only one or two villages, indicate major reductions in parasitemia over our same evaluation period. Health facility-based data would provide longitudinal and spatial changes in parasitema and anemia, important outcomes in the causal pathway considered in this evaluation. While all-cause mortality data from household surveys have excellent representativeness [22], the principle limitation is the bias introduced to mortality estimates based solely upon birth histories reported by respondents [102]. A disadvantage of using U5CM is the lack of specificity for malaria deaths and the non-malarial causes must be accounted for through adequacy and plausibility assessments. Likewise, U5CM reflects no impact for older children and adults who also benefit from malaria control in Tanzania, particularly those living with HIV/AIDS [103]. Monitoring ITN, IPTp, and case management outcome indicators through household surveys has multiple limitations. Prior to the deployment of LLINs (with insecticide incorporated into netting material), insecticide was hopefully applied directly to bednets by the beneficiary. Inability of a survey respondent to correctly recall timing of net retreatments may have produced unreliable estimates of ITN coverage. An appropriately treated ITN or an LLIN with sufficient insecticidal content may still not protect a user due to excessive holes/tears in the net. The proportion of children less than five years of age who sleep under an ITN continues to be influenced by perceptions regarding seasonal variation in malaria transmission risk [104,105]. Since these surveys are often done during the dry season to facilitate field team mobility, ITN use for the rest of the year may be underestimated [106]. IPTp coverage may be unreliable stemming from the respondent’s inability to identify which drug was given or how many doses [107]. A final major limitation of these household surveys is the lack of contemporary exposure data for children who died within the period covered by the survey. ITN use (night before the survey), first-line treatment (past two weeks), and all contextual factors were assessed only for children who survived through the time of the survey visit, but are not assessed for child deaths in the whole 5-year period preceding the survey. This limits the conventional epidemiologic analyses of individual-level exposure/outcome data and increased reliance upon a plausibility design [24].

Malaria case management (testing and treatment) indicators from household surveys are particularly problematic for examining trends over time [52]. Respondents’ recall of facility-level diagnostic testing and treatment may not be valid. A final limitation relates to Hb measurement and estimates of SAP. Hb concentration among African children is influenced by many determinants including age, sex, nutrition, infections, environment, and genetic factors [108]. While malaria interventions have been associated with a 60% reduction in the risk of moderate-to-severe anemia (Hb < 8 g/dL) [70], in most of Africa the leading cause of pediatric anemia is iron deficiency. Aside from age and malaria endemicity, our analyses did not examine other anemia causing exposures. Notably, among Tanzania children 6–59 months of age only 1.4% received an iron supplement within seven days preceding the 2010 DHS [32].

Continued progress in malaria intervention scale-up and impact on the malaria burden in Tanzania are further supported by the most recent 2012 Tanzania HIV/AIDS and Malaria Indicator Survey [39]. The national malaria prevalence estimate using RDTs declined to 10% in 2012, a 47% relative reduction from the national prevalence of 18% in 2007/8 (also RDT-based). One high burden region (Kagera) targeted for intensive malaria control efforts since 2007 experienced a decline in malaria prevalence from 41% in 2007/8 to 8% in 2012 (and SAP among children 6–59 months declined form 9.3% to 3.2%). Nationally, household ownership of at least one ITN was 91% in 2012, up from less than 40% in 2007/8. The 2012 survey did not include new estimates for U5CM, but SAP among children 6–59 and 6–23 months (5.6% and 7.8%, respectively) were not significantly different from 2010 estimates.

## Conclusion

After no improvement in child survival during the 1990s, the 45% reduction in U5CM and 50% reduction in SAP following malaria intervention scale-up is a major public health achievement for Tanzania. This impact was more accelerated among younger children (<23 months of age) living in higher malaria endemicity areas, the very children with the greatest potential to gain health improvements following malaria intervention scale-up. While other factors also contributed to the observed declines in U5CM, the large proportion of decline plausibly attributable to malaria control efforts is noteworthy. The progress achieved in malaria control can reverse quickly in the absence of continued financial support and strong political will. To avoid a return to the intense perennial transmission conditions that prevailed for so long, sustained focus on and support for comprehensive programs to control malaria will be required in Tanzania and other African countries.

## Supporting Information

S1 Appendix International Research Institute for Climate and Society/Mailman School of Public Health, The Earth Institute, Columbia University. Development of climate analysis section for the President’s Malaria Initiative impact evaluation: reports for Ethiopia and Tanzania, August 2012.
(PDF)

S1 Fig Annual funding levels and source of malaria control effort (A) and number of commodities procured and distributed (B) in Mainland Tanzania, 2000–10. Footnote: *GoT = Government of Tanzania; PMI = U.S. President’s Malaria Initiative
(TIF)

S2 Fig Insecticide treated net (ITN) coverage (A), severe anemia (Hb< 8.0 g/dL) prevalence (B), and all-cause mortality among children under-five (C) stratified by background characteristics in Mainland Tanzania, 2004/5, 2007/8, and 2010. (TIF)

S3 Fig Weighted anomaly of standardized precipitation (WASP) analysis for Tanzania comparing (A) baseline period (1995–99) and (B) malaria intervention scale-up years (2000–10). Footnote: red indicates drier, green indicates wetter (compared to baseline)
(TIF)
