# Supplementary material for: Impact of Malaria Control on Mortality and Anemia among Tanzanian Children Less than Five Years of Age, 1999–2010
Source: PLoS One. 2015 Nov 4;10(11):e0141112. doi: 10.1371/journal.pone.0141112 (PMC4633136; doi:10.1371/journal.pone.0141112)
Supplement: S1 Appendix — Development of climate analysis section for the President’s Malaria Initiative impact evaluation: reports for Ethiopia and Tanzania, August 2012. (PDF) [file pone.0141112.s001.pdf]

President's Malaria Initiative-USAID Report

Development of Climate Analysis Section for the President's Malaria Initiative Impact Evaluation: Reports for Ethiopia and Tanzania.

Submitted: July 30, 2012
Revised: August 30 2012

Submitted to:
Rene Salgado
PMI-USAID

Submitted by:
Madeleine C. Thomson
Senior Research Scientist, International Research Institute for Climate and Society/Mailman School of Public Health
The Earth Institute, Columbia University

A PAHO-WHO Collaborating Center for Early Warning Systems for Malaria and other climate sensitive diseases

CONTENTS

1 EXECUTIVE SUMMARY 1-5

1.1 FINANCIAL SUPPORT 1-9
1.2 AUTHORS OF THE REPORT 1-9
1.3 CONTRIBUTORS TO THIS REPORT AND ITS APPENDICES 1-9
1.4 ACRONYMS 1-11

2 INTRODUCTION 2-13

2.1 THE PRESIDENT'S MALARIA INITIATIVE 2-13
2.2 AIM AND OBJECTIVES OF THIS STUDY 2-15

3 REVIEW OF CLIMATE AND MALARIA IN AFRICA 3-16

3.1 WHY IS CLIMATE UNIQUE? 3-16
3.2 CLIMATE – MALARIA INTERACTIONS 3-16
3.3 RAINFALL 3-17
3.3.1 RAINFALL IN SEMI-ARID REGIONS 3-17
3.3.2 RAINFALL IN HIGHLAND REGIONS 3-18
3.3.3 DROUGHTS 3-18
3.4 TEMPERATURE 3-19
3.4.1 THE RELATIONSHIP BETWEEN TEMPERATURE AND ALTITUDE 3-19
3.4.2 TEMPERATURE AND MALARIA PARASITE DEVELOPMENT WITHIN MOSQUITO 3-19
3.4.3 TEMPERATURE AND MOSQUITO DEVELOPMENT 3-20
3.5 THE RELATIONSHIP OF MALARIA, LOCAL CLIMATE TO GLOBAL CLIMATIC PROCESSES 3-21
3.6 THE CLIMATE OF EASTERN AFRICA 3-22
3.6.1 EVIDENCE OF RECENT WARMING IN EASTERN AFRICA 3-22
3.6.2 EVIDENCE OF RECENT DRYING IN EASTERN AFRICA 3-25
3.6.3 POSSIBLE CAUSES OF THE EASTERN AFRICA DROUGHT AND WHY THEY MATTER 3-26
3.7 IN SUMMARY 3-27

4 METHODOLOGY 4-28

4.1 METHOD 1: CLIMATE INFORMATION ANALYSIS (CIA) METHODOLOGY 4-28
4.2 METHOD 2: CLIMATE INFORMATION, MALARIA AND INTERVENTION ANALYSIS (CIMIA) METHODOLOGY 4-29

5 DATA SETS 5-30

5.1 MALARIA DATA 5-30
5.1.1 THE DHS (USAID) SURVEYS 5-30
5.1.2 ZONE LEVEL IDSR DATA FROM ENHRI (2005-2009). 5-31
5.1.3 NATIONAL MALARIA INCIDENCE DATA FROM ENHRI (1983-2010). 5-31
5.1.4 NUMEROUS VARIED LOCAL TIME SERIES OF MALARIA INCIDENCE DATA. 5-31
5.1.5 INTERVENTION DATA FROM PMI PARTNERS IN ETHIOPIA. 5-31
5.2 INTERVENTION DATA 5-31
5.3 CLIMATE DATA 5-34
5.3.1 METEOROLOGICAL OBSERVATIONS 5-34
5.3.2 GLOBAL PRODUCTS 5-34

5.3.3 CLIMATE CHANGE SCENARIOS 5-34
5.3.4 COMBINING GROUND OBSERVATIONS AND GLOBAL PRODUCTS 5-35
5.3.5 ENHANCED NATIONAL CLIMATE SERVICES (ENACTS) 5-35

6 NEW TOOLS CREATED 6-37

6.1 CLIMATE SUITABILITY FOR MALARIA TRANSMISSION TOOL 6-37
6.2 WASP TOOL 6-38
6.2.1 BASELINES 6-38
6.2.2 WEIGHTED ANOMALY OF STANDARDIZED PRECIPITATION (WASP) WITH CHANGEABLE BASELINE FOR IMPACT ASSESSMENT 6-39
6.3 CLIMATE ANALYSIS TOOL 6-40

7 THE USE OF CLIMATE INFORMATION IN THE ASSESSMENT OF THE IMPACT OF MALARIA INTERVENTIONS: ETHIOPIA 7-41

7.1 MALARIA IS CLIMATE SENSITIVE IN ETHIOPIA 7-41
7.2 ENACTS CLIMATE PRODUCTS FOR ETHIOPIA 7-43
7.3 BASELINES 7-44
7.4 FINDINGS FROM ANALYSIS 7-45
7.5 IN SUMMARY 7-47

8 THE USE OF CLIMATE INFORMATION IN THE ASSESSMENT OF THE IMPACT OF MALARIA INTERVENTIONS: TANZANIA 8-48

8.1 MALARIA IS CLIMATE SENSITIVE IN TANZANIA 8-48
8.2 CLIMATE DATA AND PRODUCTS FOR TANZANIA 8-50
8.3 THE CLIMATE SUITABILITY FOR MALARIA TRANSMISSION TOOL (CSMT) 8-50
8.4 BASELINES 8-51
8.5 FINDINGS FROM ANALYSIS 8-53
8.6 IN SUMMARY 8-53

9 CONCLUSION 9-55

9.1 RECOMMENDATIONS 9-55

10 REFERENCES 10-57

11 APPENDIX I: WORKSHOP REPORT “THE USE OF CLIMATE INFORMATION IN IMPACT ASSESSMENT FOR MALARIA INTERVENTIONS” ADDIS ABABA, ETHIOPIA 11-61

12 APPENDIX II: WORKSHOP REPORT “DATA QUALITY CONTROL, SATELLITE RAINFALL ESTIMATION, AND MERGING STATION OBSERVATIONS WITH SATELLITE ESTIMATES. DAR ES SALAAM, TANZANIA. 12-62

Figures

Figure 3-2 Malaria cases and rainfall in Ovamboland, Namibia 3-18
Figure 3-3 Temperature and the extrinsic incubation period [35] 3-20
Figure 3-4 Malaria incidence, rainfall (CMAP) and sea surface temperature in Botswana 3-21
Figure 3-5 Malaria cases and Minimum Temperature at Kericho, Kenya, compared to Global SSTs, Tropical LST and ENSO 3-24
Figure 3-6 Differential impact of rainfall and ENSO on maximum (Tx) and minimum (Tn) temperature 3-25
Figure 3-8 Time series of March-May rainfall anomalies relative to a 1979-2010 baseline averaged across East Africa land areas 3-26
Figure 5-5-1 Intervention Score distribution in Oromia 5-33
Figure 6-6-1 The Pan African Climate Suitability for Malaria Transmission Tool 6-38
Figure 6-6-2 Malaria morbidity in Eritrea 1996-2003 6-39
Figure 6-6-3 Weighted Anomaly of Standardized Precipitation for Eritrea using a 1999 (wet) baseline 6-39
Figure 6-6-4 Weighted Average Standardized Anomaly Rainfall for Eritrea using a 2000 (dry) baseline 6-40
Figure 7-1: Time series of LSTs and SSTs compared to Minimum Temperature from Kericho, Kenya (1979-2009)[48] 7-42
Figure 7-4: CSMT for Ethiopia using ENACTS products indicating highly seasonal transmission in green areas such as Tigray, a long single transmission season in the warm lowlands of Gambela and a bio-modal seasonality in the highly varied Oromia region 7-44
Figure 7-6abc Trend in rainfall (top) max T (middle) and min T (bottom) for the Belg rainy season (Feb-May). 45
Figure 7-7abc Trend in rainfall (top) max T(middle) and min T (bottom) for the Kiremt rainy season (Jun-Sep). 45
Figure 7-8 Regional WASP analysis for Ethiopia using ENACTS products using 2000-2005 baseline .. 7-45
Figure 7-9 Zonal WASP analysis for Oromia, Ethiopia using ENACTS products with a 2000-2005 baseline 7-46
Figure 8-1 Time series of LSTs and SSTs compared to Minimum Temperature from Kericho, Kenya (1979-2009)[48] 8-49
Figure 8-4 CSMT P. falciparum for Rungwe District, Mbeya, Southern Tanzania 8-51

Tables

Table 3-1 Temporal correlation between climate variables (maximum temperature, minimum temperature and sea surface temperatures) 3-24
Table 5-1 Finding contemporaneous data 5-31
Table 5-2 Interventions and associated partners 5-32
Table 5-3 Dependent and independent intervention variables 5-32
Table 4 Possible outcomes if climate information is not incorporated into malaria impact assessment: 9-55

Technical Report

1 Executive Summary

The Monitoring and Evaluation Team of the President's Malaria Initiative identified the need for technical expertise in climate analysis to ensure that their impact evaluations benefit from the most up-to-date methods and are consistent across countries. This report describes the results obtained from a study, partially funded by PMI (July 2011-July 2012), to develop a standardized methodology for climate analysis in relation to malaria epidemiology for PMI's (along with national government and RBM partner) Impact Evaluation. In addition, the project aimed to produce climate analysis reports for two PMI impact evaluation countries: Ethiopia and Tanzania.

1. **Review of the literature and data sources** (Section 3)
    a. Climate and malaria interactions (including those involving sea surface temperatures) from studies published and grey literature were briefly reviewed in the context of the evaluation of the impact of climate as a potential confounder in the assessment of malaria interventions. This included a brief review of:
        i. The warming of Eastern Africa (Section 3.6.1)
        ii. The drying of Eastern Africa (Section 3.6.2)

2. **Methodology** (Section 4). Two linked methodological approaches for the incorporation of climate information into malaria intervention impact assessment were investigated. These two methodologies are:

    a. **Climate Information Analysis (CIA)**

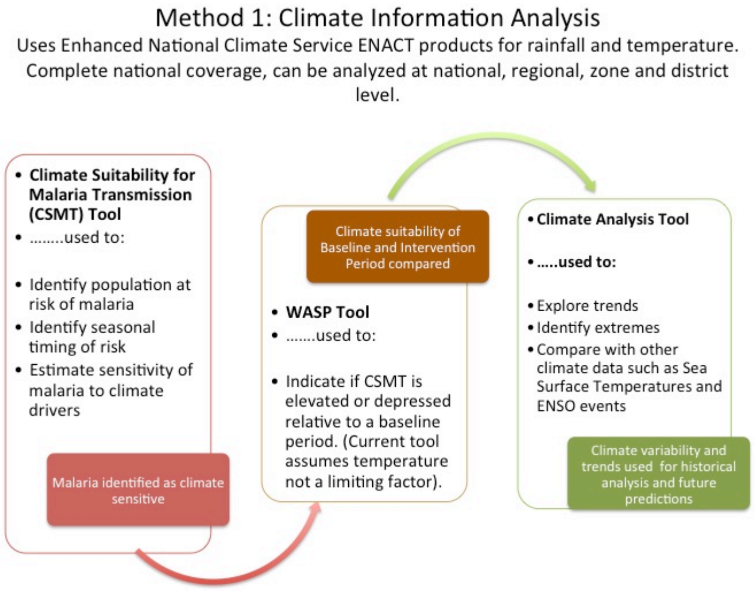

where in the absence of malaria and intervention data only climatic factors are investigated. This approach was applied to: i.) Ethiopia Section 7 where limited malaria data were available and ii) Tanzania Section 8 where no malaria and intervention data were available to this study.

Possible outcomes if climate information is not incorporated into malaria impact assessment:

	**Malaria decreases following intervention**	**Malaria remains the same or increases following intervention**
**Climate suitability for malaria transmission increases following intervention**	Failure to incorporate climate in analysis may underestimate benefits of intervention	Failure to incorporate climate in analysis may result in resurgence being blamed inappropriately on non-climatic factors or conversely climate being blamed for resurgence when in fact control failure is responsible
**Climate suitability for malaria transmission does not change following intervention**	No further climate analysis required	No further climate analysis required
**Climate suitability for malaria transmission decreases following intervention**	Failure to incorporate climate in analysis may overestimate benefits of intervention	Failure to incorporate climate may underestimate the importance of non-climatic factors in driving malaria increase.

b. **Climate Information, Malaria and Intervention Analysis (CIMIA)**

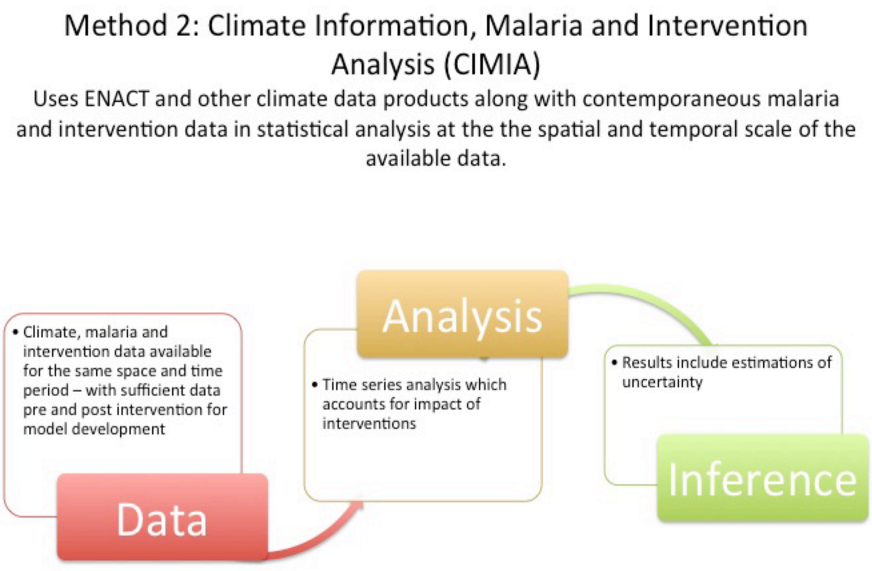

This approach was explored with limited data on malaria and intervention from Ethiopia. The statistical analysis was performed by Peter Diggle's group at Lancaster/Liverpool University (see document "Malaria,

Incidences, and Climate Analysis: Ethiopia" for more information, username: PMI, password: pietro). However, due to the limited number of years for malaria and intervention data, it was impossible to statistically identify the contribution of intervention and climate factors on the trends of malaria cases. In order to implement this approach, this would require longer time-series of cotemporaneous data on all three dimensions – malaria incidence, climate and intervention – observed over a time-period that spans the introduction of the interventions in question.

3. **Data sets** (Section 5)
    a. Climate, Malaria and Intervention data were identified and explored. challenges in data quality and availability were identified.
    b. Appropriate data set were accessed from partners and new databases created as needed.
        i. Malaria data (Section 5.1) from disparate sources were accessed for Ethiopia including:
            1. Zone level IDSR data from the Ethiopia Health and Nutrition Research Institute (EHNRI) (2005-2009)
            2. National HMIS malaria incidence data from ENHRI (1983-2010)
            3. Numerous local time series of malaria incidence data were explored during the impact workshop
        ii. Intervention data (Section 5.2) from PMI partners were accessed for Ethiopia's Oromia region, including:
            1. Quarterly reports on commodity distribution and geographic coverage (2008-2010).
        iii. Climate data (Section 5.3) obtained from Enhanced National Climate Services (ENACTS) products, including rainfall and temperature. These were developed for Ethiopia and Tanzania with the support of Reading University, the World Meteorological Organization and the National Meteorological Agencies of Ethiopia (NMA) and Tanzania (TMA). They included:
            1. Quality assured rainfall and temperature time series generated for each 10km grid of the country from merged observation and satellite data going back 30 years
            2. IRI Data Library data management, analysis and visualization tool installed in each country
            3. Data products disseminated via National Meteorological Agency websites

4. **New Tools Created (Section 6)**
    a. New tools were developed for climate – malaria analysis that could use the ENACTS climate databases created for Ethiopia and Tanzania. These were:
        i. **Climate Suitability for Malaria Transmission (CSMT) Tool** designed to help identify regions (including administrative boundaries) where climate variability and trends are likely to be significant. CSMT results for Ethiopia (*P. falciparum* and *P. vivax*) and Tanzania (*P. falciparum* and *P.vivax*) are available at:

Ethiopia Falciparum, Vivax; Tanzania Falciparum, Vivax (click on the links and use username: PMI; password: pietro).

ii. **Weighted Anomaly of Standardized Precipitation (WASP) Tool** designed to establish whether or not the intervention period was substantially wetter or drier than the baseline period.

iii. **Climate Analysis Tool** designed to explore trends in the rainfall and temperature data by year, season or month. Climate variability and its relationship with ENSO can also be explored which will indicate the likelihood that the region will experience major anomalies during an ENSO event.

5. **Short Reports** were created using the CIA methodology for USAID and partner staff to summarize information on the relevant climate data for malaria impact assessments for two PMI countries.
    a. "The use of climate in the assessment of the impact of malaria interventions: Ethiopia" (Section 7)
    b. "The use of climate in the assessment of the impact of malaria interventions: Tanzania" (Section 8)

6. **Appendix: Training workshops undertaken (Section 11 & 12)**
    a. A training workshop was held in Addis Ababa, Ethiopia, during December, 12-14, 2011, facilitated by the Anti-Malaria Association/Climate and Health Working Group of Ethiopia and the International Research Institute for Climate and Society/Earth Institute, Columbia University, New York, USA and in collaboration with the Federal Ministry of Health, the National Meteorological Services Agency of Ethiopia. The workshop, entitled "The Use of Climate Information in Impact Assessment for Malaria Interventions" focused on the training of national malaria experts (both practitioners and researchers) in the use of the new ENACTS databases in malaria impact assessments. Representatives from the National Meteorological Agency also participated. A report on the meeting was produced (Appendix 1 Section 11)

    b. A training workshop was held in Dar es Salaam in collaboration with the Tanzanian National Meteorological Agency during June 25 July, 6, 2012. The workshop, entitled "Data Quality Control, Satellite Rainfall Estimation, and Merging Station Observations with Satellite Estimate" focused on the training of national meteorological agency staff in the creation of the ENACTS climate database for Tanzania. A report on the meeting was produced (Appendix 2 Section 12).

1.1 Financial Support

Funding for this project was provided by the President’s Malaria Initiative, USAID. The project was administered through an IRI/EGAT-USAID Cooperative Agreement “Weather and Climate Information for Climate Resilient Development (CCRD)” PI. A. Robertson. A subcontract was awarded to an IRI partner, the Health and Climate Foundation (a US-based 5013c; http://hcfoundation.org), to facilitate a short-term consultancy as well as the disbursement of funds for the workshop held in Tanzania.

The project leveraged resources from additional partners and projects namely: USAID-EGAT CCRD award to IRG, and a NOAA Institutional grant NA07GP0213.

In particular this study leveraged a three year Google.org funded project ”Building Capacity to produce and use climate information for improving health in East Africa”. In particular the current analysis incorporates the key output from the Google.org project namely the new climate database for Ethiopia (Enhanced National ClimaTe Services; ENACTS product) developed by the National Meteorological Agency of Ethiopia with support from IRI. Funding from the Google.org project was used to support the workshop “Use of Climate Information in Impact Assessment for Malaria Interventions” at UNECA in Addis Ababa, Ethiopia from December 12-14, 2011 which was convened by the Anti-malaria Association/Climate and Health Working Group of Ethiopia, and IRI.

The Health and Climate Foundation was a subcontractor for this project and helped facilitate the workshops and support key consultancies.

The World Meteorological Organization also provided financial and facilitation support to the Tanzania workshop which was used to develop the ENACTS products for Tanzania.

1.2 Authors of the Report

Madeleine Thomson, IRI/MSPH
Frank Zadravecz, MSPH
Brad Lyon, IRI
Gilma Mantilla, IRI
Derek Willis, IRI/CRED
Pietro Ceccato, IRI
Tufa Dinku, IRI

1.3 Contributors to this Report and its Appendices

Pietro Ceccato, IRI
Remi Cousin, IRI
Steve Connor, Liverpool University
John del Corral, IRI
Peter Diggle, Lancaster/Liverpool University
Tufa Dinku, IRI Review
Patricia Graves, James Cook University
Augustine Kanemba, TMA
Agnes Kijazi, TMA

Nassib Ibrahim, TMA
Brad Lyon, IRI
Gilma Mantilla, IRI
Emmanuel Mpeta, TMA
Judy Omumbo, Independent Consultant
Barbara Platzer, CGC
David Rogers, (HCF)
Ben Taylor, Lancaster University
Madeleine Thomson, IRI/MSPH
Wilbert Timiza, TMA
Derek Willis, IRI
Frank Zadravecz, MSPH

1.4 Acronyms

ALR	Adiabatic Lapse Rate
ACCM	All-cause child mortality
ACTs	Artemisinin-based Combination Therapies
AMA	Anti-Malaria Association
CAMS_OPI	Climate Anomaly Monitoring System and OLR Precipitation Index
CCD	Cold Cloud Duration
CDC	Center for Disease Control
CGC	Columbia Global Center
CHWG	Climate and Health Working Group
CSMT	Climate Suitability Malaria Transmission
CMAP	Climate Prediction Center Merged Analysis of Precipitation
CRED	Center for Research in Environmental Decisions
DJF	December January February
EGAT	Economic Growth and Trade
EHNRI	Ethiopian Health and Nutrition Research Institute
ENACTS	Enhanced National Climatology time Series
ENSO	El Niño Southern Oscillation
FMoH	Federal Ministry of Health
GCMs	Global Climate Models
GFATM	Global Fund for Aids Tuberculosis and Malaria
GMAP	Global Malaria Action Plan
GIS	Geographical Information Systems
GPCC	The Global Precipitation Climatology Centre
HCF	Health and Climate Foundation
HMIS	Health Management Information System
HIMAL	Highland Malaria Project
IDSR	Integrated Diseases Surveillance and Response System
IPCC	Intergovernmental Panel on Climate Change
IPT	Intermittent Preventive Treatment
IPTp	Intermittent Preventive Treatment of malaria in pregnancy
IRI	International Research Institute for Climate and Society
IRG	International Resources Group
IRS	Indoor residual spraying
ITNs	Insecticide-treated nets
MARA	Mapping Malaria Risk in Africa
MDGs	Millennium Development Goals
MERG	Monitoring and Evaluation Reference Group
MSPH	Mailman School of Public Health
NASA	National Aeronautics and Space Administration
NMA	National Meteorological Agency
NOAA	National Oceanic and Atmospheric Administration, USA
PHEM	Preventive Health Emergency Management
PMI	President Malaria Initiative
QC	Quality Control
RBM	Roll Back Malaria
SST	Sea Surface Temperature
TMA	Tanzanian Meteorological Agency
UNECA	United Nation Economic Commission for Africa

UNICEF	United Nations Children's Fund
USAID	United States Agency for International Development
WASP	Weighted Anomaly of Standardized Precipitation
WHO	World Health Organization

2 Introduction

Malaria is the most important parasitic disease worldwide with the highest burden borne by endemic countries in sub-Saharan Africa where it accounts for an estimated 0.5 - 2 million deaths each year, mostly in children under the age of 5 years [1]. Over the last decade, control and, more recently elimination, of malaria has been prioritized by governments of endemic countries and the focus of intensified international donor support [2]. Donor support is increasingly contingent on evidence of impact. For example, the malaria targets for the reduction in malaria morbidity and mortality established for the Millennium Development Goals and the Roll Back Malaria partnership require measurement of specific malaria outcome indicators in order to evaluate the effectiveness of interventions toward their achievement.

Malaria is a complex disease: its transmission, via vector mosquitoes (*Anopheles* sp.) can be highly climate sensitive with temperature being a significant driver of the development rates of both mosquito vector and plasmodium parasite while rainfall and humidity provide essential environmental characteristics for juvenile mosquito development and adult survivorship respectively. Climate has been identified as one of a number of possible confounders in the evaluation of malaria interventions. Climate information, based on routinely collected data, obtained via globally recognized standards at defined regular time intervals, can be systematically incorporated into malaria analyses at multiple spatial and temporal scales. If climate is not taken into account, then the measurement of achievements may be overly pessimistic in years that experience an elevated climate risk for malaria in relation to the baseline period or conversely overly optimistic when climate risk from malaria is low.

2.1 The President's Malaria Initiative

The US President's Malaria Initiative (PMI) has supported malaria control in 19 high malaria burden countries in sub-Saharan Africa since its launch in 2006. In support of the Global Malaria Action Plan (GMAP) goal of achieving a 50% reduction in malaria-related mortality, PMI aims to achieve 85% coverage of vulnerable populations with 4 proven interventions (insecticide-treated nets (ITNs), indoor residual spraying (IRS), intermittent preventive treatment of malaria in pregnancy (IPTp) and artemisinin-based combination therapies (ACTs). Since 2005, the scaling-up of coverage with these proven interventions has progressed in many African countries with the expectation that as coverage increases a significant reduction in the malaria burden will occur. The World Malaria Report 2010 highlights that continued progress has been made towards meeting international targets for malaria control to be achieved by 2010 and 2015. The report also provides evidence of the scale-up of the four key interventions, with a dramatic increase in provision of interventions across the board in 2008.

To date, several countries in Africa have reported that they have achieved target levels for intervention coverage [2]. Furthermore, there are an increasing number of reports describing declining incidence of malaria hospitalization, deaths and prevalence [3, 4] and a strong case has been made that the implementation of malaria interventions at

scale has produced a demonstrable impact in a number of African countries [5-8] although not all countries have achieved success [9].

Since 2010, PMI has used Roll Back Malaria's methodology to evaluate its malaria interventions [10]. The methodology identifies the need to explain contextual and/or confounding factors (e.g. urbanization, agricultural development, education, burden of other infectious disease, etc.) that may have had an impact on malaria transmission during the past six years of intervention activities.

Key among the list of potential confounders is climate. The intention is to construct a *'plausibility argument"* whereby it can be reasonably assumed "*that mortality reductions can be attributed to programmatic efforts when improvements are found in steps of the causal pathway between intervention, scale-up and mortality trends*" [11]. Here, we seek to ensure that such observed reductions in morbidity and mortality along with changes along the causal pathway are appropriately attributed to interventions climate and other factors as appropriate. Similarly if malaria morbidity and mortality increase, then the role of climate must also be taken into account.

PMI's and partner's evaluation strategy has focused on the measurement of changes in all-cause child mortality (ACCM) and an examination of the plausibility of attributing observed ACCM reductions to National Malaria Control Program (NMCP) interventions. A recent external evaluation of PMI's evaluation methodology [12] indicated that PMI's evaluations should no longer be centered on ACCM. Instead, the external evaluation recommended the use of a range of data sources (including ACCM) in order to assess trends in malaria morbidity and mortality. Such an approach is consistent with the change in PMI's objectives, which now include the reduction of malaria morbidity, in addition to mortality.

Below are four observations, given as examples, which indicate that climate variability and trends potentially confound the attribution of reductions in malaria morbidity and mortality solely to interventions.

- Declines in malaria indicators have occurred in regions where vector populations (*Anopheles gambiae* s.str and *An. funestus*) have declined independently of any known specific malaria intervention - for example in Tanzania [13].

- Declines in malaria indicators have occurred in regions where the dominant vector population has switched from *An. gambiae* s.str to *An. arabiensis* – the latter has a tendency to be more zoophilic and drought tolerant [14]. For example, *Anopheles gambiae* s.str. adult females from indoor collections predominated in Kisumu, Kenya, from 1970 to 1998 (ca. 85%). Beginning in 1999, *An. gambiae* s.str. decreased proportionately relative to *An. arabiensis*, then precipitously declined to rarity coincident with increased bed net ownership as national bed net distribution programs commenced in 2004 and 2006 [15].

- Declines in malaria morbidity and mortality have preceded interventions. These declines have occurred on Pemba Island, Tanzania [16] and in a diverse range of ecological settings in Africa [3].

- Declines in malaria morbidity and mortality have been shown to be associated with drought. For example, declines in malaria due to drought have been observed in Sudan and Eritrea [17, 18]

Note that drought may also be the underlying cause of changes in vector abundance and species as well as observed declines in malaria before interventions are instigated.

- Malaria resurgence has been observed in some areas following unusually high rainfall [2].

2.2 Aim and objectives of this study

The aim of this study is to enable USAID/CDC PMI to account for the confounding effect of climate variability when evaluating the NMCP's malaria interventions.

The study has the following 5 specific objectives:

1. Review the probable impact of climate variability and trends on malaria in different eco-epidemiological settings in Africa, including recent changes in the climate of East Africa (2000-2010) in light of the climate suitability for malaria transmission. This literature will be reviewed in section 3.

2. Establish a methodological framework for removing the confounding effect of climate from routine impact assessment activities carried out by the Monitoring and Evaluation Reference Group. This methodological framework will be summarized in section 4.

3. Identify and collect best data on malaria, intervention and climate in section 5.

4. Analyze the climate data and create new tools that will facilitate climate analysis for future requests in section 6.

5. Provide two short reports (Ethiopia and Tanzania) that contribute to the PMI plausibility argument that is being used in the RBM Monitoring and Evaluation Reference Group (MERG) impact assessment. The Ethiopian short report is presented in section 7 and the Tanzanian report is in section 8.

3 Review of Climate and Malaria in Africa

3.1 Why is Climate Unique?

In addition to malaria interventions a plethora of temporally evolving demographic, epidemiological and health-system factors that are not discussed in this report may play an equivalent or larger role than climate in driving changing morbidity or mortality.

What makes climate measurements unique is the fact that they are (at best) recorded by national agencies according to globally recognized standards at defined, regular time intervals and can be systematically analyzed at the local and global scale allowing comparison across space and time. The following characteristics of climate make it potentially ideal as an additional layer of information for the health sector for application in malaria vulnerability assessments, surveillance and forecasting: its climatology, seasonality, diurnal rhythm and potential predictability at multiple time scales (weather, seasonal, decadal and climate change). Despite their potential, climate data are rarely used in health decision-making. Institutionalized weaknesses in policy development and implementation as well as availability and access to robust climate data and information [19] limit the current capacity of the health sector in Africa [20]. This project signals the type of change needed to effectively use climate information in health policy decision making.

In order to minimize the confounding effect of climate in evaluations of malaria interventions, it is essential to first establish the significance of the underlying relationship of climate variables to malaria outcomes – both in terms of their magnitude and their strength. While in some geographic regions this relationship is relatively easy to quantify, in others it is significantly more challenging. In all areas, quality contemporaneous data on malaria outcomes, the implementation of interventions and climate are needed in order to generate appropriate evidence of the relationship.

However, the use of malaria, intervention and climate data in observational studies pose two distinct challenges i) those which involve the very nature of the data itself and ii) the availability and accessibility of appropriate quality-controlled data. These challenges are outlined in detail in section 3.2 and the means to overcome the widespread lack of appropriate, quality controlled, high resolution climate data is presented in section 5.3.

3.2 Climate – Malaria Interactions

Malaria transmission is highly climate sensitive. Temperature is a significant driver of the development rates of both the *Anopheles* spp. mosquito vector and *Plasmodium* parasite. Rainfall and humidity provide essential environmental characteristics for juvenile mosquito development (breeding sites) and adult survivorship [21]. In general, a relative humidity of 60% or more is deemed necessary for effective malaria transmission [22].

The greatest burden of malaria in Africa is suffered by those living in endemic rural areas. However, populations that live in areas bordering endemic regions are particularly vulnerable to epidemics as their low immunity status makes them susceptible to severe malaria at times when changes in the environment increases its suitability for malaria

transmission [23]. In both highly seasonal and marginal transmission areas, climate (specifically rainfall, humidity and temperature) plays a significant role in determining spatial, seasonal and year-to-year variations in transmission as well as influencing longer term trends. Thus, climate has the potential to confound observational studies of the impact of interventions on malaria outcomes at a range of spatial and temporal scales.

3.3 Rainfall

In Africa, members of the dominant malaria vector species complex, *Anopheles gambiae* s.l. breed in open, sun-light pools. Although irrigation, river pooling and domestic water use may also create suitable breeding sites, the most frequent source of these pools is seasonal rainfall.

Where the environment is sufficiently warm for parasite development to occur, seasonal rainfall is a key determinant of the timing of the malaria transmission season in many parts of the world and especially in semi-arid regions in Africa. Year-to-year changes in rainfall quantity and distribution through the season (e.g. number of days of rainfall) may also be important in driving year-to-year variations in malaria outcomes.

Figure 3-1 Relationship between rainfall and malaria in Botswana

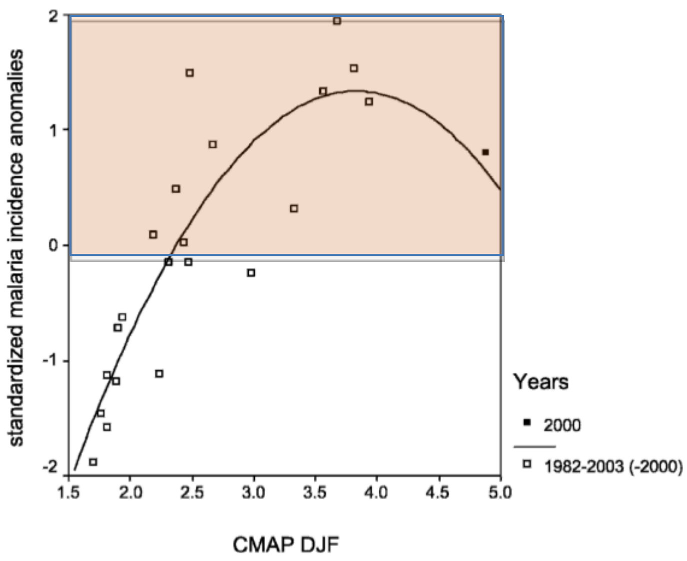

3.3.1 *Rainfall in semi-arid regions*

For example, in lowland semi-arid warm regions of Africa (such as Botswana in southern Africa) where rainfall is the predominant climatic driver of malaria transmission, the relationship between anomalies (deviations from the mean) of national laboratory confirmed malaria incidence data and climate data can be well characterized using simple statistical models Figure (Figure 3-1**Error! Reference source not found.**)[24].

Note that the negative standardized malaria incidence anomalies (<0) have a strong linear relationship with rainfall (CMAP) when compared to positive anomalies. For example, the very high rainfall observed in 2000 (associated with Cyclone Eline) resulted in higher than average malaria anomalies. Overall, however, malaria was lower than what might be expected from a simple linear model and a quadratic model worked best. One reason often cited for this phenomenon is that high rainfall may wash out breeding sites as well as create them, leading to a decrease in malaria transmission [25]. A similar situation was observed in Namibia, where again a quadratic relationship was observed between malaria cases from 9 hospitals in Ovamboland, Namibia and Cold Cloud Duration (CCD), a satellite proxy for rainfall [26] Figure 3-2.

Figure 3-2 Malaria cases and rainfall in Ovamboland, Namibia

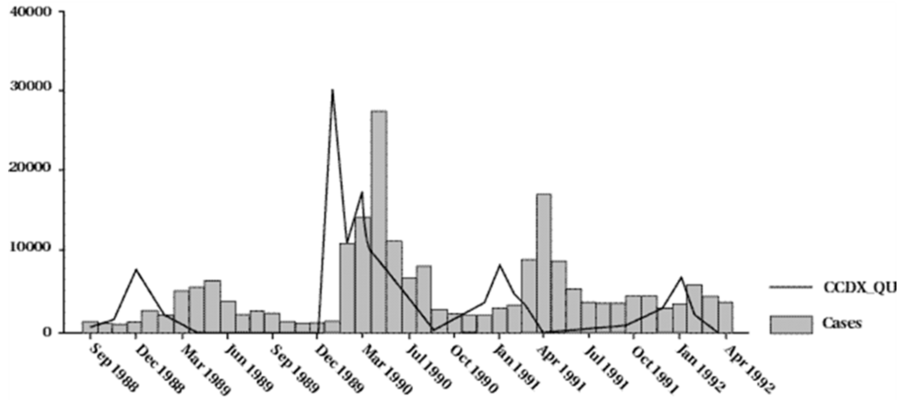

In highly endemic regions such as Sierra Leone (both warm and wet), climate variability has relatively little overall impact on differences in malaria from one season or year to another [27].

3.3.2 Rainfall in highland regions

Altitude also affects rainfall distribution. Moist air which is forced to ascend hills may be cooled below the dew point to produce cloud and rain. A map of average annual rainfall therefore looks very similar to a topographic map. However, mountains at higher altitudes may be relatively dry as clouds have already formed and rain fallen at lower altitudes. The peak rainfall region relative to altitude is called the “pluviometric optimum” (or rainfall maxima). This was noted by the authors of the Highland Malaria (HIMAL) project [28] who extensively reviewed the literature pertaining to the role of climate in the East African highlands. They reported that many highland areas are in fact relatively dry. For example, precipitation on Mount Kenya and Cameroon at 3000 m is only 10–30% of that at 1500m. On Mount Kilimanjaro, rainfall shows a similar pattern but the rainfall maximum is significantly higher, at 2000 m. In Ethiopia, there is a bimodal rainfall distribution with peaks at 2000–2500 m and at 3000–3500 m. Abnormal rainfall events have been shown to cause malaria epidemics in highland areas as long as temperatures remain sufficiently high. Aspect (side of the mountain) and slope (which determines run-off) can also be key determinants at the local level.

3.3.3 Droughts

The strong influence of droughts on lowering or even eliminating malaria for periods of time has been observed in other semi-arid regions including Eritrea [17] and Sudan [18], This relationship appears to be more predictable than the association of malaria epidemics with high rainfall [29], although the latter can be extremely significant - such as the catastrophic malaria epidemics in northeastern Kenya associated with the 1997/8 El Niño [30]. Occasionally, droughts may cause local epidemics of malaria through the creation of breeding sites next to normally ‘free flowing’ rivers. In highly endemic regions such as Sierra Leone (both warm and wet), climate variability has relatively little overall impact on differences in malaria from one season or year to another [27].

3.4 Temperature

3.4.1 The relationship between temperature and altitude

The Highland Malaria Project (HIMAL) was designed to address both the academic and operational aspects of highland malaria in Africa following observations that epidemics were increasing [28]. Amongst other activities the project included the development of a stratification of malaria risk in highland areas, based on spatial modeling of continental datasets for climate and altitude.

In the HIMAL report altitude is recognized as important to malaria control and is used as a key environmental variable to describe the limits of malaria transmission in highland areas. Altitude acts as a proxy for temperature because day-time temperatures decline as altitude increases; a process formally described as the adiabatic lapse rate (ALR). The ALR varies according to the degree to which the air is saturated. In completely unsaturated air, the (dry) ALR is 0.98 °C per 100m, but this rate decreases as saturation increases. In reality air is almost always partly saturated, and while lapse rates of around 0.6 °C per 100 m are most common, spatial and temporal variations in humidity can make lapse rates extremely variable. At night, the situation may be quite different and temperature inversions are not uncommon. The relationship also varies according to latitude and orography.

In Ethiopia for example, altitude is used by the Ministry of Health as a cutoff for endemic, epidemic and non-malarious areas (below <2000, 2000-25000 and >2500 respectively). However because of the limitations described above it is preferable to use the best available estimates of actual temperature rather than simply assume that altitude is a good proxy.

An important constraint in Africa to the use of temperature data in malaria analysis is the limited distribution of meteorological stations recording air temperature and the challenge of interpolating data across complex terrains. Compensation for this paucity of information may be obtained by using satellite-based methods however this is not straightforward. Recently a comparisons between night MODIS temperature surface data with minimum ambient temperature showed that MODIS nighttime products provide a good estimation of minimum ambient over different ecosystems [31].

3.4.2 Temperature and malaria parasite development within mosquito

Temperature affects the intensity of malaria transmission primarily through its effect on the malaria parasite and malaria vector (mosquito). In particular the non-linear relationship of temperature to parasite development means that small changes in temperature can have a big effect on transmission.

The development of the malaria parasite within the vector is referred to as sporogony (extrinsic incubation period). The time necessary for the malaria parasite to complete sporogony can be calculated using Moshkovsky's degree-day-based formulae [32, 33] where:

for *Plasmodium falciparum*: $E = 111 / (T-16)$
for *Plasmodium vivax*: $E = 105 / (T-14.5)$

These formulae indicate that as temperatures increase there is a resulting decrease in the extrinsic incubation period of the malaria parasite. This reduction in the extrinsic incubation period is associated with an increase in malaria transmission intensity. [34]

The following figure depicts the inverse relationship between temperature and the extrinsic incubation period

Figure 3-3 Temperature and the extrinsic incubation period [35]

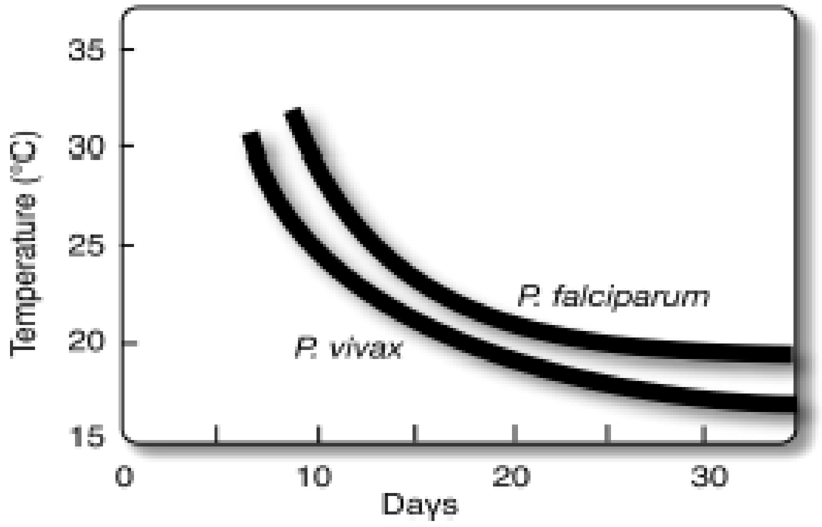

3.4.3 Temperature and mosquito development

There are a number of ways in which temperature affects malaria transmission via its effect on malaria vectors. These include the length of the gonotrophic cycle, vector abundance and vector survival.

The gonotrophic cycle is the process of a female mosquito digesting a blood meal, developing ovaries and laying eggs. As temperatures increase, the time necessary for female mosquitoes to complete the gonotrophic cycle decreases. When the time necessary to complete the gonotrophic cycle decreases, a female mosquito has more opportunities to take blood meals and, therefore, transmit the malaria parasite [36]. A study in Kenya found that an increase in temperature of 1.8 degrees Celsius led to a decrease in the first and second gonotrophic cycles of *Anopheles gambiae* s.l. of 1.5 days (17% decrease) and 1.4 days (27% decrease), respectively [37].

Similarly, studies in Kenya have also shown that higher temperatures of aquatic breeding sites lead to a decrease in the time necessary for larvae to develop into adult mosquitoes [38, 39].

The minimum temperature for mosquito development is between 8-10°C although, the minimum temperatures for parasite development are between 14-19°C. The optimum temperature for mosquitoes activity is 25-27°C, and the maximum temperature for both vectors and parasites is 40°C [40]. It must be noted however that mosquitoes live in micro-climates and may avoid low temperatures by spending much of their time indoors (e.g. in houses or animal pens). They may avoid high day time temperatures by locating

themselves in cool animal burrows or by resting on the outside of a traditional clay pot where the surface is cooled by constant evaporation.

3.5 The relationship of malaria, local climate to global climatic processes

In areas of the world where sea-surface temperatures (SSTs) in the Pacific (i.e., the El Niño-Southern Oscillation, ENSO) are important predictors of climate events, significant correlations between malaria incidence and observed SSTs (including lagged SSTs) have been noted [25, 41].

For instance, in Botswana, an analysis of sea surface temperature and national standardized malaria incidence anomalies revealed a sufficiently high level of predictability [24] to warrant the development of a malaria early warning system which incorporates seasonal climate forecasts [29]. See Figure 3-4.

Figure 3-4 Malaria incidence, rainfall (CMAP) and sea surface temperature in Botswana

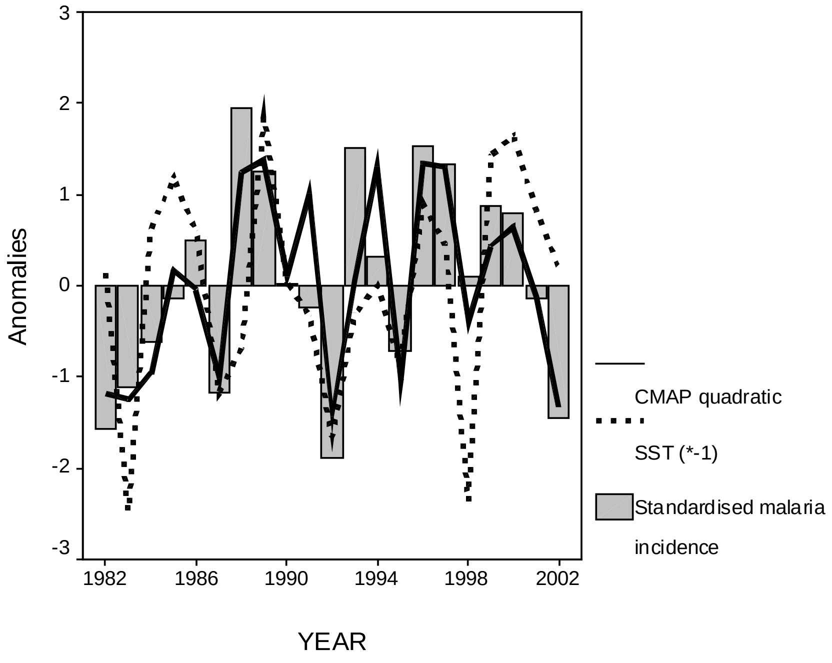

However, as climate is only one of the potential drivers of malaria transmission and associated outcomes and its importance varies across different eco-epidemiological settings, a thorough understanding of the climate and epidemiology of malaria at relevant spatial and temporal scales is needed when assessing the sensitivity of malaria to climate in any particular setting.

3.6 The Climate of Eastern Africa

Many of the countries exhibiting a strong decline in malaria cases are found in Eastern Africa where the environment varies from cool mountains to semi-arid desert to humid coastlines. In terms of its climatology, equatorial East Africa is one of the most complex regions in Africa. The large-scale tropical drivers, which include major convergence zones, are superimposed on other factors including the region's complex topography, large lakes and extensive coastline. Sitting astride the equator, much of the region experiences two rainy seasons occurring when the Inter-tropical convergence zone (ITCZ) traverses the region in its southward and northward migrations. As a result, the climatic patterns are markedly complex and can change rapidly over short distances [42]. For example i) the average amount of rainfall often changes significantly within distances on the order of 10s of kilometers ii) within the region there are areas with one, two and even three seasonal cycles of rainfall and iii) the transition from desert, with rainfall less than 200mm, to rainforest where the annual rainfall is >2000mm happens within short horizontal distances or changes in elevation.

Despite the spatial complexity of the average climate, Nicholson ([42]) observed that the year-to-year variability in rainfall is remarkably coherent across large areas of East Africa with the 'Short' rainy season (typically October to December) showing greater variability than the 'Long" rains, which last from March to May. Seasonal rainfall variations during the short rainy season are largely linked to the El Niño Southern Oscillation phenomena and sea surface temperatures in the Indian Ocean [43]. East Africa is a relatively dry region with average annual rainfall totals in the range of 500m-1500m. In addition, very extensive dry regions (e.g. N.E. Kenya and Somalia) exist as do wetter areas such as those around Lake Victoria.

3.6.1 Evidence of recent warming in Eastern Africa

The potential for warming of highland regions in East Africa as a function of climate change has been an issue of considerable concern to the malaria research and practitioner community since the late 1990s [28] and has been extensively debated by health researchers [44-46] with widely differing perspectives.

Central to this debate has been whether or not a statistically significant upward trend in temperature in the highlands of East Africa has occurred and whether such a rise could account, at least in part, for the observed increase in malaria during the 1980s and 1990s. A substantial constraint to the climate analyses of these and subsequent studies has been the very limited access to a sufficiently long time series of quality controlled daily observations of surface air temperature from meteorological stations. Constraints to accessing such gold standard observations have meant that studies have relied heavily on limited time series of station data, have used data that are inadequately quality controlled or have ignored local ground observations completely in favor of spatially interpolated datasets. However, in the last few years, evidence of regional and local warming in East Africa from detailed analyses of quality-controlled ground based meteorological station data has emerged.

For example, a detailed regional climate trend analysis for East Africa was conducted by Christy and co-authors examining air temperature trends at 60 stations across Kenya

[47]. After spatially interpolating the station-based data, the study reports finding a statistically significant upward trend in minimum temperature in the Kenyan Highlands region. The magnitude of the area-average trend in minimum temperature they identified was about $^{+}$0.15°C per decade, based on an analysis covering the period 1979-2004. However, no statistically significant trend in maximum temperature was found.

For a detailed local analysis, Omumbo and colleagues (including national meteorologists from Kenya and an IRI climate scientist) obtained 30 years (1 January 1979 to 31 December 2009) of quality-controlled daily observations (>97% complete) of maximum and minimum temperature. These data were used in an analysis of trends at Kericho meteorological station, sited in a tea growing area of Kenya's western highlands [48]. After extensive quality control of the data, linear trends were identified via a least-squares regression analysis. An upward trend of approximately 0.2^{O}C/decade was observed in both minimum and maximum temperatures ($P<0.01$).

These 'gold standard' meteorological observations were compared with spatially interpolated temperature datasets that have been developed for regional or global applications and have been previously used in local malaria analyses in the Kenyan highlands [46, 49]. These climate surfaces showed markedly different trends when compared with each other and with the Kericho station observations, reinforcing the perspective that local high quality data are needed for epidemiological studies [50].

Where malaria transmission is constrained by low temperatures, the relationship to malaria is made complicated by the independent and interacting effects of temperature and rainfall. For example, in the Kericho study local rainfall was found to have inverse effects on minimum (night-time) and maximum (day-time) temperatures [48]. Months with above-average (unusually heavy) rainfall were found to be negatively correlated with maximum temperature values ($r = -0.52$, $p < 0.01$) (presumably due to cloud cover restricting daytime sunlight) but positively correlated with minimum temperature (presumably due to cloud cover preventing cooling at night; $r = 0.30$, $p < 0.01$).

Conversely, unusually dry conditions boost maximum temperatures while allowing minimum temperatures to drop. This is an essential point when attempting to link climate variability with malaria: care is needed in choosing the appropriate climate variable to analyze. Often average temperatures (mean of the minimum and maximum) are used in such analyses, which may mask the underlying relationship of malaria transmission to night time (minimum) temperatures – the time when vector mosquitoes are active.

The relationship of local climate variations with larger climate processes, including tropical sea surface temperatures (SST), and El Niño-Southern Oscillation (ENSO) was also assessed by Omumbo and colleagues [48]. To facilitate comparison with other climate variables, monthly departures from the long-term (1980-2009) monthly mean values of maximum temperature, minimum temperature, average temperature and precipitation were computed. To analyze the relationships with El Niño (and La Niña), an 11-month moving average was then applied to the resulting time series as that is the typical time scale of individual ENSO events. They found that temperature variations in Kericho were associated with large-scale climate variations including tropical SST. In particular, minimum temperature showed the strongest relationship (Table 3-1, Figure 3-5) with large-scale climate variations.

Table 3-1 Temporal correlation between climate variables (maximum temperature, minimum temperature and sea surface temperatures)

Variables	Correlation
Tropical sea surface temperatures<br>Kericho minimum temperature	0.77 ***<br>0.46 ***<br>0.36 ***
Tropical sea surface temperatures<br>Kericho maximum temperature	0.62 ***<br>0.24 ***<br>0.14 *

Source: [48]

*Confidence levels, based on a 2-tailed t-test, are shown by asterisks, where *** = 99%, ** = 95% and * = 90%. The three correlation values for each pair of climate variables are those computed for: (top) the 11-month moving average; (middle) monthly values; and (bottom) de-trended, monthly values.

Figure 3-5 Malaria cases and Minimum Temperature at Kericho, Kenya, compared to Global SSTs, Tropical LST and ENSO

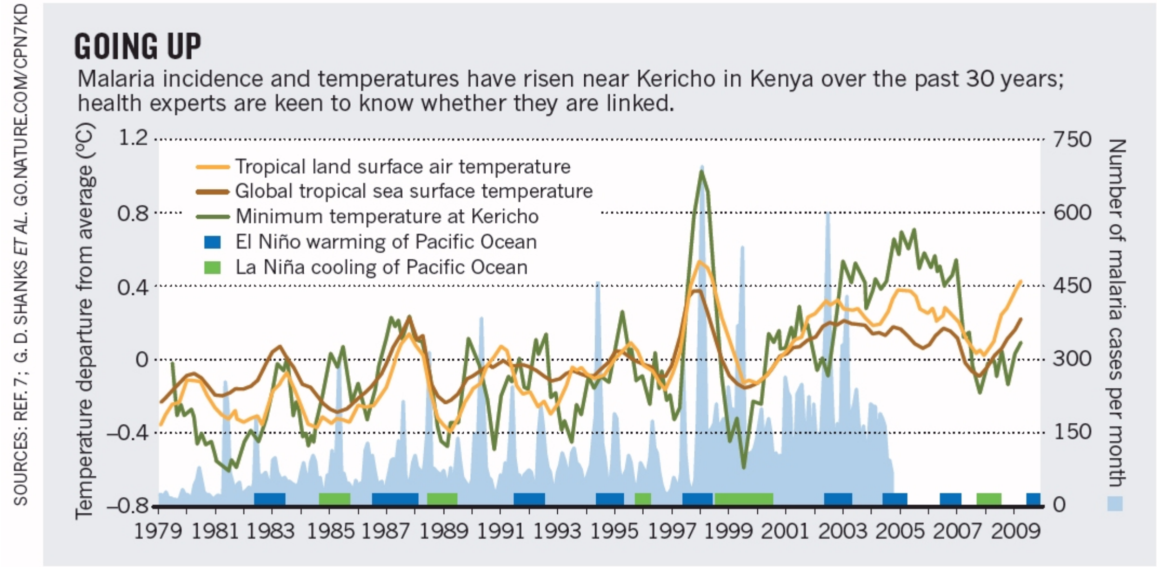

The higher correlation of minimum temperature with sea surface temperatures, when compared with maximum temperature, could be explained by the differential effect that rainfall has on minimum and maximum temperatures (as described above).

During El Niño years, when sea surface temperatures in the Pacific are high, much of East Africa experiences unusually heavy rainfall during the short rainy season (October-December). While El Niño tends to boost temperatures independently of rainfall, this heavy rainfall will moderate the effect and will tend to dampen maximum temperatures while at the same time further increasing minimum temperature. These interactions are described in more detail in following Figure 3-6.

Figure 3-6 Differential impact of rainfall and ENSO on maximum (Tx) and minimum (Tn) temperature

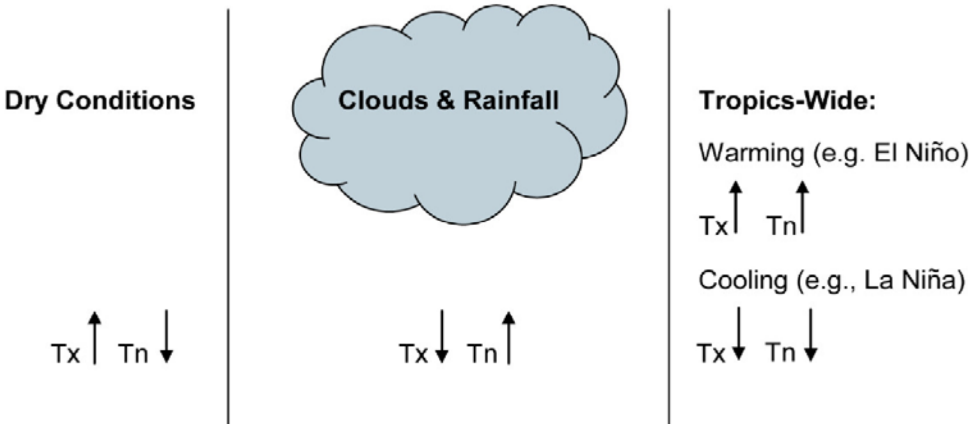

Schematic of the relationship between maximum (Tx) and minimum (Tn) temperatures and unusually dry conditions (left), rainy conditions (center) and in relationship to tropics-wide warming or cooling associated with El Niño or La Niña (right) [48].

3.6.2 Evidence of recent drying in Eastern Africa

Over the past decade East Africa has experienced an increasing frequency of drought events particularly during the “long rains” season, which typically runs from March to May. The most recent of these events, in 2010-11, was considered the most severe in 60 years and triggered a humanitarian crisis that initiated a global response. The spatial extent of the drought, its severity and its likely cause are important as the drought provides a favorable environment for malaria control but could also potentially confound the assessment of the impact of malaria interventions by overestimating their impact. The spatial extent of the recent drying is indicated in Figure 3-7.

Figure 3-7 Percentage change in annual precipitation (c) and March – May precipitation (d) for the period 1999-2009 relative to 1980 – 1998 baseline.

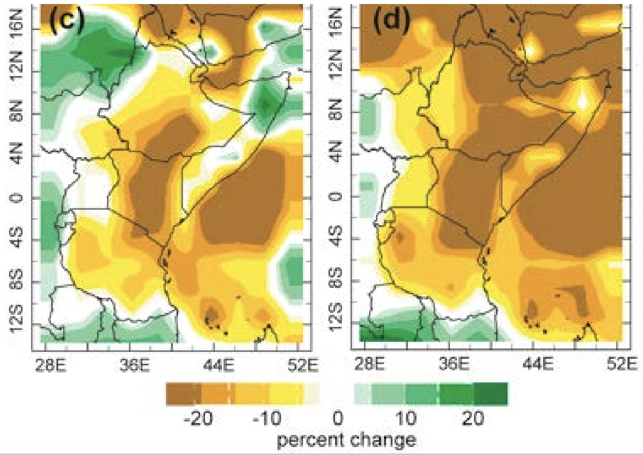

based on GPCP v2.2 data [51]

An abrupt shift towards drier conditions at the regional scale is indicated in Figure 3-8.

Figure 3-8 Time series of March-May rainfall anomalies relative to a 1979-2010 baseline averaged across East Africa land areas

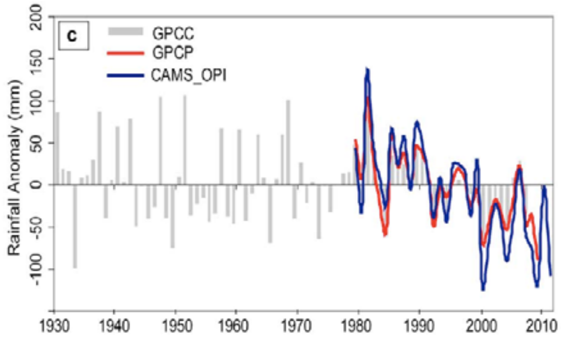

The analysis is based on three sources of precipitation data: from GPCC, GPCP, and CAMSOPI [52].

A reduction of approximately 15% in March - May rainfall has been observed [52, 53] for the region as a whole. While this is substantial, it is not as severe as the protracted Sahel drought where rainfall declines of 30-40% were observed and shown to be mainly driven by sea surface temperatures [54]. Observed changes in malaria indicators in the Sahel as a result of the extended drought included declines in malaria attributed morbidity [55], changes in vector composition and in some cases complete disappearance of *An. funestus* for an extended period of time [56].

3.6.3 Possible causes of the Eastern Africa drought and why they matter

Climate change projections use global climate models (GCMs) to examine the potential effects of increasing concentrations of anthropogenic greenhouse gasses on the Earth's climate. Current projections suggest that the climate of East Africa will become wetter by the end of this century [57]. This divergence from the recent, observed decline in rainfall raises some fundamental questions to climate scientists who provide a number of alternative explanations which are highly relevant to current and longer-term prospects for malaria control and elimination.

For instance, some climate scientists have argued that the increasing frequency of drought in East Africa is associated with an overall downward trend in East African rainfall which has been underway since the 1980s, which in turn is associated with an upward trend in sea surface temperature, especially in the tropical Indian Ocean and therefore potentially connected to climate change [53]. In simplest terms, the argument is that warmer ocean temperatures lead to increased precipitation over the ocean that ultimately robs East Africa of its moisture and rainfall.

In contrast, while not excluding the role of climate change, a recent study by Lyon and DeWitt [52] provides evidence for the role of slowly-varying tropical ocean conditions in generating the recent droughts in East Africa. The study argues that rather than a prolonged decline in rainfall since the 1980's, the long rains of East Africa have instead undergone an abrupt decline that occurred around 1998-99 and that it was associated with similarly abrupt changes in sea surface temperatures, mainly in the tropical Pacific Ocean. Their observational analysis was supported by the results from climate model

simulations, which indicated that drying in East Africa occurred when the model was only responding to conditions in the Pacific.

The apparent discrepancy between recent drying and projections for a wetter future under climate changes raises some fundamental questions. Possible explanations include:

1. The climate models used in making projections are unable to capture key aspects of the climate system and thus indicate the climate of the region will become wetter, while in fact it will dry.
2. The recent drying may be associated with climate variations operating to a large extent independently of those associated with the effects of anthropogenic climate change.
3. The current drying is part of a more complex response to anthropogenic climate change where near term responses differ from longer-term responses because of various feedback mechanisms in the climate system.

Irrespective of which of the above scenarios may be at play, the observation is that the climate of late has been drying and there is the possibility that in the future this trend may reverse (as is normal with natural variations) and this reversal may then be further enhanced by climate change. In short, there is a scientific basis for considering that the climate suitability of East Africa to malaria transmission may increase in the coming decade.

3.7 In summary

Given the analyses summarized above, we contend that the drying and warming trends observed in Eastern Africa could act as confounders in PMI's evaluation of its malaria interventions. In addition, a reversal of the current drying trend (should it occur) could have significant implications for effectiveness of malaria control and elimination programs over the next decade.

4 Methodology

4.1 Method 1: Climate Information Analysis (CIA) Methodology

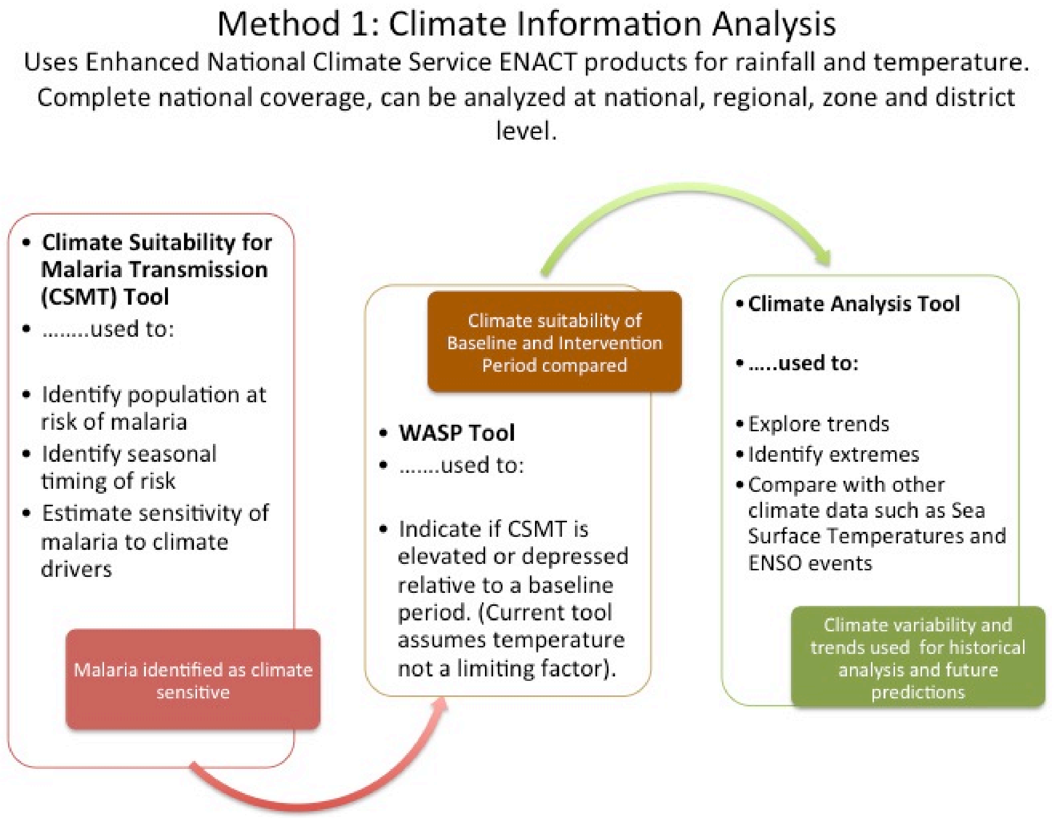

where in absence of malaria and intervention data only climatic factors are investigated. This approach was applied to: i) Ethiopia Section 7 where limited malaria data were available and ii) Tanzania Section 8 where no malaria and intervention data were available.

Step1. The Climate Suitability for Malaria Transmission Tool is used to estimate the degree to which malaria in a given area is climate sensitive. This is a relative analysis and is dependent on the number of months of climate suitability (3 months is more sensitive than 4) and evidence that the area is at the margins of malaria transmission – i.e.. Climate suitability for malaria transmission over the 28-30 year period is <100% for a given month in the main transmission season.

Step 2. The WASP tool is used to estimate the change in rainfall integrated over an area in the intervention period relative to the baseline period. When combined with information on temperature, it is used to estimate whether or not the intervention period is occurring during a time when the climate is more or less suitable for malaria transmission relative to the baseline period.

Step 3. The Climate Analysis Tool can be used to explore trends in the rainfall and temperature data by year, season or month at different spatial scales that may explain an increase or decrease in malaria incidences. Climate variability and its relationship with ENSO can also be explored. This may indicate the likelihood that the region will experience major malaria anomalies during an ENSO event which may impact on future control and elimination activities.

4.2 Method 2: Climate Information, Malaria and Intervention Analysis (CIMIA) Methodology

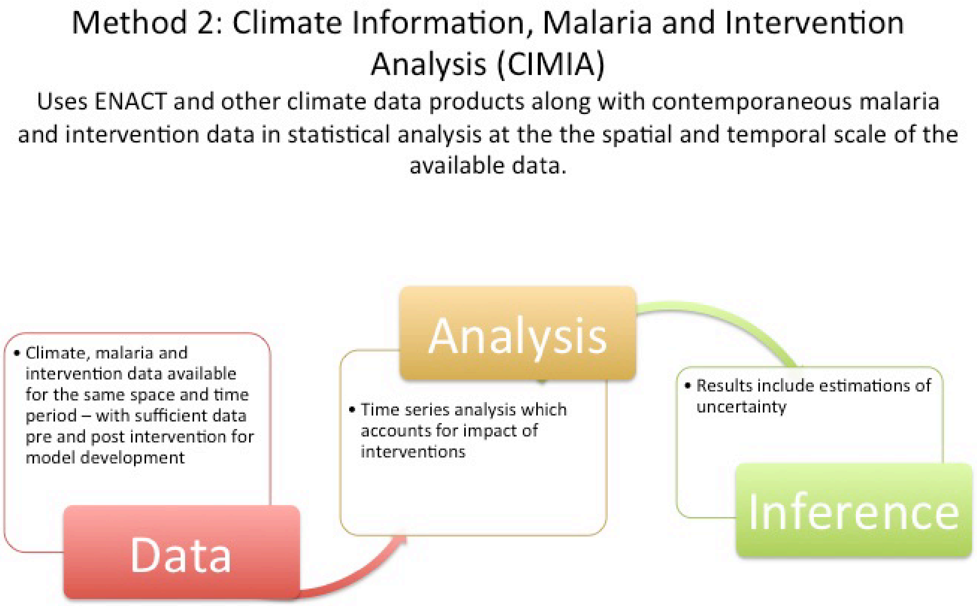

Step 1. Identify region as climate sensitive. This methodology is best applied to an area deemed as climate sensitive for malaria transmission using the CIA method above or through a review of the literature and expert knowledge.

Step 2. Obtain relevant data with sufficient pre and post intervention periods to undertake a meaningful statistical analysis. Five years either side of the intervention period is appropriate but longer may be needed in a region where malaria varies cyclically.

Step 3. Use appropriate statistical methodologies to establish whether, and if so, by how much, interventions have a beneficial impact on malaria morbidity and mortality allowing for the possibility that disease patterns are changing in response to climate variability and trends.

This approach was explored with limited data on malaria and intervention from Ethiopia. The statistical analysis was performed by Peter Diggle's group at Lancaster/Liverpool University (see document "Malaria, Incidences, and Climate Analysis: Ethiopia" for more information, username: PMI, password: pietro). However, due to the limited number of years for malaria and intervention data, it was impossible to statistically identify the contribution of intervention and climate factors on the trends of malaria cases. In order

to implement this approach, this would require longer time-series of cotemporaneous data on all three dimensions – malaria incidence, climate and intervention – observed over a time-period that spans the introduction of the interventions in question. The immediate appropriateness of this analysis is dependent on the quality of national or subnational malaria, climate and intervention data. If the data is not immediately available it is likely that suitable data sets will become available in most countries in the next few years.

5 Data sets

5.1 Malaria data

The use of malaria data in observational studies poses distinct challenges which involve the very nature of the data itself as well as the issue of availability, access relevance, and quality. For example, routine facility-based reporting of cases may be affected by low and variable treatment-seeking, poor routine diagnosis, highly incomplete reporting from facilities and deaths which are compounded by unreliable approaches to malaria-specific mortality estimation.

The nature of survey data also has implications for limitations in the analysis of malaria distributions. The ability to understand the correct distribution of malaria cases is complicated by differential measures (incidence, prevalence, age stratifications etc.) reported across both small and large-scale survey datasets. Further, the ability to combine data from multiple survey sources must consider the epidemiological implications of data quality and biases stemming from incongruous case-definitions and disparate survey coverage.

Other challenges are biological: the relationships between the population prevalence of patent infection (as measured by cross-sectional surveys), clinical disease (a fraction of which is ostensibly measured by routine reporting systems), and malaria-attributable mortality are highly non-linear, extremely biologically complex and only partially understood, and poorly measured empirically.

Given the spatio-temporal nature of climate variability and change across very varied epidemiological setting, the careful use of health surveillance data (either facility based or by administrative unit) in malaria intervention impact analysis [58] has become increasingly important.

Data explored during this study includes:

5.1.1 The DHS (USAID) surveys

These surveys were intended for other purposes and only peripherally concern malaria.. Whilst the reports of these surveys are already available, the malaria data in them is only for children <5 years old and are given to no more than second Administrative level.

5.1.2 Zone level IDSR data from ENHRI (2005-2009).

The database has complete national coverage, is considered of higher quality than HMIS data, and is at the zone level but is of only 5 years duration and therefore insufficient for a robust analysis. In an area of high year to year variability in malaria under normal conditions.

5.1.3 National malaria incidence data from ENHRI (1983-2010).

Created from HMIS data this database is suitable for long term analysis but temporal averaging (year) and spatial averaging (whole country) mean that relationship to climate difficult to establish.

5.1.4 Numerous varied local time series of malaria incidence data.

These were explored during the impacts workshop (see Appendix 1). This helped identify valuable datasets which may be used in the future.

5.1.5 Intervention data from PMI partners in Ethiopia.

These data was accessed for the Oromia Region (2008-2010). However, the short time period covered precluded any detailed analysis.

5.2 Intervention data

As is intimated in all monitoring efforts, consistent and complete data reporting is imperative in correctly evaluating the relationship between interventions and their accompanying covariates. In order to accurately account for and quantify the effect of specific interventions, comprehensive monitoring and evaluation plans are most helpful when they (1) are initiated prior to the commencement of intervention, (2) involve data point collection at multiple and equally spaced time-intervals, and (3) report data at sufficient spatial and temporal resolution. Difficulties in adequately defining association of interventions to a change in the outcome of interest often arise from complications inherent in this monitoring.

In the analysis of Ethiopian malaria trends, primary limitations in time series analyses involved differential dataset lengths of the meteorological-covariate, outcome (malaria), and intervention datasets (see Table 5-1).

Table 5-1 Finding contemporaneous data

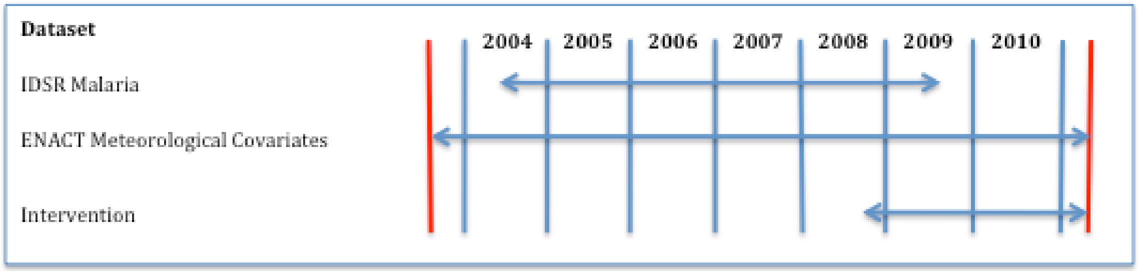

Note: Arrows terminating at red lines denote dataset bounds before or after those in table.

Quarterly reports documenting PMI partner commodity distribution and geographic coverage were provided by Joseph Malone and colleagues, and applied as the known

distribution of Ethiopian interventions. Partner organizations and the interventions they executed are shown in Table 5-2.

Table 5-2 Interventions and associated partners

Intervention	Partner(s)
Rapid Diagnostic Tests	UNICEF, John Snow International
Artemisinin Combination Therapies	UNICEF
Druge Provision (CQ, QN, others)	UNICEF
Long-Lasting Insecticide Treated Nets	UNICEF
Indoor Residual Spraying	None Listed
Social and Behavior Change Communication	Communication for Change
Laboratory Strengthening	International Centers for AIDS Care and Treatment Programs
Case Management/ Epi Surveillance	Integrated Family Health Program
Pharmaceutical Drug Management	Management Sciences for Health
Epidemic Surveillance	Measure III

Quarterly reports from 2008-2011 listed the presence/absence of interventions as provided by the above partners in each woreda of the Oromia region. Fiscal quarters were defined as Q1 (OND), Q2 (JFM) Q3 (AMJ), and Q4 (JAS).
Two variables were created, aggregating interventions as those that vary dependent on malaria increase, and those that vary independent of malaria increase. Interventions available were aggregated as shown in Table 5-3. Some interventions may appear in both categories depending on local control strategies.

Table 5-3 Dependent and independent intervention variables

Int Dependent on Malaria Increase	Int Independent of Malaria Increase
Artemisinin Combination Therapies	Rapid Diagnostic Tests
Other Malaria Drugs	Long-Lasting Insecticide Treated Nets
Pharmaceutical Drug Management	Laboratory Strengthening
Epidemic Surveillance	Social and Behavior Change Communication
Case Management/ Epi Surveillance	Indoor Residual Spraying
Indoor Residual Spraying	

For this analysis it was presumed that the above are all the possible interventions for a specific woreda. Each woreda was given a score for intervention 1 ('dep_inter': dependent on malaria increase) and intervention 2 ('ind_inter': independent of malaria increase), calculated as the proportion of interventions present (coded binary) of the total possible within each scheme. These proportions were then averaged over the available woredas (3rd Order Administrative boundaries: maplibrary.org) for each zone within the Oromia region in each three-month quarter, weighted by woreda population size as of the 2007 census, and extracted over geometries while masking unavailable woredas over the desired zone. An example of the distribution of the intervention scores for the first available quarter can be seen in Figure 5-1. For a more advanced study each intervention might be given a different weight and the impact of an intervention in one time period on subsequent time periods would be included.

Figure 5-5-1 Intervention Score distribution in Oromia

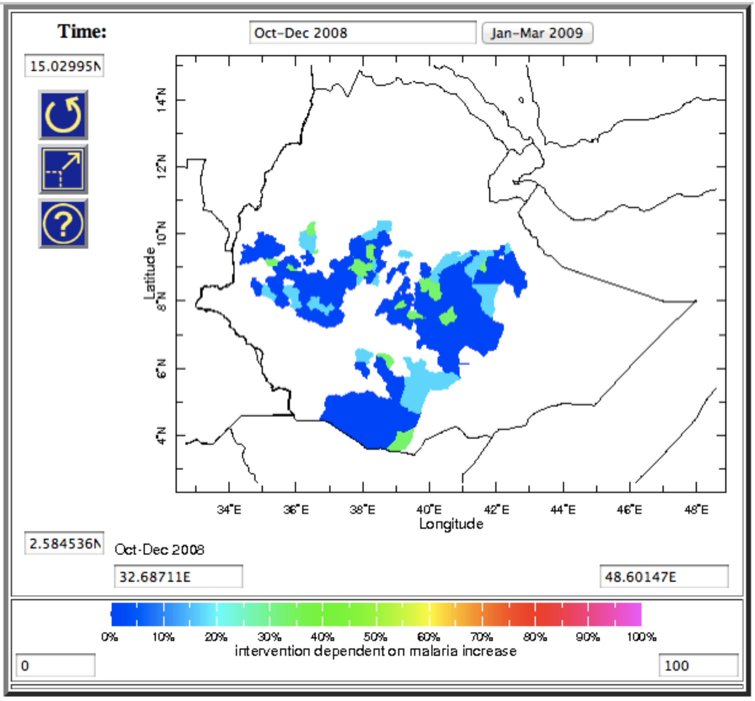

The score for a specific quarter was then applied to the corresponding three months for each zone within the dataset.

Geometries derived from 2nd order administrative boundaries were applied to the intervention datasets for extraction of data at the zonal levels corresponding to the Oromia region.

Additional analytical limitations involved caveats in the intervention dataset available for the country. Intervention variable dataset calculations were limited to those districts (woredas) within a zone that (1) population data were available for, and (2) matched the geographic jurisdictions within the IRI Data Library. Further, intervention scores were applied equally to each of the three months for a given quarter, as detailed contextual information was not provided as would be needed to warrant the application of a "ramping-up" or "waning" of intervention level.

Further, correctly parameterizing interventions is paramount in understanding the correct association of interventions and malaria trends. Intervention data are commonly provided as units distributed (nets) or dispensed (drug regimens and therapies), and complications in analysis arise when it is desirable to parse out the impact attributable to individual or group interventions that overlap in scope and time of effect. However, in the context of this study it is the combined effect of interventions that is of interest.

5.3 Climate data

5.3.1 Meteorological observations

The main source of climate data in African countries is the network of weather stations managed by National Meteorology Agencies (NMAs). In Africa, in general, the number of stations is < 12.5% of what is deemed necessary by the World Meteorological Organization. Even when data exists and is quality controlled the available stations are unevenly distributed with most of the stations located at airports and along the main roads. This imposes severe limitations to the availability of climate information and services particularly to rural communities where these services are needed most. Where records exist, they frequently suffer from data gaps and poor quality and often are not easily accessible due to the dissemination policies of the National Meteorological Agencies (NMAs). As a result many users have turned to global products such as interpolated station data or modeled outputs and satellite data as proxies for what is happening on the ground in specific countries.

5.3.2 Global products

The main advantage of the global products derived from satellite proxies and model reanalysis is the excellent spatial converge: these data are available over most parts of the world at increasingly improved spatial and temporal resolutions. Satellite rainfall estimates now go back thirty years. Over the past decade, a number of rainfall estimate products with high spatial and temporal resolution and near-global coverage have been developed. These products combine precipitation information from multiple sensors and multiple algorithms to produce estimates of rainfall over the globe at spatial resolutions of 0.25° latitude/longitude (or finer) and 3-h temporal resolution (or less). These products are similar in that most of them combine data from passive microwave (PM) and thermal infrared (TIR) sensors. The main differences among them are the manner in which the individual data inputs are combined. These global products, while providing excellent spatial coverage are often not well correlated at small temporal and spatial scales (such as districts) and are frequently inconsistent over long time periods. Some recent products such as Tropical Rainfall Measuring Mission (TRIMM) have demonstrated high levels of skill at the local level but are only available for the last 10 years (2002-2012) [59]. The long term sustainability of TRIMM is also a concern,

5.3.3 Climate change scenarios

Climate change models over Africa are highly uncertain. While many are able to reproduce observed African climate in its general patterns (*i.e.* overall trends, large-scale spatial patterns), they often display strong deviations on the more detailed level. According to Muller et al., research on more specific aspects of African climate, such as climate extremes, is limited and often highly uncertain suggesting that projections on changes in monsoon patterns and cyclones are too uncertain to allow for general conclusions. Despite these deviations and some systematic errors in reproducing observed climate patterns, climate models reproduce the observed climate trend at the continental and regional scale reasonably well. Downscaling projections of coarse climate models for assessments of regional and local climate change impacts adds to the overall uncertainty in climate change projections especially in Africa where the reference meteorological historic database is so weak. Climate projections can therefore be employed to assess the range of possible future climate change, keeping in mind the

shortcomings of climate projections for Africa, and in general but they are not suitable for use in the type of impact analysis reported on here.

5.3.4 Combining ground observations and global products

The problem of data availability and data quality could be significantly improved by combining meteorological station observations with globally available products such as satellite proxies and model reanalysis data [60]. The main advantage of the global products is the excellent spatial coverage. These data are available over most parts of the world at increasingly improved spatial and temporal resolutions. Satellite rainfall estimates for Africa now go back 30 years. The combination of ground-based observations with satellite and/or model information should therefore help to overcome the spatial and temporal gaps in station data while improving the accuracy of the global products. This will alleviate the inadequacy of climate data, particularly for rural Africa where malaria is most prevalent.

5.3.5 Enhanced National ClimaTe Services (ENACTS)

ENACTS is a three-track approach of simultaneously improving “data availability, access and use” is being implement in Africa. ENACTS components include:

1. Availability
    a. Enhanced national (or regional) climatology based on over 30 years of good quality 10 daily rainfall and temperature for every 10 km grid through combining all relevant data from the national observation network with the best global products;
    b. New monitoring products based on observed differences to long-term averages that are superior to currently available alternative products.
2. Access
    a. An online mapping service (using IRI Data Library capacities) installed at the National Meteorological and Hydrological Services (NMHS) allowing visualization and querying and access to the information.
3. Use
    a. Capacity strengthening for national researchers and development professions in the use of the new ENACTS climatology products
    b. Time-sensitive delivery of information for the delivery of the Millennium Development Goals

Figure 5-2 Rainfall products for 2nd 10 day period in April 1996

The top-left panel is the rain gauge data for the 10-day period, while the top-right panel shows a satellite estimate for the same time. The lower-left panel is interpolated rain gauge, and the lower-right panel is combined rain gauge and satellite data – both for the same ten day period. The interpolated gauge follows the overall spatial structure of rainfall as depicted by gauge data, but with significant smoothing.

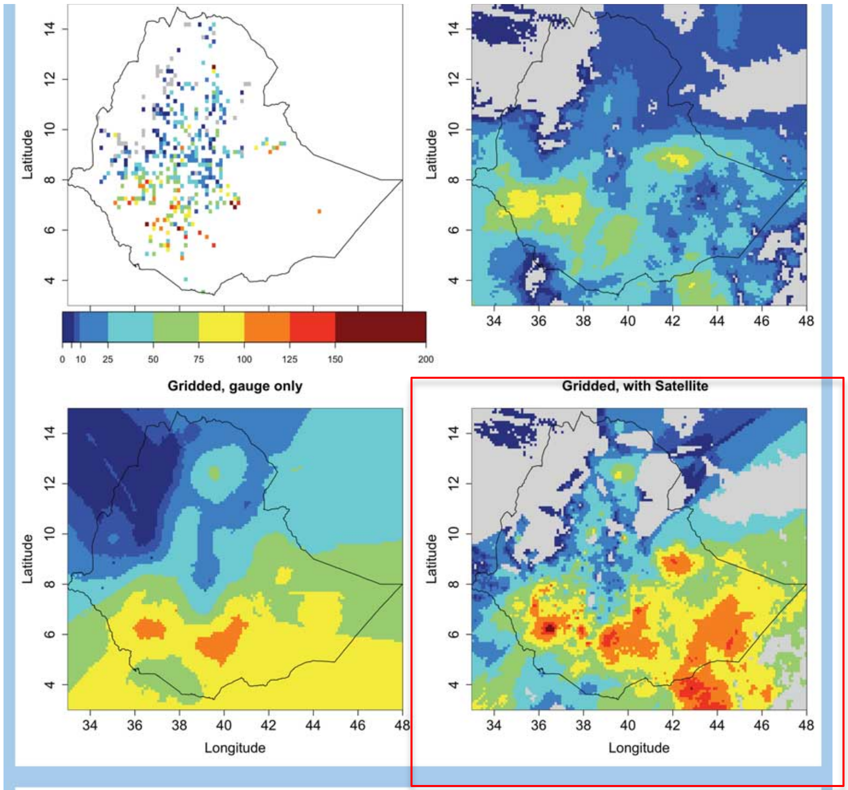

The top-left panel is station data for the 10-day period, while the top-right panel is interpolated station data for the same time. The bottom-left panel is station data combined with 10-day period averages of MODIS LST and elevation for the same period. The bottom-right panel is the topography for reference.

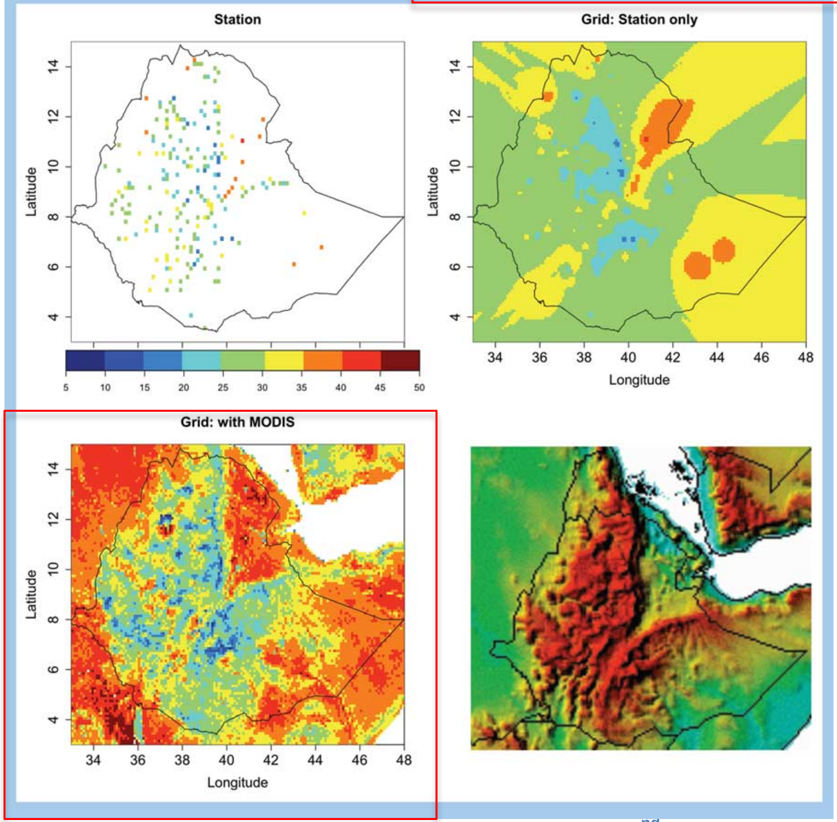

Figure 5-3 Maximum temperature products for the 2nd 10 day period in April 2000

6 New Tools Created

6.1 Climate Suitability for Malaria Transmission Tool

One way to assess the potential sensitivity of malaria in a region to climate is to understand the local drivers of seasonality. A simple approach to describe the seasonality of malaria, to aid localized policymaking and targeting of interventions has been proposed in which sites are defined as having 'marked seasonality' if 75% or more of all episodes of malaria (clinical malaria or confirmed) occurred in six or less months of the year [61]. Another approach is to define malaria seasonality using climate drivers. For example the MARA maps described by Craig and colleagues indicates the likely length and timing of onset of malaria according to a simple model of malaria suitability [62].

The Pan-African Climate Suitability for Malaria Transmission (CSMT) tool was developed originally as a decision tool to support the timing of activities associated with malaria interventions in Africa organized under the auspices of the President's Malaria Initiative [63]. It is an interactive map that displays the number of months during the year when climatological (*i.e*. long-term average) conditions are considered to be suitable for malaria transmission. Suitability is based on empirically-derived thresholds of precipitation, temperature and relative humidity For the purposes of this tool, climatic conditions are considered to be suitable for transmission when the monthly precipitation accumulation is at least 80 mm, the monthly mean temperature is between 18°C and 32°C (*Plasmodium falciparum*) and the monthly relative humidity is at least 60% [62, 64]. A modified tool which uses a 16°C threshold has been developed to represent climate suitability for *Plasmodium vivax* malaria transmission. In practice, the optimal and limiting conditions for transmission are dependent on the particular species of the parasite and vector.

The CSMT tool is well suited to the task of identifying regions where malaria is climate sensitive as it is designed to indicate areas where variability in the climate may affect transmission – e.g. where the disease has a season of four months or less (including whether or not it is bimodal) and where changes in the characteristics of the climate from one year to another are likely to affect the likelihood of malaria transmission. However, the data used in the original published Pan African CSMT tool are based on climate data available from 1951-2000 and do not incorporate data from the last decade which may be more relevant to the current study.

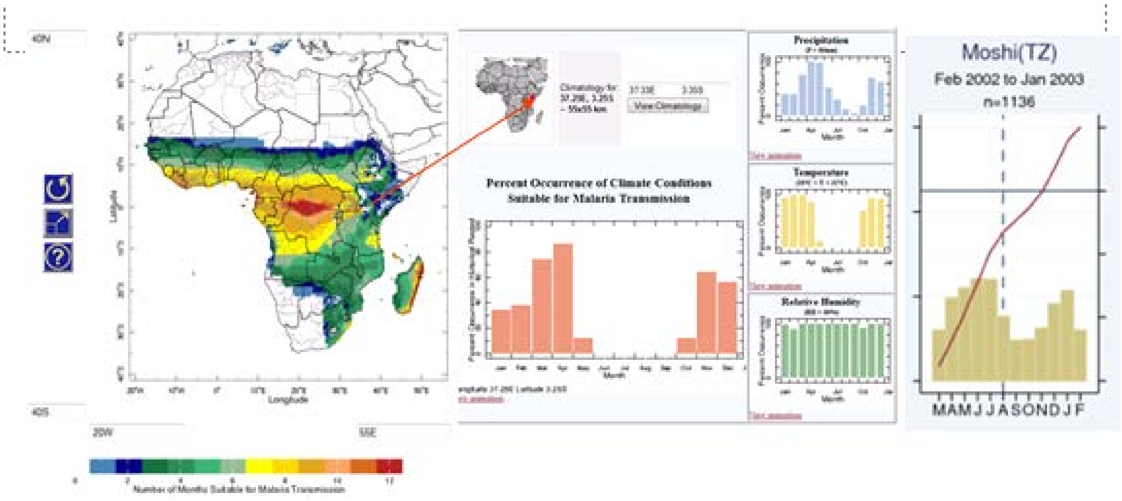

Figure 6-6-1 The Pan African Climate Suitability for Malaria Transmission Tool

The map in Figure 6-1 displays the number of months during the year that are suitable for malaria transmission in Moshi, Tanzania. Suitability is defined as the coincidence of precipitation accumulation greater than 80 mm, mean temperature between 18°C and 32°C, and relative humidity greater than 60 percent. These are rough thresholds that are intended to describe conditions that are suitable for both the development of the *Plasmodium falciparum* parasite and the life cycle of the mosquito vector. The tool provides a simple means for identifying peak transmission periods (and transmission troughs) which will precede key malaria indicators (possibly by one or two months). The figure on the far right of Figure 6-1 represents hospital admissions in Moshi, Tanzania for a single year (Feb 2002-Jan 2003) [61].

The accuracy of the CSMT is based on two factors. The first factor is the degree to which the simple model represents the climatic drivers for the seasonality of malaria at a location. The accuracy of the climate data is the second factor. Using the ENACTS high resolution database for the rainfall and temperature components of the CSMT, it is possible to develop a much higher quality product for Ethiopia and Tanzania.

6.2 WASP Tool

6.2.1 Baselines

Central to any health impact assessment is the concept of a baseline year or baseline period against which changes in outcomes can be measured. If the baseline year (or period) was unusually severe for the particular outcome then achieving change relative to that baseline when the climate risk for malaria is reduced is relatively easy. Conversely, if the baseline year or period experienced a reduced risk relative to the intervention period then it will be harder to achieve positive results.

For example, motivated by the emerging evidence and a widespread malaria epidemic in 1998/1999, Eritrea was one of the earliest adopters of the new push for more aggressive malaria control. With the support of the World Bank and USAID, Eritrea invested $40 million in its national control program. According to reports between 1998 and 2004, substantial reductions in routinely reported clinical malaria cases were described following a scale-up of control measures. By 2004, Eritrea, joined Brazil, Indian and Vietnam as significant success stories in controlling malaria [65]. During this period there was an observed reduction in both the incidence of presumptive malaria in outpatient facilities (83% decline) and in the case fatality of malaria admissions [66]. However,

closer examination of the data indicates that the largest decline in malaria cases was observed between 1998 and 2000 before the control efforts could achieve their maximum effect. This change is explained by the reduction of rainfall after 1998.

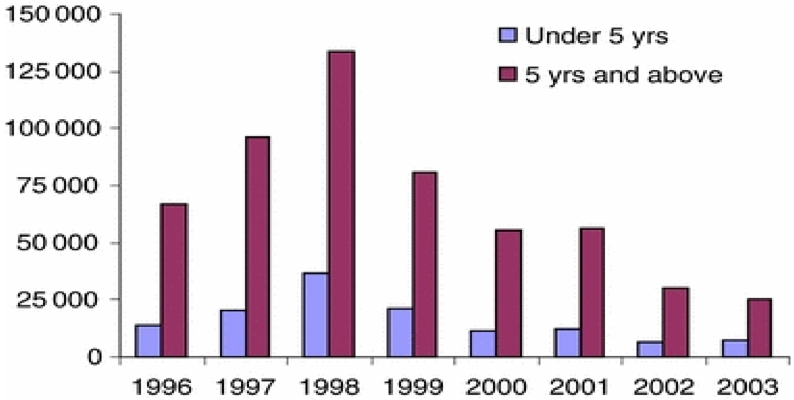

Figure 6-6-2 Malaria morbidity in Eritrea 1996-2003

According to a detailed study, up to 40% of the decline in malaria between 1999 and 2003 could be accounted for in the first instance by climate and environmental factors [17, 67]. Subsequent observations have indicated that malaria control has proven largely robust even when higher rainfall has returned. Malaria control managers in Eritrea routinely use a malaria early warning system to alert them to any likely re-emergence of malaria as a result of high rainfall [67].

6.2.2 Weighted Anomaly of Standardized Precipitation (WASP) with changeable baseline for impact assessment

A simple tool developed by the IRI to analyze droughts using different baseline years indicates this clearly (See Figures 6-3, 6-4).

Figure 6-6-3 Weighted Anomaly of Standardized Precipitation for Eritrea using a 1999 (wet) baseline

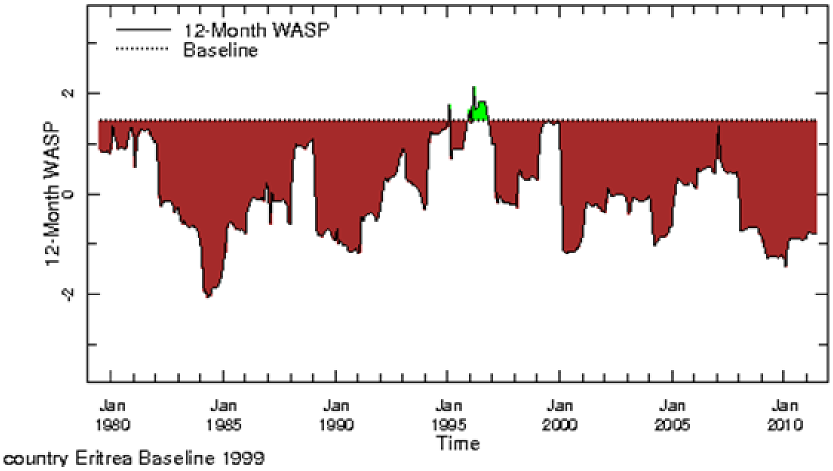

Note the 1996 anomaly. This was the only period above the 1999 WASP baseline in the 28 year time series.

Figure 6-6-4 Weighted Average Standardized Anomaly Rainfall for Eritrea using a 2000 (dry) baseline

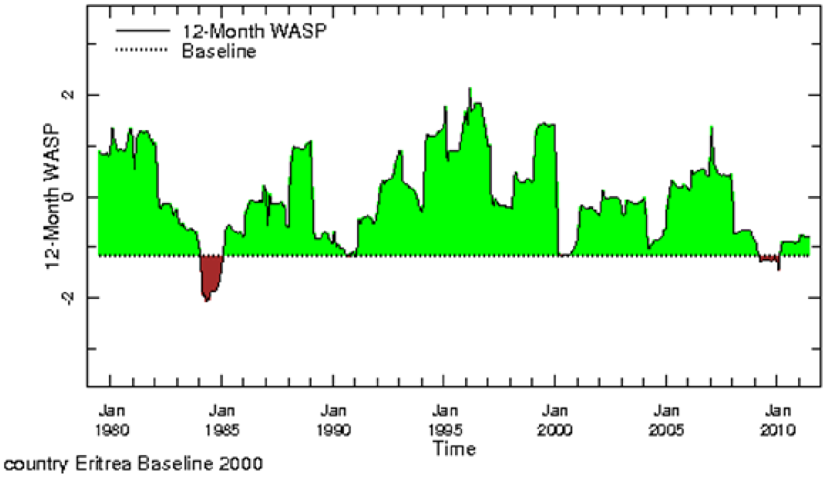

Note the 1984 drought anomaly and the 2009 drought anomalies are the only periods below the 2000 WASP baseline.

This tool was initially developed for analysis at the national level only. For the current online national version the tool uses the following data source: Latest available release of monthly CPC Merged Analysis of Precipitation (CMAP, version 2, combined gauge and satellite estimates) on a 2.5 x 2.5 deg. lat/lon grid. This plot shows a time series of the country-averaged value of the 12-Month Weighted Anomaly Standardized Precipitation (WASP) index calculated using the latest version of the CMAP monthly precipitation dataset for a user-selected country in Africa.

To compute the WASP index, monthly precipitation departures from the long-term average are obtained and then standardized by dividing by the standard deviation of monthly precipitation. The standardized monthly anomalies are then weighted by multiplying by the fraction of the average annual precipitation for the given month. These weighted anomalies are then summed over a 12-month time period in this case, and this result is itself standardized.

The new ENACTS products permit the use of the WASP tool at the national and subnational level.

6.3 Climate Analysis Tool

This tool was developed specifically to analyze the ENACTS products of Ethiopia and Tanzania. It permits an easy analysis of climate variability and trends in rainfall, minimum and maximum temperature over 28-30 years by month, season or year for administrative levels at the national and subnational level.

7 The use of climate information in the assessment of the impact of malaria interventions: Ethiopia

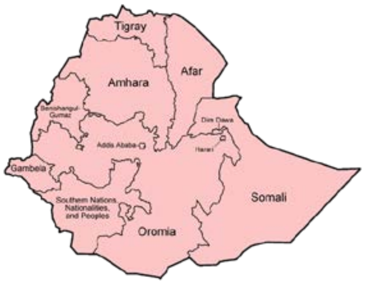

Malaria targets established for Millennium Development Goals and the Roll Back Malaria partnership require measurement of specific malaria outcome indicators in order to evaluate the effectiveness of interventions toward their achievement. The intention is to construct a ‘plausibility argument” whereby it can be reasonably assumed “*that mortality reductions can be attributed to programmatic efforts when improvements are found in steps of the causal pathway between intervention scale-up and mortality trends*”.

Malaria is a complex disease. Its transmission, via *Anopheles* spp. mosquitoes can be highly climate sensitive with temperature being a significant driver of the development rates of both mosquito vector and *Plasmodium* parasite. In addition rainfall and humidity provide essential environmental characteristics for juvenile mosquito development and adult survivorship. Climate has been identified as a one of a number of possible confounders in the evaluation of malaria interventions. Climate information, based on routinely collected data, obtained via globally recognized standards at defined regular time intervals, can be systematically incorporated into malaria analysis at multiple spatial and temporal scales. If climate is not taken into account, then the measurement of achievements may be overly pessimistic in years that experience an elevated climate risk for malaria in relation to the baseline period and conversely overly optimistic when climate risk for malaria is low.

Since 2005 there has been a dramatic increase in malaria interventions in Ethiopia and now the Federal Ministry of Health and its development partners (including USAID) are seeking to assess the impact of these interventions on all-cause mortality as well as malaria specific morbidity and mortality.

In this short report, a climate information analysis methodology involving three steps is used to i) assess the climate sensitivity of malaria in Ethiopia b) indicate changes in climate suitability for malaria risk in the intervention period relative to a given baseline and iii) explore trends and variability in the climate data that indicate the likelihood of large scale climate anomalies associated with ENSO as well as underlying trends associated with sea surface temperatures which are therefore potentially predictable.

7.1 Malaria is climate sensitive in Ethiopia

Ethiopia is located in the Horn of Eastern Africa. The dominant feature of the country is the rugged central highland plateau that varies from 1,290 to 3,000 m above sea level and covers approximately two thirds of the country. A number of rivers cross the plateau notably the Blue Nile rising from Lake Tana. The plateau gradually slopes to the hot and humid lowlands of the Sudan on the west and the hot and dry Somali-inhabited plains to the southeast.

Malaria in Ethiopia is extremely climate sensitive with the majority of the population living in highland or semi-arid regions prone to climate related epidemics. In Ethiopia the determinants of malaria transmission are diverse and localized but temperature (especially minimum temperature), which is linked to altitude is certainly a major limiting factor for malaria transmission in the highland plateau region as is rainfall in the semi-arid regions. Low rainfall is also a feature of highland regions above 1500m. When substantial climate variations occur malaria epidemics can develop rapidly, with devastating effect. A nation-wide epidemic, caused by unusual weather conditions associated with El Niño was documented in 1958. It affected most of the central highlands between1600 m and 2150 m with an estimated 3 million cases and 150,000 deaths [68]. Subsequently, cyclic epidemics of various dimensions have been reported from other

highland areas, with intervals of approximately 5-8 years. Most of these large-scale epidemics have been attributed to climatic variations although other factors such as population displacement, land-use change, drug and insecticide-resistance may also play an important role.

Climatologically speaking Eastern Africa is one of the most complex regions on the African continent. The large-scale tropical drivers, which include several major wind convergence zones, are superimposed on other factors including the regions complex topography, coastline and large lakes. Nowhere is this complexity greater than Ethiopia. The country has two predominant rainy seasons – the main season (Kiremt or Meher) occurs June-September and the small season (Belg) occurs February-May. Research indicates that the Kiremt season is strongly influenced by ENSO (El Niño Southern Oscillation) events that are associated with changes in global sea surface temperatures (SSTs). The Belg season is more erratic and less influenced by global processes such as ENSO. Ethiopia has been suffering from an extended period of drought over the last decade with a 15-20% observed decline in rainfall and evidence of widespread warming which is related to changes in seas surface temperature [53] the exact nature of which is unclear [52].

The extent to which global sea surface temperatures and ENSO (El Niño and La Niña) may drive local meteorological phenomena can be observed in an analysis from neighboring Kenya (Figure 7-1). While El Niño years favor a rise in minimum temperature at Kericho, La Niña years are associated with a decline.

Figure 7-1: Time series of LSTs and SSTs compared to Minimum Temperature from Kericho, Kenya (1979-2009)[48]

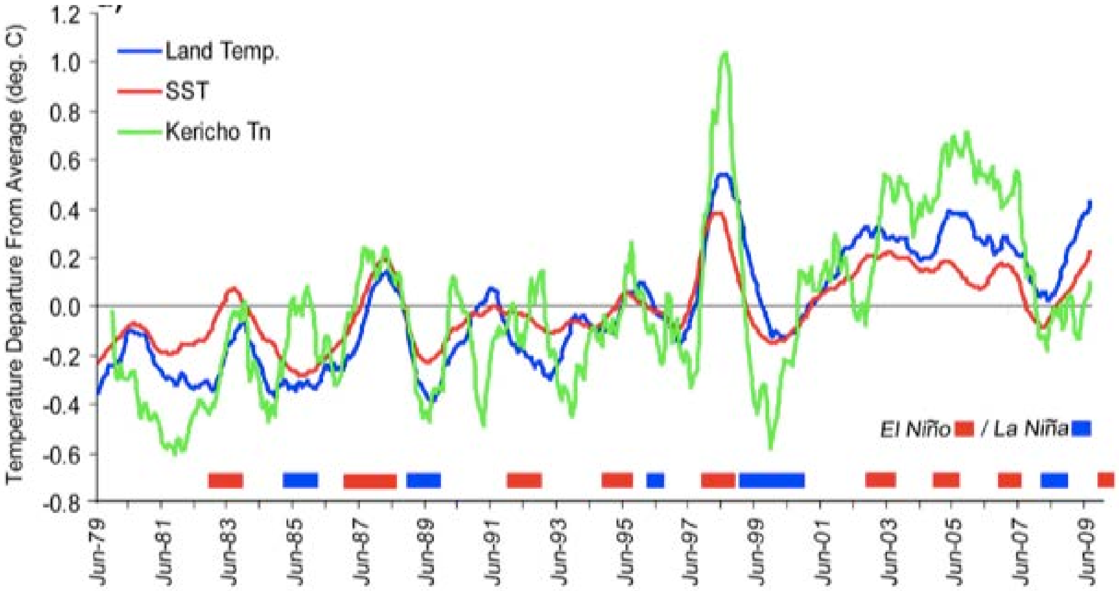

This relationship (warming minimum temperature in Kericho, and warming sea surface temperature) is likely common in parts of Ethiopia but a detailed analysis by location is required to ascertain where this relationship is robust.

ENSO events have long been associated with malaria epidemics in Ethiopia (e.g. 1988/9; 1991/2) and a relationship with global Sea Surface Temperatures (SSTs) is considered likely. However, the relationship is less clear in a national analysis as different regions of Ethiopia respond differently to ENSO and the Belg and Kiremt season may not be uniformly impacted.

7.2 ENACTS Climate products for Ethiopia

While global climate data and products can be used for analysis at the national scale there is a need for high quality high spatial and temporal resolution data for analysis at the sub-national scale. In response to this challenge Ethiopia has implemented a new climate data and dissemination process (Enhanced National Climate Services: ENACTS) [60]. These ENACTS products combine locally calibrated satellite rainfall estimates derived from METEOSAT and all available quality controlled ground-based meteorological station gauge data (more than 600 stations) available in Ethiopia for the period (1983-2010). The new climate time series includes minimum and maximum temperature generated by combining station measurements (from about 300 stations) with NASA's MODIS land surface temperature estimates data and digital elevation model.

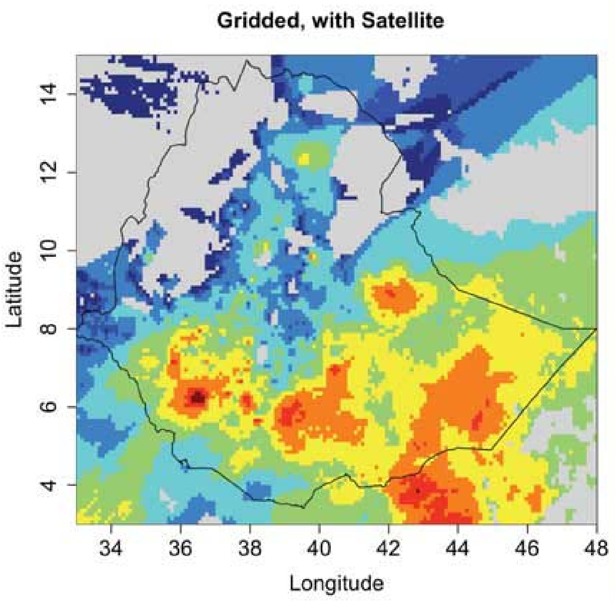

Figure 7-2 ENACTS rainfall product for Ethiopia– example of one 10 day period.

The ENACTS data, derived products, and the related web-based services are unprecedented in Africa and many parts of the world. This quality assured data set has been made available by the National Meteorological Agency of Ethiopia (http://www.ethiomet.gov.et/) and is suitable for robust analysis at the national, regional, zonal and woreda level.

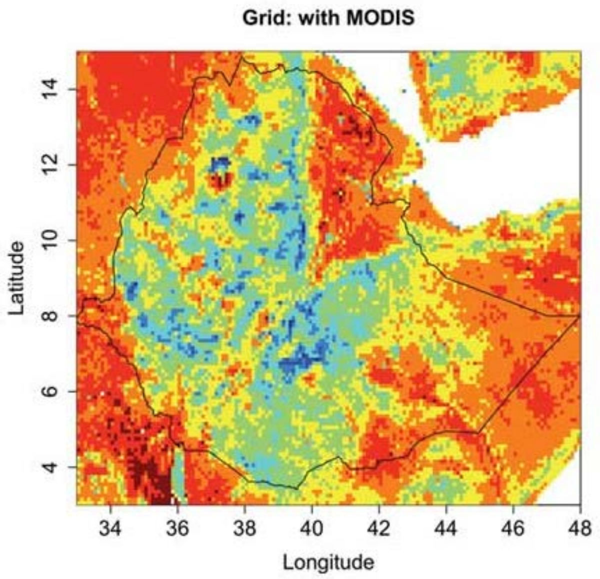

Figure 7-3: ENACTS Temperature product for Ethiopia – example of one 10 day period.

The new ENACTS database and the IRI Data Library data management, analysis and visualization capacities enable the climate drivers of malaria seasonality, variability and trends in Ethiopia to be observed at multiple temporal and spatial scales (e.g. by month, season, year, grid point, woreda, zone and region). As a first step simple averaging and mapping of climate data reveals general patterns.

The Climate Suitability Tool for Malaria Transmission (CSMT) [69] is an interactive mapping tool that interrogates the ENACTS database and then displays the number of months during the year when climatological (i.e. long-term average) conditions are considered to be suitable for malaria transmission. Suitability is based on empirically-derived thresholds of precipitation, temperature and relative humidity. These are a) monthly precipitation accumulation is at least 80 mm, b) monthly mean temperature is between 18°C and 32°C (*P. falciparum*) and c) monthly relative humidity is at least 60%. In practice, the optimal and limiting conditions for transmission are dependent on local conditions (including surface water) and the particular species of the parasite and vector.

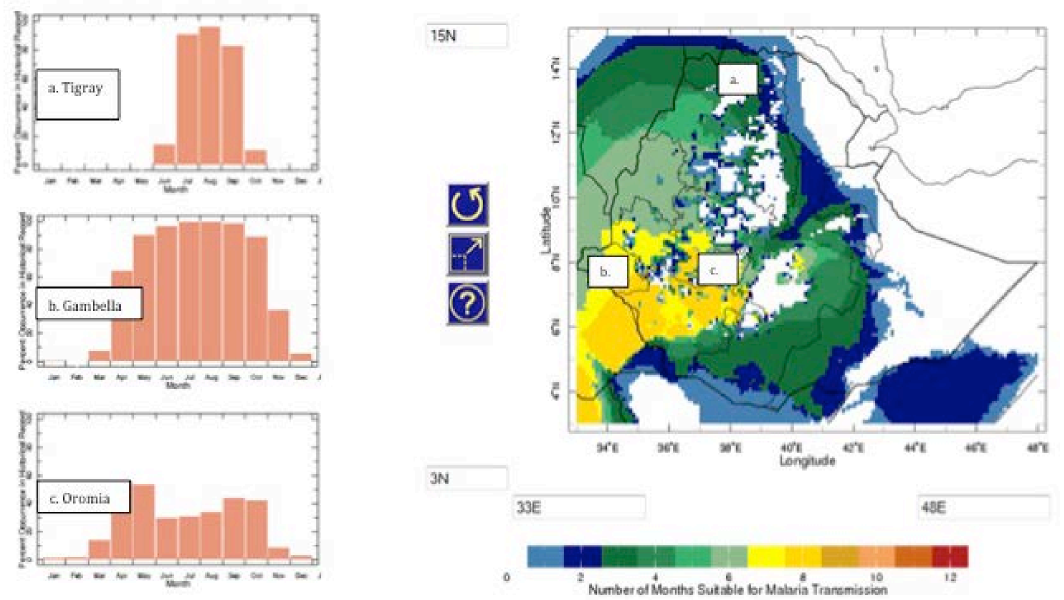

Figure 7-4: CSMT for Ethiopia using ENACTS products indicating highly seasonal transmission in green areas such as Tigray, a long single transmission season in the warm lowlands of Gambela and a bio-modal seasonality in the highly varied Oromia region

7.3 Baselines

Central to malaria intervention impact assessment is the concept of a baseline year or baseline period against which changes in outcomes can be measured. If the climate risk for malaria in the baseline period) was unusually severe then achieving change relative to that baseline is relatively easy. Using the Weighted Average Standardized Precipitation Tool (WASP) it is possible to explore changes in rainfall integrated over time and over a specified region for both a baseline and intervention period. Where temperature is not a constraint to malaria transmission this tool may provide a good estimate of climate risk for malaria, for example Oromia (Figure 7-9).

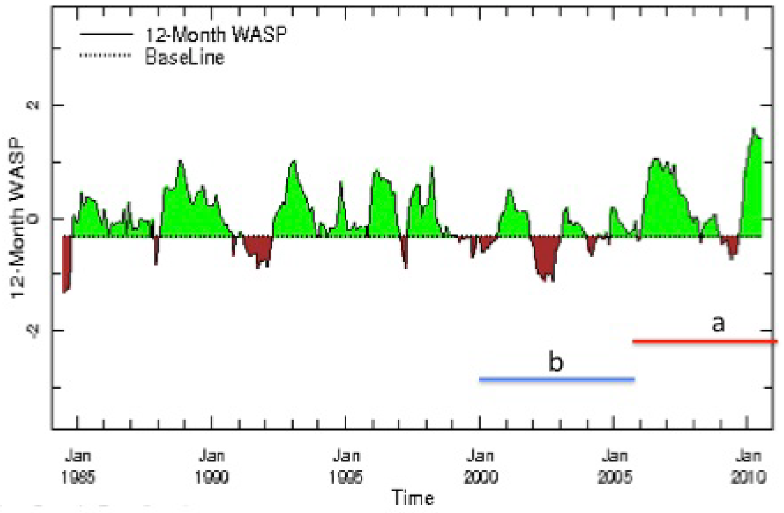

Figure 7-5: ENACTS - rainfall analyzed using the WASP tool for Oromia Region. The green areas are wetter than the baseline period whereas the brown areas are drier.

Intervention period a) (2006-2010) and baseline period b) (average of 2000-2005)

Additional WASP results for each region in Ethiopia and each zone in Oromia can be seen in Figure 7-8 & Figure 7-9.

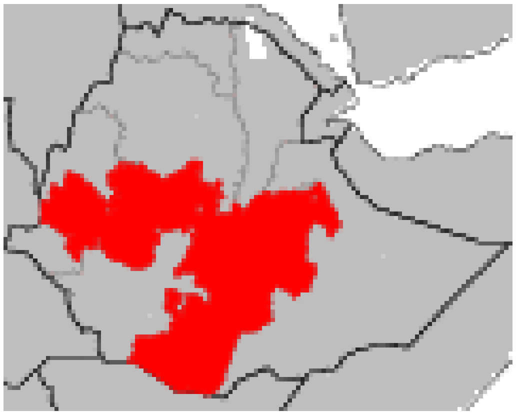

Oromia (similar information is available for all regions, zones and woredas). The Oromia Region is home to approximately 35% of Ethiopia's people and is the source of more than a third of the countries confirmed malaria cases. The other most populous regions are Amhara and SNNPR. Recent climate trends for the region's two rainy seasons, the Belg and Meher (Kiremt) are set out below. Substantial warming can be observed in both seasons over the time period 1983-2010. This warming may make highland regions more susceptible to malaria. An extended drought period from 1997-2009 for the Belg season can be observed. The year 2010 was extremely wet in both seasons and also had high minimum temperatures (compared to the long term average).

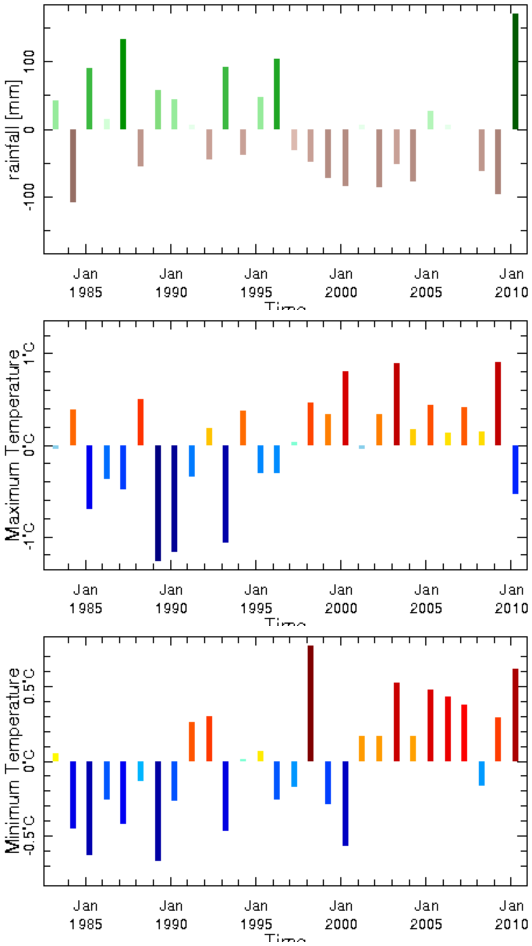

Figure 7-6abc Trend in rainfall (top) max T (middle) and min T (bottom) for the Belg rainy season (Feb-May).

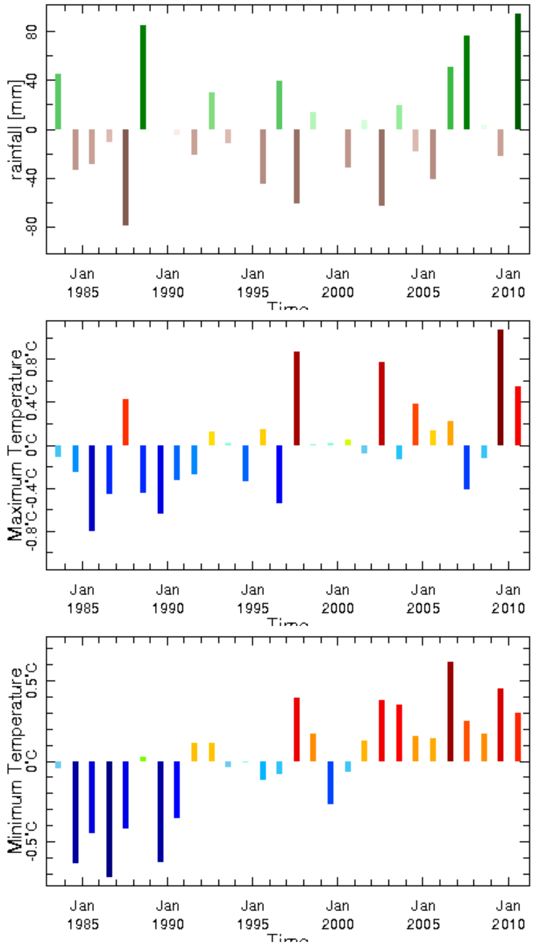

Figure 7-7abc Trend in rainfall (top) max T(middle) and min T (bottom) for the Kiremt rainy season (Jun-Sep).

7.4 Findings from Analysis

- The new quality assured ENACTS temperature and rainfall products for Ethiopia, (28 years; 10 day and 10km) resolution are suitable for analysis at different time-space scales (e.g. woreda, zone, region and country, dekad, month, season, year).

- The Climate Suitability for Malaria Transmission Tool used in conjunction with ENACTS products reveals that >75% of the populated areas of Ethiopia are climate sensitive for malaria transmission: with both temperature and rainfall having a role. The country is therefore classified as extremely sensitive.

- The WASP Tool explores changes in ENACTS rainfall integrated over time and over a specified region for both a baseline and intervention period. Results for Oromia indicate a wetter intervention period (2005-2010) relative to a baseline period of 2000-2005. Given temperature also increased during the intervention period the climate risk for malaria is deemed more severe in 2006-2010 than during the baseline period. These results are replicated across the highly populated regions of the central highlands including Amhara and SNNPR.

- The Climate Analysis Tool using ENACTS temperature shows there is evidence of significant warming in Oromia and other highland areas over the 28 year period. Warming appears to be inversely related to drying. This warming is highly significant (approx. 0.3°C per decade) and could (amongst other factors) account for increases in malaria observed at higher altitudes (e.g. in 2003).

- A regional drought has persisted in East Africa including parts of Ethiopia through much of the last decade – especially during the long rainy season. (Kiremt). In the most populated areas, where >75% of malaria cases are found (Oromia, Amhara and SNNPR) the drought appears to have been more significant in the period 2000-2005 with some recovery in recent years (2006-2010). This suggests that drought cannot be the reason for the decline in malaria observed in Ethiopia during the intervention period 2006-2010.

- Observed increases in malaria in 2009 and 2010 in partner reports may be associated with a particularly favorable climate in some regions of Ethiopia.

- Policy makers and control managers should be aware that cyclical swings in the climate are likely in the future and while the extended regional drought may continue it is also possible for a return to much wetter conditions in the near future.

Figure 7-8 Regional WASP analysis for Ethiopia using ENACTS products using 2000-2005 baseline

Figure 7-9 Zonal WASP analysis for Oromia, Ethiopia using ENACTS products with a 2000-2005 baseline

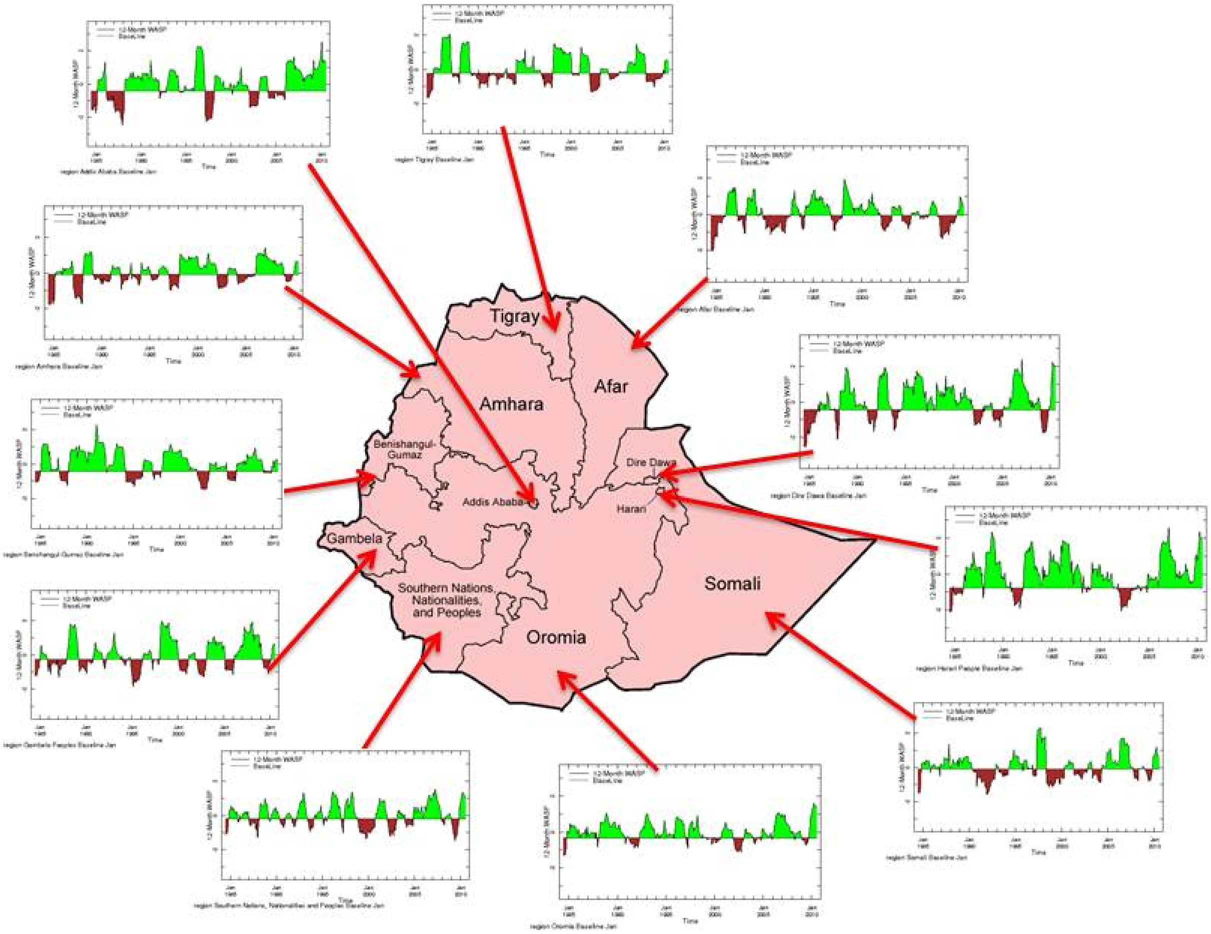

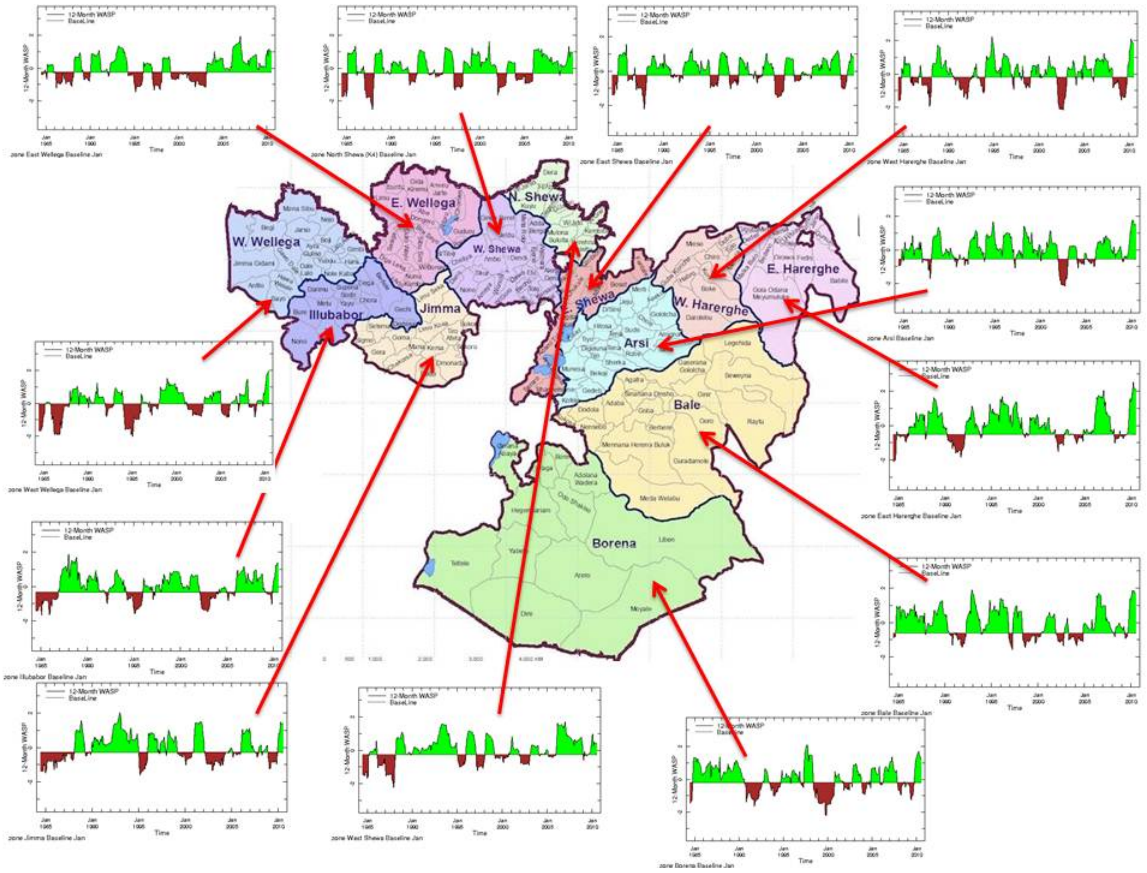

WASP analysis based on ENACTS products using a 2000 – 2005 baseline

7.5 In summary

In Ethiopia, the relationship between malaria and climate is very significant and complex. The most obvious impact is that of the changing epidemiology of disease at increasing elevations, associated with both changes in temperature and rainfall. There is a high risk that impact assessments that do not incorporate climate will overestimate or underestimate the impact of malaria interventions. Incorporating climate data into an impact evaluation must be undertaken at multiple scales in order to account for local complexity. Evidence to date suggests that climate risk for malaria has increased during the intervention period when compared with the baseline period.

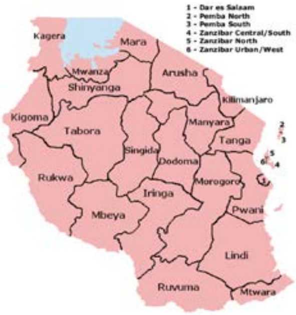

8 The use of climate information in the assessment of the impact of malaria interventions: Tanzania

Malaria targets established for Millennium Development Goals and the Roll Back Malaria partnership require measurement of specific malaria outcome indicators in order to evaluate the effectiveness of interventions toward their achievement. The intention is to construct a 'plausibility argument" whereby it can be reasonably assumed "*that mortality reductions can be attributed to programmatic efforts when improvements are found in steps of the causal pathway between intervention scale-up and mortality trends*".

Malaria is a complex disease. Its transmission, via *Anopheles* spp. mosquitoes can be highly climate sensitive with temperature being a significant driver of the development rates of both mosquito vector and *Plasmodium* parasite. In addition rainfall and humidity provide essential environmental characteristics for juvenile mosquito development and adult survivorship. Climate has been identified as a one of a number of possible confounders in the evaluation of malaria interventions. Climate information, based on routinely collected data, obtained via globally recognized standards at defined regular time intervals, can be systematically incorporated into malaria analysis at multiple spatial and temporal scales. If climate is not taken into account, then the measurement of achievements may be overly pessimistic in years that experience an elevated climate risk for malaria in relation to the baseline period and conversely overly optimistic when climate risk for malaria is low.

Since 2005 there has been a dramatic increase in malaria interventions in Tanzania and now the Ministry of Health and its development partners (including USAID) are seeking to assess the impact of these interventions on all-cause mortality as well as malaria specific morbidity and mortality.

In this short report, a climate information analysis methodology involving three steps is used to i) assess the climate sensitivity of malaria in Tanzania b) indicate changes in climate suitability for malaria risk in the intervention period relative to a given baseline and iii) explore trends and variability in the climate data that indicate the likelihood of large scale climate anomalies associated with ENSO as well as underlying trends associated with sea surface temperatures which are therefore potentially predictable.

8.1 Malaria is climate sensitive in Tanzania

Tanzania is located in Eastern Africa. It has a very varied geography – extending from the Indian Ocean coastline in the East to the humid Lake Victoria region in the West. Most of the country has a tropical climate with cooler regions limited to the highland areas associated with Tanzania's volcanic mountain range (including Kilimanjaro) in the northwest and the highland plateau in the south central region.

Malaria in Tanzania is mostly highly endemic and seasonal. In this country the key environmental determinant of malaria transmission is rainfall, especially in the semi-arid central region where the transmission season is less than three months. Temperature (especially minimum temperature), which is linked to altitude, is also important in the highland regions. Low rainfall is also a feature of highland regions above 1500m. When substantial climate variations occur malaria epidemics can develop rapidly in these specific regions, with devastating effect as was notable in Kagera, a region that was subject to widespread malaria epidemics in 1997 and 1998 following the unusually strong El Niño.

Climatologically speaking Eastern Africa is one of the most complex regions on the African

continent. The large-scale tropical drivers, which include several major wind convergence zones, are superimposed on other factors including the regions complex topography, coastline and large lakes. Tanzania lies within this complex climate system and as a result the country has two major rainfall regimes in different parts of the country. In the south, west, south-west and central regions of Tanzania the rainfall is unimodal - there is one rainy season, which occurs between December and April (called Kifuku). In the northern and eastern regions of the country the rainfall is bimodal, with two rainy seasons. The 'short rains' or Vuli last from October to December, and the 'long rains' or Masika last from March to May.

Research indicates that the Oct-Dec season is strongly influenced by ENSO (El Niño and La Niña) events that are associated with changes in global sea surface temperatures (SSTs). As a result the climatic patterns across the country are mixed with locally complex regions such as the northern highlands or the coastal region. This makes the use of climate information from gauges at meteorological stations particularly challenging as what may be measured in one area cannot necessarily be applied to an area nearby. The extent to which global sea surface temperatures and ENSO (El Niño and La Niña) may drive local meteorological phenomena can be observed in an analysis from neighboring Kenya (Figure 8-1). While El Niño years favor a rise in minimum temperature at Kericho, La Niña years are associated with a decline.

Figure 8-1 Time series of LSTs and SSTs compared to Minimum Temperature from Kericho, Kenya (1979-2009)[48]

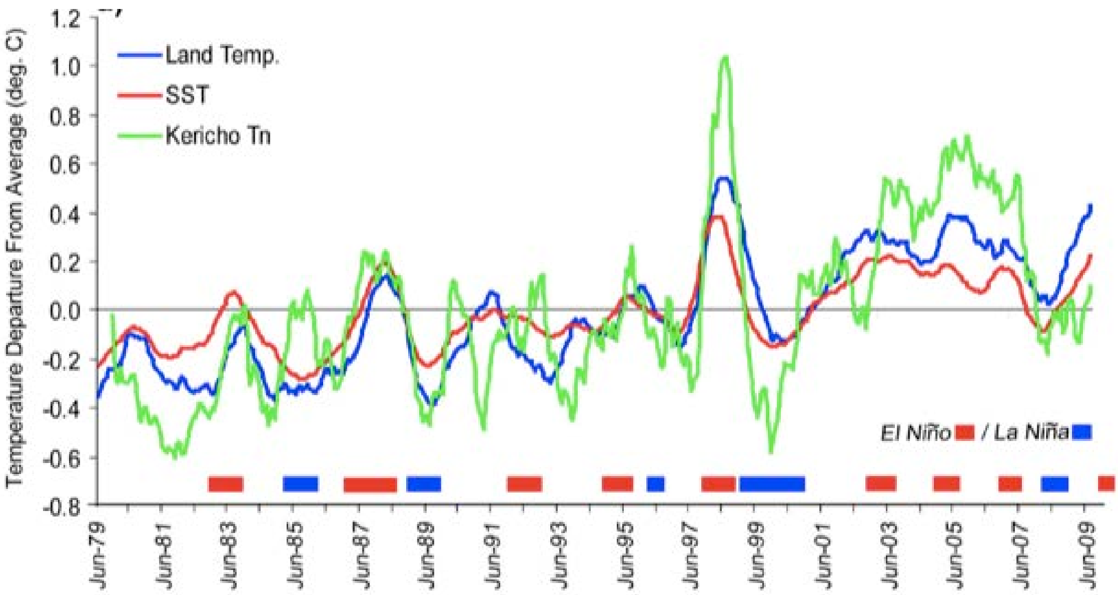

This relationship (warming minimum temperature in Kericho, and warming sea surface temperature) may also be found in parts of Tanzania where a strong response to ENSO is evidenced by very high rainfall in 1997/98.(see Figure 8-5 below). However the relationship is not uniform across the country.

ENSO events have been associated with malaria epidemics in Tanzania. Uddenfeldt-Wort and colleagues demonstrated that the risk of delivering a low-birth weight baby in the first pregnancy increases approximately 5 months following a malaria epidemic. Their analysis indicates that an epidemic which occurred in Kagera resulted in large birth weight differences between primigravidae and multigravidae occurred, related to ENSO's impact in 1997–1998 [70]. Conversely, Lindsay *et al*, who compared the level of malaria infection in children before and following the 1997–1998 El Niño in the Usambara mountains of NE Tanzania, found that even though El Niño led to more abundant rainfall, fewer malaria cases were reported following this

event than in the previous year, suggesting that heavy rainfall may have washed away mosquito breeding sites [71] although lower temperatures may also have been important.

8.2 Climate data and products for Tanzania

While global climate data and products can be used for analysis at the national scale there is a need for high quality high spatial and temporal resolution data for analysis at the sub-national scale. In response to this challenge Tanzania has implemented a new climate data and dissemination process (Enhanced National Climate Services: ENACTS) [60] (Dinku, Hilemariam et al. 2011). These ENACTS products combine locally calibrated satellite rainfall estimates derived from METEOSAT and all available quality controlled ground-based meteorological station gauge data (more than 200 stations) available for Tanzania for the period (1983-2010). The new climate time series also includes minimum and maximum temperature generated by combining station measurements (from about 50 stations) with NASA's MODIS land surface temperature estimates data and a digital elevation model.

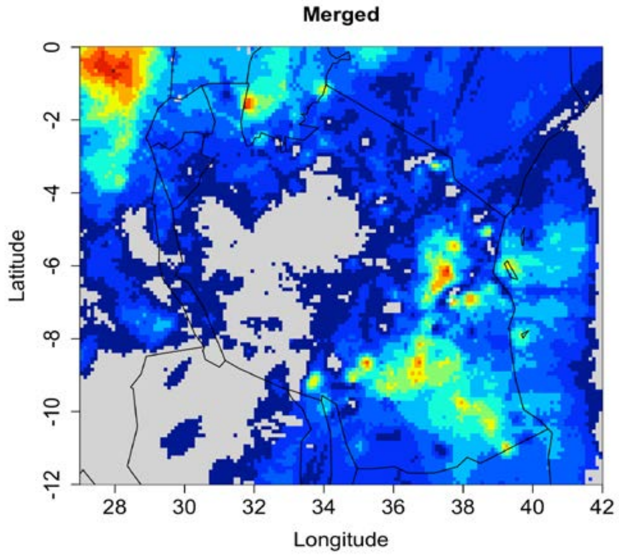

Figure 8-2 ENACTS rainfall product for Tanzania – example of one 10 day period

The ENACTS data, derived products, and the related web-based services developed in Ethiopia and Tanzania are unprecedented in Africa and many parts of the world. The quality assured data set used in this analysis has been made available by the Tanzanian National Meteorological Agency (http://meteo.go.tz/) and is suitable for robust analysis at the national, regional and district level.

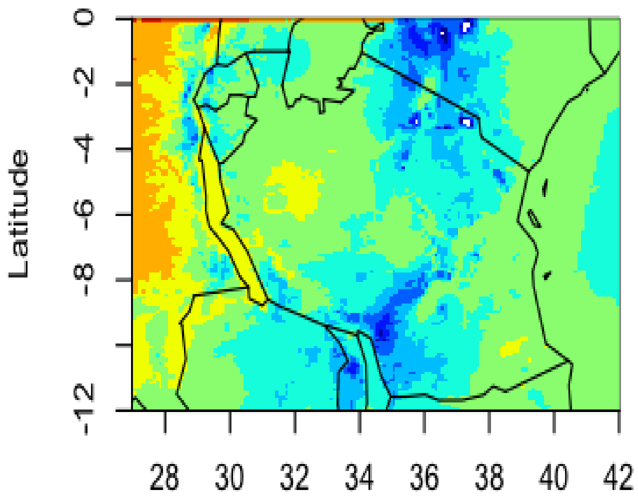

Figure 8-3 ENACTS temperature product for Tanzania – example o one 10 day period.

The new ENACTS database and the IRI Data Library data management, analysis and visualization capacities enable the climate drivers of malaria seasonality, variability and trends in Tanzania to be observed at multiple temporal and spatial scales (e.g. by month, season, year, grid point, district province, country).

8.3 The Climate Suitability for Malaria Transmission Tool (CSMT)

The Climate Suitability for Malaria Transmission Tool (CSMT) [69] is an interactive mapping tool that interrogates the ENACTS database and then displays the number of months during the year when climatological (i.e. long-term average) conditions are considered to be suitable for malaria transmission. Suitability is based on empirically-derived thresholds of precipitation, temperature and relative humidity. These are a) monthly precipitation accumulation is at least 80 mm, b) monthly mean temperature is between 18°C and 32°C (*P. falciparum*) 16°C and 32°C (*P. vivax*)

and c) monthly relative humidity is at least 60%. In practice, the optimal and limiting conditions for transmission are dependent on local conditions (including surface water) and the particular species of the parasite and vector.

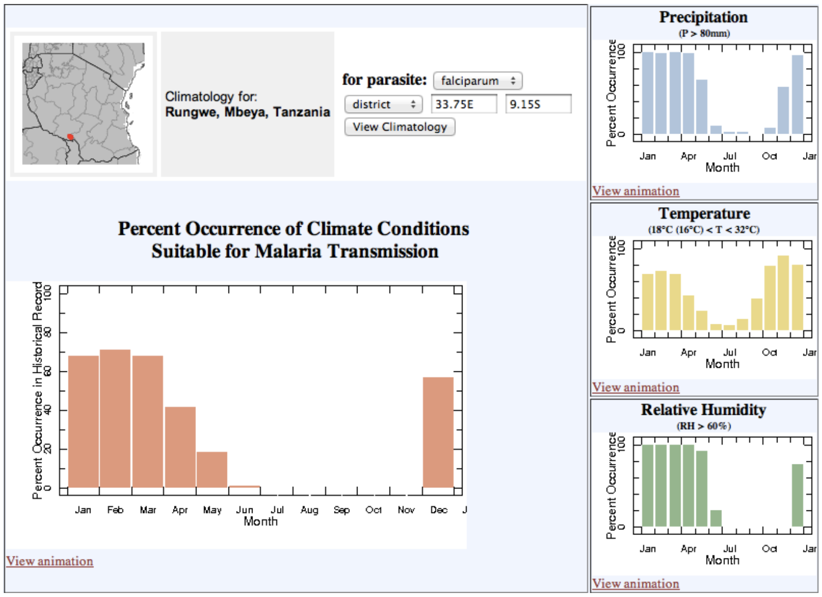

Figure 8-4 CSMT *P. falciparum* for Rungwe District, Mbeya, Southern Tanzania

Where ever the CSMT tool indicates less than 100% occurrence of a climate suitable for malaria transmission throughout the entire year the region is likely (at least in part) to be highly sensitive to climate variability and trends – for example Rungwe District Figure 8-4 .

8.4 Baselines

Central to malaria intervention impact assessment is the concept of a baseline year or baseline period against which changes in outcomes can be measured. If the climate risk for malaria in the baseline period was unusually severe then achieving change relative to that baseline is relatively easy. Using the Weighted Average Standardized Precipitation Tool (WASP) it is possible to explore changes in rainfall integrated over time and over a specified region for both a baseline and intervention period. Where temperature is not a constraint to malaria transmission this tool may provide a good estimate of climate risk for malaria. Figure 8-5 shows a WASP calculation for Tanzania using a baseline period of (1995-1999) and an intervention period (2000-2010).

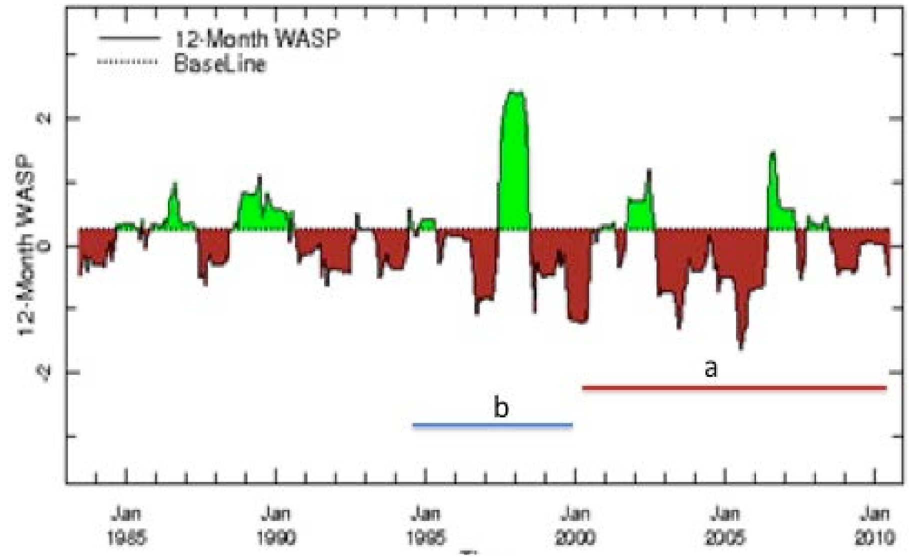

Figure 8-5 Indicates that in Tanzania the intervention years (2000-2010) included major droughts (2000, 2004-2006) while the baseline period included a major El Nino year (1997/8).

Additional WASP analysis for each district in Tanzania can be seen in Figure 8-.

The climate analysis tool permits exploration of both rainfall and temperature. Warming over the last 28 years in Tanga is very apparent, as is the extreme rainfall in 1997-1998.

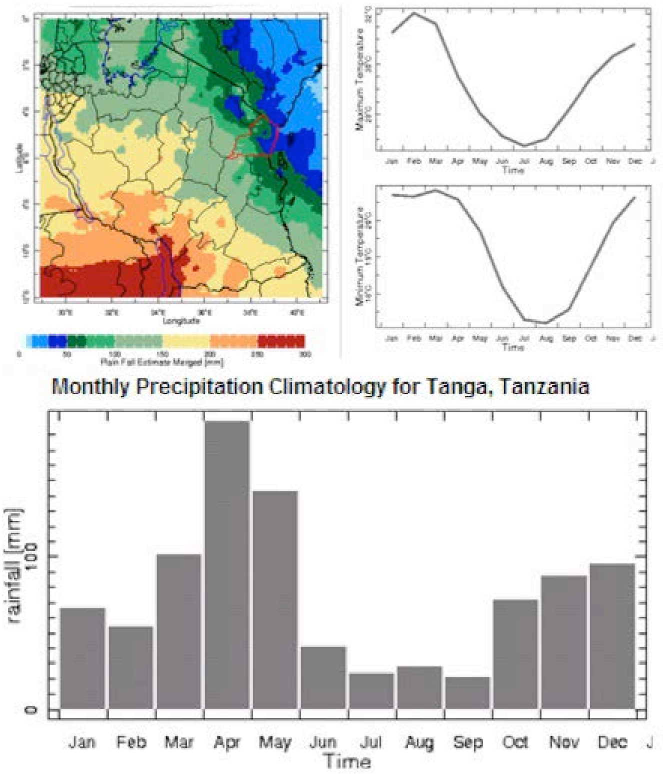

Figure 8-6 Tanga District - trends in rainfall (top) max T (middle) min T (bottom)

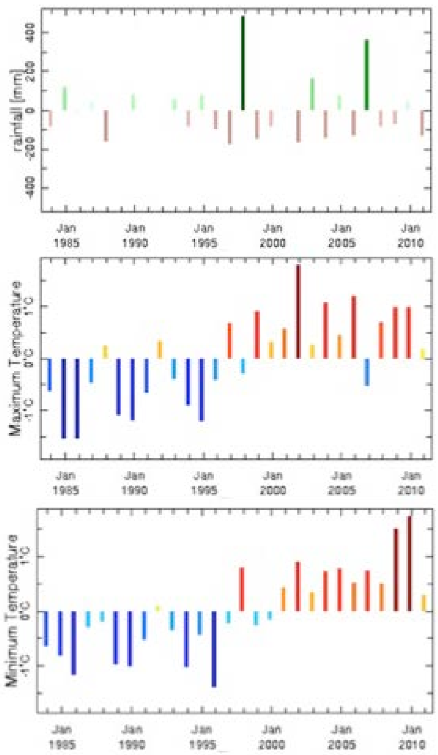

Figure 8-7 Tanga District - trends in rainfall (top) max T (middle) min T (bottom) during Oct-Dec.

8.5 Findings from Analysis

From the analysis we have undertaken the following conclusions can be drawn

1. The new ENACTS product for Tanzania provides a high quality climate database which is suitable for analysis at different time and space scales (e.g. district and country, month, season, year). However, malaria incidence data and intervention data that are suitable for a national statistical analysis using Method 2 was not available to this project.

2. Drought has persisted in Tanzania through much of the last decade. However there is no evidence that drought could account for any substantial decline in malaria 2006-2010 across the whole country as this period is generally wetter than 2000-2005 – as has been observed in other regions of East Africa such as Ethiopia. However, variability at the subnational scale indicates that more detailed analysis will be required for specific provinces and districts.

3. There is evidence of significant warming in many but not all regions of Tanzania including Tanga and other highland areas. Warming appears to follow global sea surface temperatures and is inversely related to rainfall but the response of minimum and maximum temperature is not the same. This warming is highly significant (approx. $0.3^{o}C$ per decade and could (amongst other factors) account for increases in malaria observed at higher altitudes.

4. Analysis of both rainfall and temperature indicates a strong relationship to ENSO in many but not all regions.

8.6 In summary

In Tanzania the relationship between malaria and climate is significant and varies across the country with temperature important in highland areas in the northwest and south central regions and rainfall may be significant across the country especially in the semi-arid regions. As a consequence there is a high risk that impact assessments will be confounded by climate in certain regions. In these areas analysis of malaria and climate must be undertaken at multiple scales to account for local complexity.

Figure 8-8: WASP analysis for Tanzania's districts using a 1995-2000 baseline

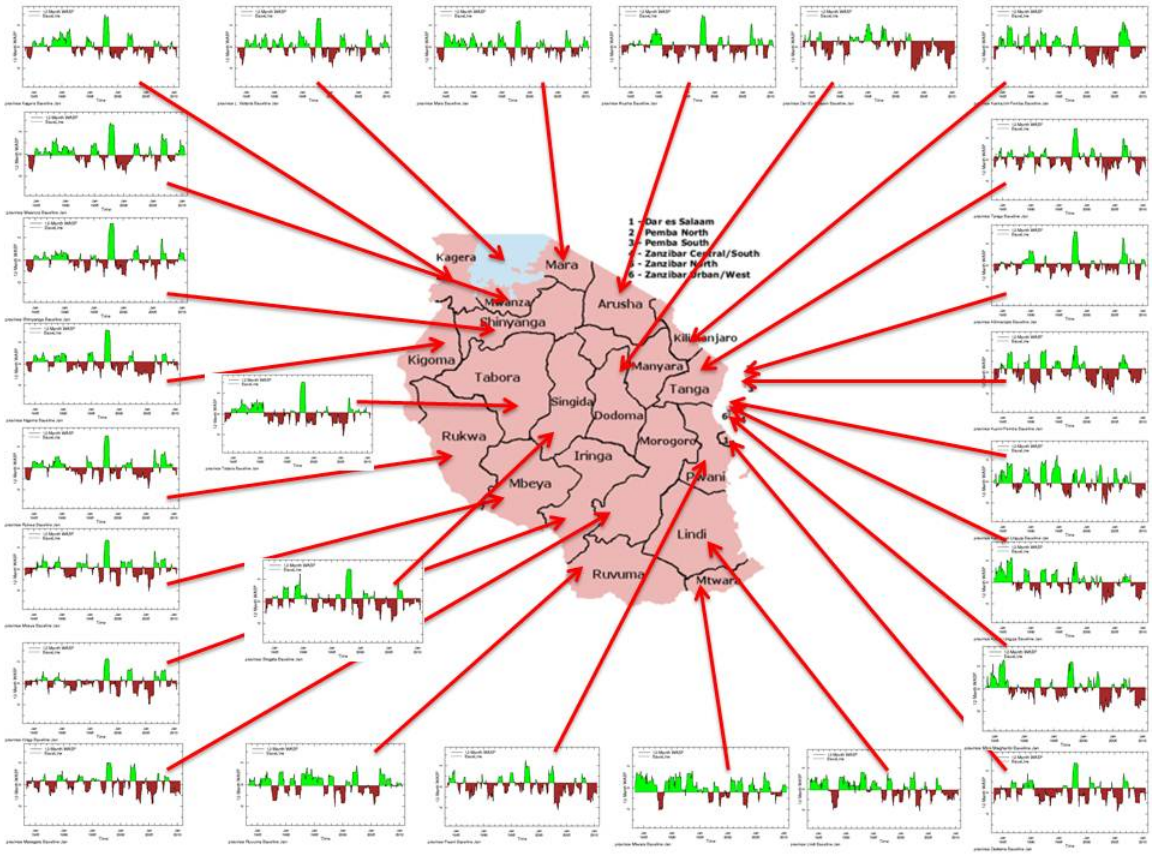

9 Conclusion

In the context of impact assessments of malaria interventions, the risk of climate confounding the assessments can be described as either elevated, relative to a baseline period, (e.g. during a period of drought in a semi-arid area) or depressed, relative to a baseline period (e.g. during a warmer and wetter period in a highland region). The following matrix Table 4 indicates the impact of ignoring climate under differing scenarios of climate suitability and malaria pre and post intervention.

Table 4 Possible outcomes if climate information is not incorporated into malaria impact assessment:

	**Malaria decreases following intervention**	**Malaria remains the same or increases following intervention**
**Climate suitability for malaria transmission increases following intervention**	Failure to incorporate climate in analysis may underestimate benefits of intervention	Failure to incorporate climate in analysis may result in resurgence being blamed inappropriately on non-climatic factors or conversely climate being blamed for resurgence when in fact control failure is responsible
**Climate suitability for malaria transmission does not change following intervention**	No further climate analysis required	No further climate analysis required
**Climate suitability for malaria transmission decreases following intervention**	Failure to incorporate climate in analysis may overestimate benefits of intervention	Failure to incorporate climate may underestimate the importance of non-climatic factors in driving malaria increase.

9.1 Recommendations

- ENACTS climate information products are developed for all PMI countries
- Ranking tool for each country are developed which indicate the relative importance of climate as a confounder in malaria intervention impact assessment
- Method 1 (Climate Information Analysis) is performed on ENACTS data from all countries deemed climate sensitive
- Method 2 (Climate Information, Malaria and Intervention Analysis) is undertaken in PMI countries wherever malaria is deemed climate sensitive and appropriate data sets are available. Climate data and methodology to be made available to local scientists and those engaged in impact assessment.

- A new product be developed that integrates both rainfall and temperature and can be used in a more sophisticated WASP style analysis to explore pre and post intervention climate suitability.

- ENACTS products are used to investigate the potential predictability of Climate Suitability for Malaria Transmission from global climate processes.

- The risk to current control and future malaria elimination programs of a warmer and drier/warmer and wetter climate for malaria control and elimination to be assessed.

10 References

1. Murray, C., et al., *Global malaria mortality between 1980 and 2010: a systematic analysis.* The Lancet, 2012. **379**(9814): p. 413-431.
2. WHO/UNICEF, *The World Malaria Report 2010*, 2011.
3. Okiro, E.A., et al., *Malaria paediatric hospitalization between 1999 and 2008 across Kenya.* BMC Medicine, 2009. **7**: p. 75.
4. Prudhomme, W., et al., *Changes in the burden of malaria in sub-Saharan Africa.* Lancet Infectious Diseases, 2010. **10**: p. 545-555.
5. Nyarango, P.M., et al., *A steep decline of malaria morbidity and mortality trends in Eritrea between 2000 and 2004: the effect of combination of control methods. .* Malaria Journal, 2006. **5**(33).
6. Ceesay, S., et al., *Changes in malaria indices between 1999 and 2007 in The Gambia: a retrospective analysis.* The Lancet, 2008. **372**(9649): p. 1545-1554.
7. O'Meara, W., et al., *Changes in the burden of malaria in sub-Saharan Africa.* Lancet Infectious Diseases, 2010. **10**(8): p. 545-55.
8. Aregawi, M.W., et al., *Reductions in malaria and anaemia case and death burden at hospitals following scale-up of malaria control in Zanzibar, 1999-2008.* Malaria Journal, 2011. **10**(46).
9. Roca-Feltrer, A., et al., *Lack of Decline in Childhood Malaria, Malawi, 2001–2010.* Emerging Infectious Diseases, 2012. **18**(2).
10. RBM, *A decade of partnership and results*, in *Progress and Impact Series*2011, RBM. p. 136pp.
11. Rowe, A.K., et al., *Viewpoint: evaluating the impact of malaria control efforts on mortality in sub-Saharan Africa; Roll Back Malaria Monitoring and Evaluation Reference Group.* Tropical Medicine & International Health, 2007. **12**(12): p. 1524-39.
12. Simon, J., et al., *External Evaluation of the President's Malaria Initiative - Final Report*, 2011: Washington, D.C.
13. Meyrowitsch, D.W., et al., *Is the current decline in malaria burden in sub-Saharan Africa due to a decrease in vector population?* Malaria Journal, 2011. **10**: p. 188.
14. Lindsay, S.W., L. Parson, and C.J. Thomas, *Mapping the ranges and relative abundance of the two principal African malaria vectors, Anopheles gambiae sensu stricto and An.arabiensis, using climate data.* The Royal Society, London. Series B., 1998. **265**: p. 847-854.
15. Bayoh, M.N., et al., *Anopheles gambiae: historical population decline associated with regional distribution of insecticide-treated bed nets in western Nyanza Province, Kenya.* Malaria Journal, 2010. **9**: p. 62.
16. Jaenisch, T., et al., *Malaria incidence and prevalence on Pemba Island before the onset of the successful control intervention on the Zanzibar Archipelago.* Malaria Journal, 2010. **9**: p. 32.
17. Graves, P.M., et al., *Effectiveness of malaria control during changing climate conditions in Eritrea, 1998-2003.* Tropical Medicine & International Health, 2008. **13**(2): p. 218-228.
18. Creasey, A., et al., *Eleven years of malaria surveillance in a Sudanese village highlights unexpected variation in individual disease susceptibility and outbreak severity.* Parasitology, 2004. **129**: p. 263-271.

19. IRI, *A Gap Analysis for the Implementation of the Global Climate Observing System Programme in Africa*, 2006, International Research Institute for Climate and Society: Palisades, NY.
20. Connor, S.J., et al., *Health and Climate - Needs.* Procedia Environmental Sciences Volume 1, 2010. **1**: p. 27-36.
21. Wernsdorfer, W.H. and I.A. McGregor, *Malaria: Principles and practice of malariology*1988, Edinburgh: Churchill Livingstone.
22. Molineaux, L., *The epidemiology of human malaria as an explanation of its distribution, including some implications for its control*, in *Malaria: Principles and practise of malariology*, W.H. Wernsdorfer and I.J. McGregor, Editors. 1988, Churchill Livingstone. p. 913-998.
23. Worrall, E., A. Rietveld, and C. Delacollette, *The burden of malaria epidemics and cost-effectiveness of interventions in epidemic situations in Africa.* American Journal of Tropical Medicine and Hygiene, 2004. **71(Suppl 2)**: p. 136-140.
24. Thomson, M.C., et al., *Rainfall and Sea-Surface temperature monitoring for malaria early warning in Botswana.* American Journal of Tropical Medicine and Hygiene, 2005. **73**(1): p. 214-221.
25. Thomson, M.C., et al., *Use of rainfall and Sea Surface Temperature monitoring for Malaria Early Warning in Botswana.* American Journal of Tropical Medicine and Hygeine, 2005. **73**(1): p. 214-221.
26. WHO, *Malaria Early Warning Systems, Concepts, Indicators, and Partners: A Framework for Field Research in Africa*, 2001, WHO/Roll Back Malaria/Technical Support Network for Prevention and Control of Malaria: Geneva.
27. Barnish, G., et al., *Malaria in a Rural Area of Sierra-Leone .1. Initial Results.* Annals of Tropical Medicine and Parasitology, 1993. **87**(2): p. 125-136.
28. Cox, J., et al., *Mapping malaria risk in the highlands of Africa - MARA/HIMAL Technical Report*, 1999, MARA / London School of Hygiene and Tropical Medicine: Durban, South Africa / London.
29. Thomson, M.C., et al., *Malaria early warnings based on seasonal climate forecasts from multi-model ensembles.* Nature, 2006. **439**: p. 576-579.
30. Brown, V., et al., *Epidemic of malaria in north-eastern Kenya.* Lancet, 1998. **352**(9137): p. 1356-1357.
31. Vancutsem, C., et al., *Evaluation of MODIS Land surface temperature data to estimate air temperature in different ecosystems over Africa.* Remote Sensing of Environment, 2010. **114**: p. 449-465.
32. Kiszewski, A., et al., *A global index representing the stability of malaria transmission.* American Journal of Tropical Medicine and Hygiene, 2004. **70**(5): p. 486-498.
33. Moshkovsky, S.D. and M.G. Rashina, *Epidemiology and medical parasitology for entomologists*, 1951: Moscow.
34. !!! INVALID CITATION !!!
35. Patz, J.A. and S.H. Olson, *Climate Change and Health: Global to Local Influences on Disease Risk.* Annals of Tropical Medicine and Parasitology, 2006. **100**(5): p. 535-549.
36. Lindsay, S.W. and M.H. Birley, *Climate change and malaria transmission.* Annals of Tropical Medicine and Parasitology, 1996. **90**(6): p. 573-588.
37. Afrane, Y.A., et al., *Effects of microclimate changes caused by land use and land cover on duration of gonotrophic cycles of Anopheles gambiae (Diptera: Culicidae) in western Kenya highlands.* Journal of Medical Entomology, 2005. **42**: p. 974-980.

38. Munga, S., et al., *Association between land cover and habitat productivity of malaria vectors in western Kenyan highlands.* American Journal of Tropical Medicine and Hygiene, 2006. **74**(1): p. 69-75.
39. Tuno, N., et al., *Survivorship of Anopheles gambiae sensu stricto (Diptera : Culicidae) larvae in western Kenya highland forest.* Journal of Medical Entomology, 2005. **42**(3): p. 270-277.
40. McMichael, A.J., et al., *Climate Change and Human Health*1996, Geneva: World Health Organisation.
41. Kovats, R.S., et al., *El Nino and health.* Lancet, 2003. **362**(9394): p. 1481-1489.
42. Nicholson, S., *A Review of Climate Dynamics and Climate Variability in Eastern Africa.* , in *Limnology, Climatology and Paleoclimatology of the East African Lakes*, T.C.O. Johnson, E.O., Editor 1996, Gordon and Breach: New York.
43. Goddard, L., et al., *Providing Seasonal-to-Interannual Climate Information for Risk Management and Decision Making*, in *White Paper for WCC3*2009, World Climate Conference - 3
44. Hay, S.I., et al., *Climate change and the resurgence of malaria in the East African highlands.* NATURE, 2002. **415**(6874): p. 905-909.
45. Patz, J.A., et al., *Climate change (Communication arising): Regional warming and malaria resurgence.* Nature, 2002. **420**: p. 627-628.
46. Pascual, M., et al., *Malaria resurgence in the East African Highlands: Temperature trends revisited. .* Proceedings of the National Academy of Sciences,, 2006. **103**: p. 5829-5834.
47. Christy, J.R., W.B. Norris, and R.T. McNider, *Surface Temperature Variations in East Africa and Possible Causes.* Journal of Climate, 2009. **22**(33423356).
48. Omumbo, J., et al., *Raised temperatures over the Kericho tea estates: revisiting the climate in the East African highlands malaria debate.* Malaria Journal, 2011. **10:12**.
49. Rogers, D.J., et al., *Satellite imagery in the study and forecast of malaria.* Nature, 2002. **415**((6872)): p. 710-715.
50. Thomson, M.C., et al., *Africa needs climate data to fight disease.* Nature, 2011(471): p. 440-442.
51. Huffman, G.J., et al., *Improving the global precipitation record: GPCP Version 2.1.* Geophysical Research Letters, 2009. **36**(L17808).
52. Lyon, B. and D.G. DeWitt, *A recent and abrupt decline in the East African long rains.* Geophysical Research Letters, 2012. **39**.
53. Williams, A.P. and C. Funk, *A westward extension of the warm pool leads to a westward extension of the Walker circulation, drying eastern Africa.* Climate Dynamics, 2011. **37**: p. 2417-2435.
54. Giannini, A., R. R. Saravanan, and P. Chang, *Oceanic Forcing of Sahel Rainfall on Interannual to Interdecadal Time Scales.* Science., 2003(1089357): p. 3-10.
55. Mouchet, J., et al., *Drought and malaria retreat in the Sahel, West Africa.* Lancet, 1996. **348**(9043): p. 1735-1736.
56. Labbo, R., et al., *Anopheles funestus in Sahel: new evidence from Niger.* Lancet, 2004. **363**(9409): p. 660-660.
57. IPCC, ed. *Climate Change 2007: Impacts, Adaptation and Vulnerability. Contribution of Working Group II to the Fourth Assessment Report of the Intergovernmental Panel on Climate Change*. ed. M.L. Parry, et al.2007, Cambridge University Press: Cambridge, UK.
58. Cibulskis, R.E., et al., *Worldwide Incidence of Malaria in 2009: Estimates, Time Trends, and a Critique of Methods. .* PLoS Med, 2011. **8**(12): p. e1001142.

59. Dinku, T., et al., *Validation of satellite rainfall products over East Africa's complex topography.* International Journal of Remote Sensing, 2007. **28**(7-8): p. 1503-1526.
60. Dinku, T., et al., *Improving availability, access and use of climate information* World Meteorological Bulletin, 2011. **60**(2).
61. Roca-Feltrer, A., et al., *A simple method for estimating malaria seasonality.* Malaria Journal, 2009. **8**: p. 276.
62. Craig, M.H., R.W. Snow, and D. le Sueur, *A climate-based distribution model of malaria transmission in sub-Saharan Africa.* Parasitology Today, 1999. **15**(3): p. 105-111.
63. Grover-Kopec, E., et al., *An online operational rainfall-monitoring resource for epidemic malaria early warning systems in Africa.* Malaria Journal, 2005. **4**(6).
64. Gillies, H.M. and D.A. Warrell, *Bruce Chwatt's Essential Malariology*. 3rd ed1996, London: Arnold.
65. Barat, L., *Four malaria sucess stories: how malaria burden was successfully reduced in Brazil, Eritrea, India and Vietnam.* American Journal of Tropical Medicine and Hygiene, 2006. **74**(1): p. 12-16.
66. Nyarango, P.M., et al., *A steep decline of malaria morbidity and mortality trends in Eritrea between 2000 and 2004: the effect of combination of control methods.* Malaria Journal, 2006. **5**: p. 33.
67. Ceccato, P., et al., *Malaria stratification, climate, and epidemic early warning in Eritrea.* American Journal of Tropical Medicine and Hygiene, 2007. **77**(6): p. 61-68.
68. Fontaine, R.E., A.E. Najjar, and J.S. Prince, *The 1958 malaria epidemic m Ethiopia.* American Journal of Tropical Medicine and Hygiene, 1961. **10**: p. 795-803.
69. Grover-Kopec, E., et al., *Web-Based Climate Information Resources for Malaria Control in Africa.* Malaria Journal, 2006. **5**: p. 38.
70. Wort, U.U., et al., *Impact of El Nino and malaria on birthweight in two areas of Tanzania with different malaria transmission patterns.* International Journal of Epidemiology, 2004. **33**(6): p. 1311-1319.
71. Lindsay, S.W., et al., *Effect of 1997-98 El Nino on highland malaria in Tanzania.* Lancet, 2000. **355**(9208): p. 989-990.

11 Appendix I: Workshop report "The use of Climate Information in Impact Assessment for Malaria Interventions" Addis Ababa, Ethiopia

To test the acceptability of the ENACTS products to a key stakeholder community the workshop, 'Use of Climate information in Impact Assessment for Malaria Interventions', was held with support from the Federal Ministry of Health and the National Meteorological Services Agency of Ethiopia, at the UNECA Conference Center, in Addis Ababa, Ethiopia from December 12-14, 2011. The main objectives of the workshop were to: 1. Introduce the ENACTS products to the Ethiopian Malaria community, 2. Investigate possible associations between climate variability and trends, and malaria transmission in Ethiopia between July 2004 and June 2009 at sub–national levels, using the comprehensive IDSR data set made available by the Ministry of Health, and 3. Establish a methodology for removing the confounding effect of climate on impact evaluation for malaria interventions in Ethiopia using a range of local malaria data sets and the ENACTS product. The workshop was very successful and indicated the strong interest on the part of this user community in accessing and using the ENACTS products.

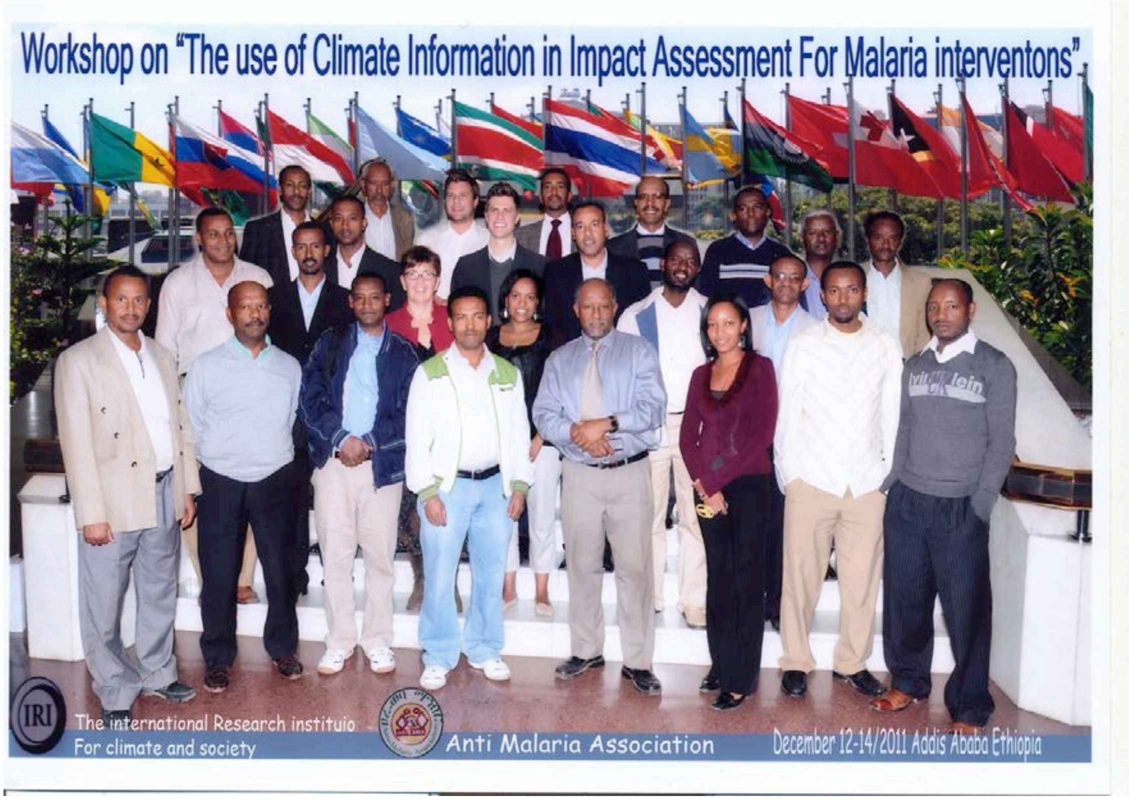

For more information please see the December 2011 'Use of Climate information in Impact Assessment for Malaria Interventions' Workshop report available at the following link: Building Capacity to Produce and Use Climate Information for Improving Health in East Africa

12 Appendix II: Workshop report "Data Quality Control, Satellite Rainfall Estimation, and Merging Station Observations with Satellite Estimates. Dar es Salaam, Tanzania.

The Tanzanian Meteorological Agency (TMA) and the International Research Institute for Climate and Society (IRI) of Columbia University, agreed to work together to improve the availability, access and use of climate information in Tanzania. This would be accomplished by:

1. Generating a 30-year times series of enhanced rainfall and temperature data every 10km grid over Tanzania through combination of all available observations with satellite proxies;
2. Creating new climate analysis and monitoring products for specific applications; and
3. Installing an online mapping service at TMA that will provide user-friendly tools for visualization, querying, and accessing information products.
4.

A critical aspect of the collaboration was to build capacity with a goal of making activities and investments sustainable. TMA staff participated in the first of an intended three training workshops to understand and implement each of the three components of the project. The first training workshop focused on the generation of a 30-year rainfall and temperature time series. This two-week training included:

- Quality control of station data;
- Introduction to satellite rainfall estimation; and
- Combining satellite data with station measurements.

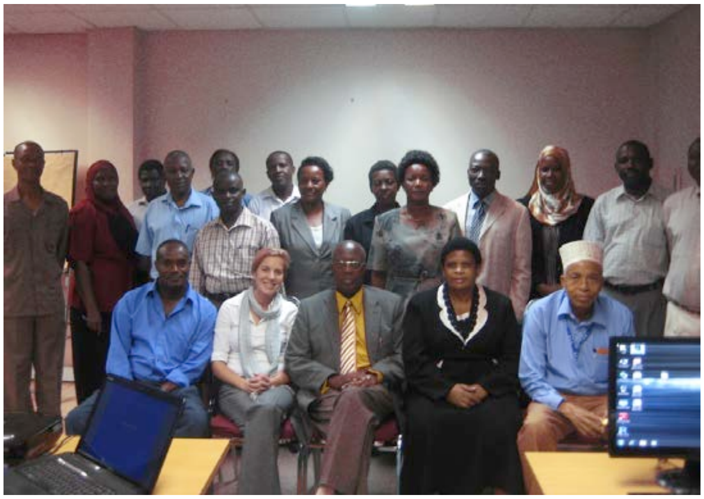

Workshop participants

For more information please see the Workshop report available at the following link: Data Quality Control, Satellite Rainfall Estimation, and Merging Station Observations with Satellite Estimates. Dar es Salaam, Tanzania (username: PMI, password: pietro)
