# Supplementary figures and images for: Impact of Malaria Control on Mortality and Anemia among Tanzanian Children Less than Five Years of Age, 1999–2010
Source: PLoS One. 2015 Nov 4;10(11):e0141112. doi: 10.1371/journal.pone.0141112 (PMC4633136; doi:10.1371/journal.pone.0141112)

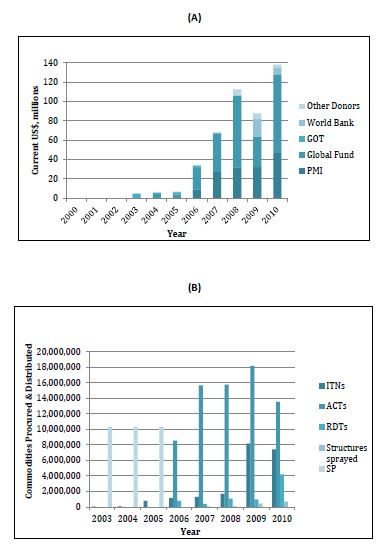

Supplement: S1 Fig — Footnote: *GoT = Government of Tanzania; PMI = U.S. President’s Malaria Initiative (TIF) [file pone.0141112.s002.tif]

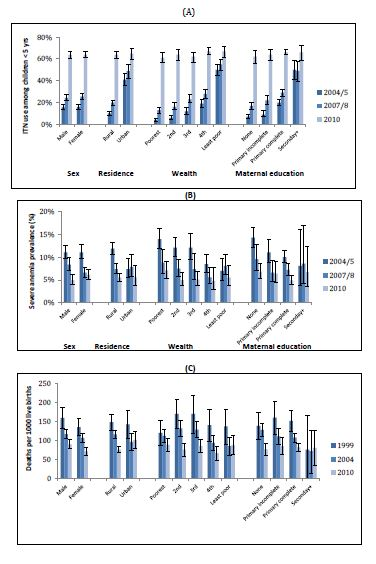

Supplement: S2 Fig — (TIF) [file pone.0141112.s003.tif]

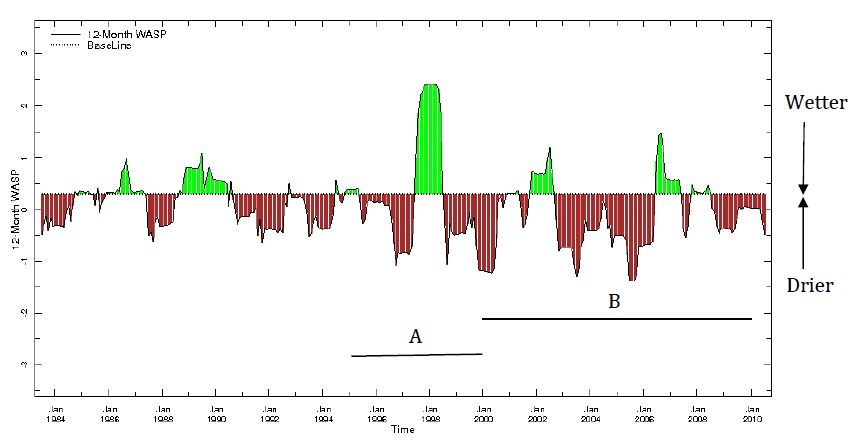

Supplement: S3 Fig — Footnote: red indicates drier, green indicates wetter (compared to baseline) (TIF) [file pone.0141112.s004.tif]
